# Refining colorectal cancer classification and clinical stratification through a single-cell atlas

**DOI:** 10.1186/s13059-022-02677-z

**Published:** 2022-05-11

**Authors:** Ateeq M. Khaliq, Cihat Erdogan, Zeyneb Kurt, Sultan Sevgi Turgut, Miles W. Grunvald, Tim Rand, Sonal Khare, Jeffrey A. Borgia, Dana M. Hayden, Sam G. Pappas, Henry R. Govekar, Audrey E. Kam, Jochen Reiser, Kiran Turaga, Milan Radovich, Yong Zang, Yingjie Qiu, Yunlong Liu, Melissa L. Fishel, Anita Turk, Vineet Gupta, Ram Al-Sabti, Janakiraman Subramanian, Timothy M. Kuzel, Anguraj Sadanandam, Levi Waldron, Arif Hussain, Mohammad Saleem, Bassel El-Rayes, Ameen A. Salahudeen, Ashiq Masood

**Affiliations:** 1 Indiana University School of Medicine, Indianapolis, IN USA; 2 Isparta University of Applied Sciences, Isparta, Turkey; 3 Northumbria University, Newcastle Upon Tyne, UK; 4 Yildiz Technical University, Istanbul, Turkey; 5 Rush University Medical Center, Chicago, IL USA; 6 Tempus Labs, Inc., Chicago, IL USA; 7 The University of Chicago, Chicago, IL USA; 8 Inova Schar Cancer Institute, Fairfax, VA USA; 9 Institute of Cancer Research, London, UK; 10 CUNY Graduate School of Public Health and Health Policy, New York, NY USA; 11 University of Maryland Marlene and Stewart Greenebaum Comprehensive Cancer Center, Baltimore, MD USA; 12 University of Alabama, O’Neil Comprehensive Cancer Institute, Birmingham, AL USA

**Keywords:** Cancer-associated fibroblast, CMS classification, Colorectal cancer, Single-cell analysis, Immunotherapy, Stromal signatures

## Abstract

**Background:**

Colorectal cancer (CRC) consensus molecular subtypes (CMS) have different immunological, stromal cell, and clinicopathological characteristics. Single-cell characterization of CMS subtype tumor microenvironments is required to elucidate mechanisms of tumor and stroma cell contributions to pathogenesis which may advance subtype-specific therapeutic development. We interrogate racially diverse human CRC samples and analyze multiple independent external cohorts for a total of 487,829 single cells enabling high-resolution depiction of the cellular diversity and heterogeneity within the tumor and microenvironmental cells.

**Results:**

Tumor cells recapitulate individual CMS subgroups yet exhibit significant intratumoral CMS heterogeneity. Both CMS1 microsatellite instability (MSI-H) CRCs and microsatellite stable (MSS) CRC demonstrate similar pathway activations at the tumor epithelial level. However, CD8+ cytotoxic T cell phenotype infiltration in MSI-H CRCs may explain why these tumors respond to immune checkpoint inhibitors. Cellular transcriptomic profiles in CRC exist in a tumor immune stromal continuum in contrast to discrete subtypes proposed by studies utilizing bulk transcriptomics. We note a dichotomy in tumor microenvironments across CMS subgroups exists by which patients with high cancer-associated fibroblasts (CAFs) and C1Q+TAM content exhibit poor outcomes, providing a higher level of personalization and precision than would distinct subtypes. Additionally, we discover CAF subtypes known to be associated with immunotherapy resistance.

**Conclusions:**

Distinct CAFs and C1Q+ TAMs are sufficient to explain CMS predictive ability and a simpler signature based on these cellular phenotypes could stratify CRC patient prognosis with greater precision. Therapeutically targeting specific CAF subtypes and C1Q + TAMs may promote immunotherapy responses in CRC patients.

**Supplementary Information:**

The online version contains supplementary material available at 10.1186/s13059-022-02677-z.

## Background

Colorectal cancer (CRC) is the third most common cancer in the world and the leading cause of cancer-related mortality [1]. Approximately one third of patients experience disease relapse following curative-intent surgical resection and chemotherapy [2, 3]. Despite the high prevalence and mortality of advanced CRC, only a few treatments have been approved in indications for a small subset of CRC patients, such as immune checkpoint inhibitors in microsatellite unstable (MSI-H) tumors and combined EGFR/BRAF inhibitors in BRAF V600E mutant CRCs [4, 5]. The molecular heterogeneity of CRC and its variable clinical course hinder the advancement of effective therapeutics and present considerable challenges in accurately evaluating prognostic and predictive indicators. Although The Cancer Genome Atlas (TCGA) has established the somatic mutational landscape within CRC, numerous studies have shown that stromal and immune signatures, such as fibroblasts and cytotoxic T cells may be the key drivers of clinical outcomes [6–9]. These results indicate that a diverse niche of heterotypic cell interactions inside the tumor microenvironment (TME) governs its tumor biology, and consequently its clinical phenotypes of CRC and, consequently, its tumor biology.

Many groups have proposed CRC subtypes based on large-scale gene expression studies. The International Consortium published the consensus molecular subtypes (CMS), which classified CRC as CMS1 (MSI immune), CMS2 (canonical), CMS3 (metabolic), and CMS4 (mesenchymal) based on bulk transcriptomic signatures [10]. However, all CRC classifications, including CMS classification, relied on data acquired through bulk sequencing, which inherently lacks the resolution to probe CRC tumors and their complex microenvironment at the cellular level necessary to detect molecular signatures in small yet critical cell populations. This has been demonstrated in numerous bulk expression studies in which stromal cells conceal essential signals emanating from other major cellular phenotypes within the CRC spectrum, influencing CRC classifications [11–13]. In addition, the potential clinical implications of intratumoral CMS heterogeneity have been suggested by several recent retrospective studies [14–16].

The only prospective study to date that used the CMS classification (specifically the CMS4 subtype) for patient selection based on dual PD-L1/TGF-ß expression signatures was halted due to futility, implying that CMS does not fully reflect the biological diversity of colorectal cancer [17]. Thus, CMS should be used as a starting point to further CRC biology research in order to develop novel biomarkers and rational combinatorial therapies. More recently, single-cell studies in CRC have attempted to provide a global view of the CRC landscape [18, 19]; however, in-depth systematic characterization of how cells of tumor and TME shape the tumor, stromal and immune landscape leading to specific CRC subtypes has not been completely characterized to date.

To provide additional insights into how cellular populations of tumor epithelia, stroma and immune cells shape the CRC landscape, we utilized single-cell RNA sequencing (scRNA-seq) and confirmed our findings in additional three independent single-cell datasets with advanced computational analysis on CRC tumors. Our study enabled us to answer several outstanding questions in CRC disease pathogenesis, including uncovering the unique tumor cell-intrinsic features that impact immune and stromal cell infiltration in each CMS group, the role of cells constituting the tumor microenvironment in each CMS at single-cell resolution, and identified cell populations including distinct Cancer-associated fibroblasts (CAFs) and immunosuppressive tumor-associated macrophages (TAMs) subtype driving clinical outcomes. We uncovered various cell populations in CRC tumors that could be exploited as therapeutic targets for drug development.

## Results

**Fig. 1:**
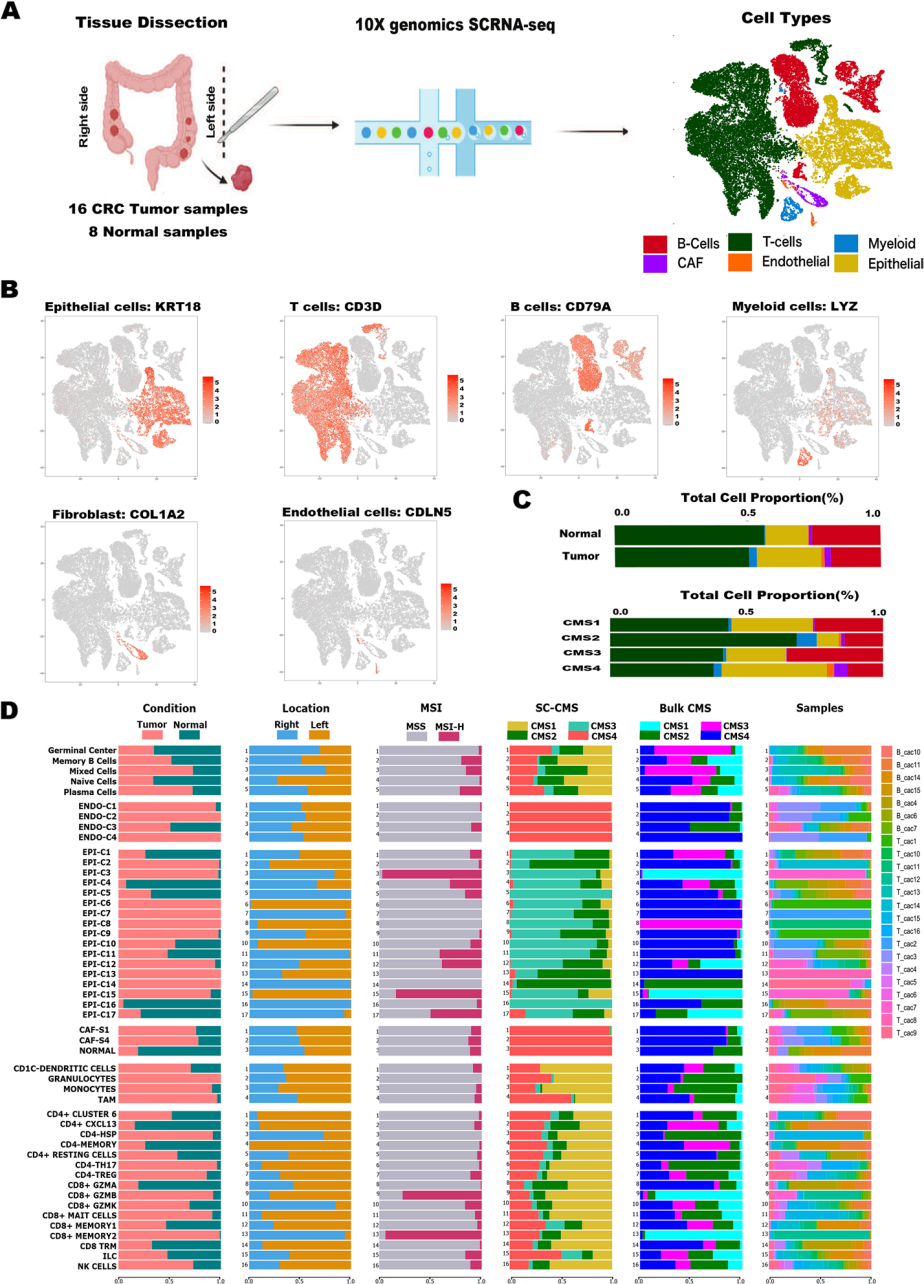
Identification and clustering of single cells. **A** Workflow of sample collection, sorting, and sequencing (methods contain full description for each step) and t-SNE characterization of the 49,859 cells profiled. **B** Identification of various cell types based on expression of specified marker genes. **C** Characterization of the proportion of cell types identified in each sample in tumor vs. normal colon tissue and Consensus Molecular Subtypes (CMS) of bulk RNA-seq data. **D** Characterization of the proportion of cell types identified in tumor vs. normal colon tissue, sidedness (right vs. left), microsatellite instability (MSI) status, single-cell Consensus Molecular Subtypes (scCMS) classification, Consensus Molecular Subtypes (CMS) of bulk RNA-seq data, and origin of sample. The graph represents total clusters and cell types identified after re-clustering of each cell compartment depicting global heterogeneous landscape of colorectal cancers

To determine and dissect the extent of tumor, immunological, and stromal heterogeneity in CRC patients, we performed droplet-based scRNA-seq on 16 racially diverse, treatment naïve CRC patient tissue samples and seven adjacent normal colonic tissue samples (totaling 23 samples) (Fig. 1A–D, Additional file 1: Fig. S1, Additional files 2, 3 and 4: Tables S1–S3). Stringent quality control yielded 49,589 high-quality, single cells for further analysis (Fig. 1A). Graph-based clustering of merged and normalized cells identified robust, discrete clusters of epithelial cells (*EPCAM+*, *KRT8+*, and *KRT18+*), fibroblasts (*COL1A1+*), endothelial cells (*CLDN5+*), T cells (*CD3D+*), B cells *(CD79A+*), and myeloid cells (*LYZ+*) based on canonical marker genes (Fig. 1B). Clusters expressing hybrid cell markers were manually removed from further analysis (see Methods). To deconstruct the molecular makeup of the tumor and TME for each CMS, we evaluated each cell type independently and identified subpopulations with diverse functional roles. Interestingly, re-clustering of major compartments individually also detected clusters expressing hybrid markers, as well as cell clusters expressing markers from distinct lineages (such as T cell clusters expressing B cell markers); these were manually removed and excluded from the downstream analysis. Clustree (V0.4.1) and manual review of differentially expressed genes in each subcluster were studied to choose the best cluster resolution without cluster destabilization (see Methods). Cell population designation was chosen by specific gene expression, and SingleR and Bioturing were also utilized for unbiased cell type recognition [20–24] (see Methods). Taken together, these steps allowed us to retain high-quality single-cell data. In parallel, we performed bulk RNA-seq analysis of the same samples to classify the CMS of each tumor (see Methods). This study revealed a complex cellular ecosystem made up of 49 distinct immune, stromal, and cancer-cell subclusters (Fig. 1D). The tumor cells were mostly clustered by patient, which confirms the significant interpatient heterogeneity observed in previous studies [18, 19, 25, 26]. Cells from both the stromal and immune compartments, on the other hand, were clustered by cell type clusters suggesting a limited batch effect (Additional file 1: Fig. S1).

To validate our findings, we profiled three independent CRC datasets [18, 19, 27] using similar quality metrics as that of primary CRC data to retain only high-quality cell phenotypes (Additional file 1: Figs. S2–S7). All datasets identified similar cell populations providing independent validation to our findings. We utilized comparative analysis to compare 18,296 CAF cells from a breast cancer (BC) cohort to CRC cohorts in order to discover the existence of distinct CAF subtypes not previously reported in CRC samples [28].

Interestingly, all CRC samples in four independent datasets showed discordant and wide-ranging cell proportion enrichment irrespective of CMS classification. It is most likely owing to the high prevalence of dropouts associated with single-cell RNA sequencing, capturing only a small fraction of the RNA molecules, as shown in many single-cell studies in diverse tumor types [18, 23, 25, 29–31] (Fig. 1C and D, Additional file 1: Figs. S6–S8). We also noted immune cell enrichment in the majority of the samples, which is consistent with previous findings in other studies indicating that dissociation protocols significantly impact on non-immune cell recovery in droplet based scRNA-seq analysis [32–34]. Furthermore, differences in the sampling procedure or intratumoral CMS heterogeneity could have influenced these results (see below) [19, 27]. In total, we profiled 487,829 single cells in this study.

Additionally, we employed two bulk- gene expression datasets and computed the prevalence of cellular phenotypes using two distinct approaches, including CIBERSORTx, to characterize their prognostic significance and evaluate their contribution to the existing CMS subgroups (see Methods) [35, 36]. Both methodologies yielded comparable results, validating our analysis.

### Tumor epithelial cells exhibit intratumoral CMS heterogeneity and drives immune-stromal cell infiltration

**Fig. 2:**
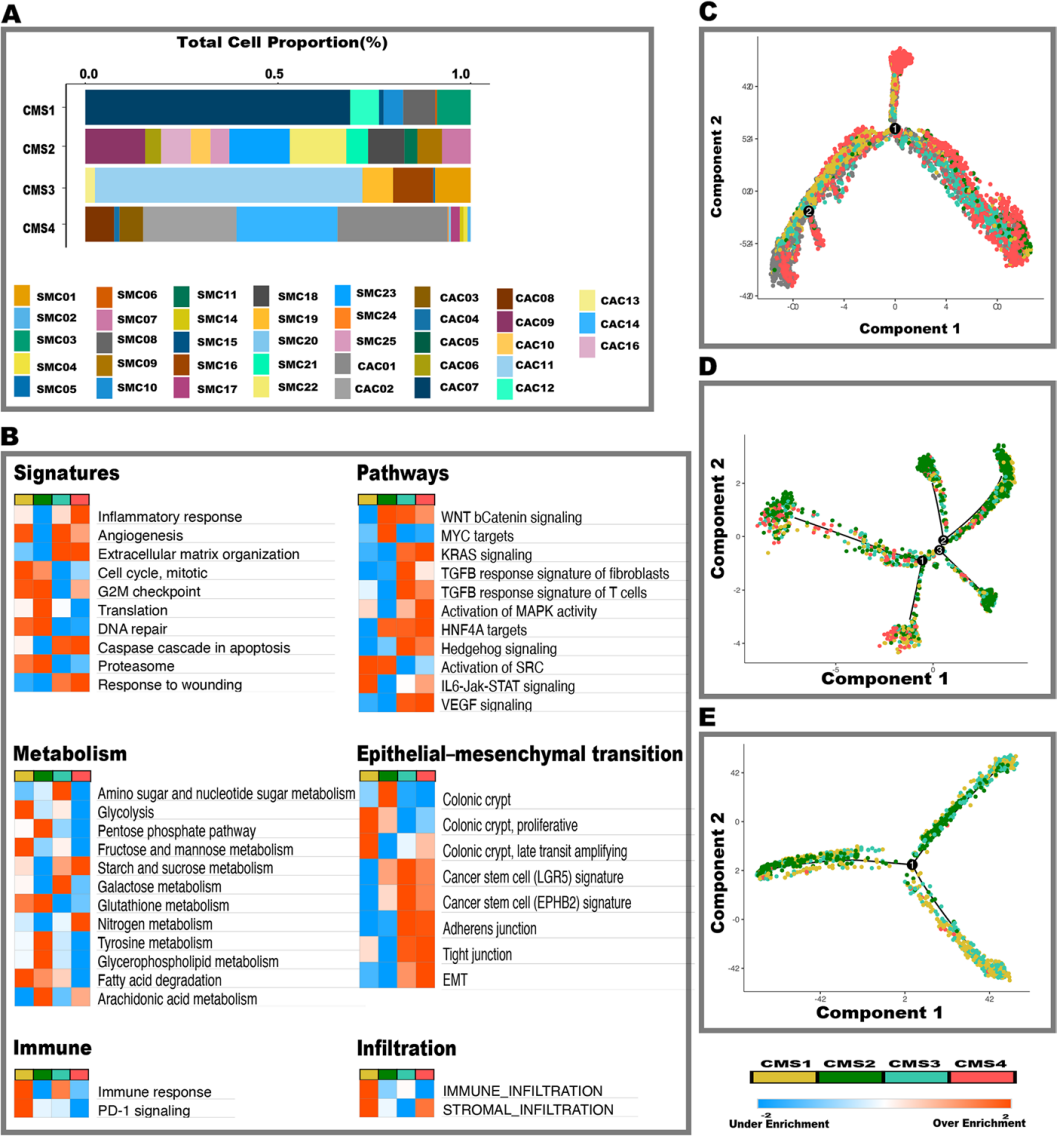
CMS Cell proportions, Gene set enrichment and trajectories of tumor cells. **A** Proportions of tumor cells classified as various CMS subtypes in various samples. Primary CRC datasets is labeled CAC and Lee et al. 2020 [18] dataset labeled as SMC. Annotation of tumor cells is based on bulk CMS classification. **B** Gene set variation expression analyses of combined primary CRC data and Lee et al. 2020 [18], in the tumor epithelial cell compartment. **C** Trajectory analysis of primary CRC dataset colored by bulk CMS status. **D** Trajectory analysis of Lee et al. 2020 [18] (Korean cohort) CRC dataset colored by bulk CMS status. **E** Trajectory analysis of Lee et al. 2020 [18] (Belgian cohort) colored by bulk CMS status

It is currently unknown if cell autonomous or cancer cell intrinsic cancer-cell programs influence stromal and immune infiltration patterns in different CMS groups. To address this critical question, we pooled tumor cells from 38 samples (*N* = 7,530 cells, this study and Lee et al. [18]) that were classified into various CMS subtypes using their matched bulk RNA sequencing data to represent each of the four CMS subgroups appropriately. After batch correction and normalization, we performed pseudo-bulk differential expression analysis, followed by pathway analysis between the CMS utilizing scRNA-seq data (Fig. 2A, B, Additional file 5: Table S4) (see Methods). Notably, there were significant tumor-cell transcriptional differences between CMS groups. CMS1 tumor epithelial cells showed increased enrichment of immunological, proteasome, JAK-STAT and PD-1 signaling pathways, whereas CMS4 tumor epithelial cells displayed epithelial-mesenchymal transition (EMT), VEGF, and TGF-β activation, among other pathways (Fig. 2B) [10]. CMS3 and CMS4 tumor epithelial cells showed unexpected enrichment in the Wnt pathway, with Wnt activity expressed in a decreasing gradient from the crypt base to the differentiated compartment, suggesting CMS subtypes are associated with distinct regions of the colon crypts (Fig. 2B) [37]. CMS2 tumor epithelial cells also showed gene expression driven by copy number alterations in the MYC and DNA repair genes. Further, CMS2 was associated with substantial variability and enrichment of multiple pathways regulating metabolism and the cell cycle, confirming CMS2 is the most heterogeneous among the various CMS subgroups (Fig. 2B) [37, 38]. Also, CMS3 tumor epithelial cells showed KRAS signaling was upregulated at the transcriptomic level in CMS3 which also demonstrated immunological and immune evasion signatures (JAK-STAT signaling), implying this subtype is not entirely immune deficient, consistent with the observation that a subset of MSI tumors are represented by this subtype [10]. CMS4 demonstrated upregulated KRAS signaling, supporting a tumor cell autonomous mechanism of cetuximab resistance in the absence of KRAS gain of function mutations [39].

Even though both MSI-H and MSS CRC tumors are included in the CMS1 subgroup, MSS tumors within this subgroup do not respond to immune checkpoint inhibitors. Therefore, we further investigated CMS1 MSS tumors (Additional file 1: Fig. S9, Additional file 6: Table S5). Intriguingly, we found that CMS1 MSS tumor epithelial cells had similar pathway activation patterns as the MSI-H tumors, particularly with respect to immunological, PD1, and JAK-STAT pathways. This suggests that at the tumor cell level, similar gene expression signatures define the CMS1 tumors independent of their relative microsatellite stability status. However, MSI-H CRC seemed to vary from MSS CRC based on CD8+ cytotoxic T cell infiltration patterns (see below).

Within tumor epithelial cells, multiple key pathways such as angiogenesis, inflammation, WNT pathway activation were shared between the CMS subgroups, which we hypothesize may be due to intratumoral heterogeneity of cellular differentiation/plasticity within the tumor (Fig. 2B). To verify our hypothesis, we applied two independent methods of trajectory analysis (Monocle 2 and Slingshot) to infer potential alignments or lineage relationships (CMS designation) (Fig. 2C, Additional file 1: Fig. S10) [40, 41]. These analyses also served as control for inter-patient heterogeneity and as orthogonal validation for confirming the transcriptomic patterns. Our analysis showed no correlation with respect to CMS classification, underscoring that intratumoral CMS heterogeneity is prevalent among CRC tumors. Our results are contradictory to Lee et al. [18], who showed tumor epithelial cells align along a CMS subgroup trajectory. We re-analyzed their data (Korean cohort) using current best standard practices, and upon excluding low-quality cells, our analysis revealed no CMS alignments within the Lee et al. [18] data (Fig. 2D, Additional file 1: Fig. S10). We also utilized an independent Belgian cohort from the Lee et al. [18], dataset, which also concurred with our findings of intratumoral heterogeneity beyond the CMS classification. Thus, intratumoral CMS heterogeneity occurs across datasets and patients (Fig. 2E, Additional file 1: Fig. S10) [18]. Taken together, our results show that tumor epithelia recapitulated the individual CMS subgroups and added another level of complexity by displaying intratumoral CMS heterogeneity at the single-cell level.

### CAFs in the tumor microenvironment exhibit diverse phenotypes

**Fig. 3:**
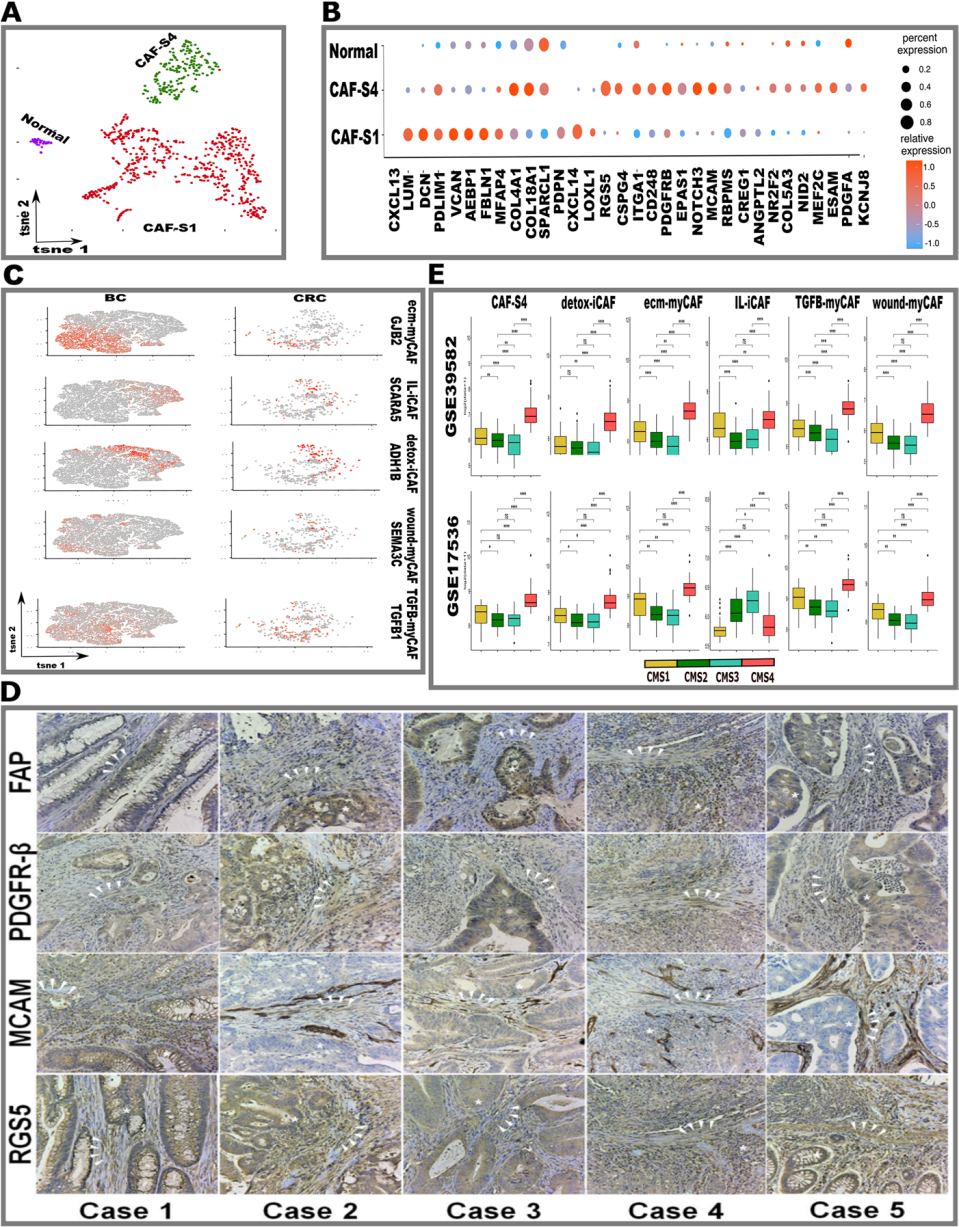
Fibroblast clusters in colon and colorectal tumors. **A** t-SNE of fibroblasts colored by Normal, CAF-S1 and CAF-S4 subtypes. **B** Dot plots showing the variable expression of fibroblast specific marker genes across CAF-S1 and CAF-S4. **C** Integration analysis of five CAFs subtypes from a breast cancer (BC) cohort to validate the existence of specific CAF subtypes in the CRC samples. **D** IHC representative images of CAF-S1 (FAP+, PDGFR-ß+) and CAF-S4 (RGS5+, MCAM+) in CRC sections from five independent patients. Asterisk (*) indicates tumor cells and arrows (>) indicate CAFs. Annotated by a board-certified GI pathologist. All images are × 20 magnification. High-resolution images are available on GitHub as source data [43]. **E** Boxplots show the distribution of cell types in two CRC bulk expression datasets, within tumors based on CMS status. The whiskers depict the 1.5 x IQR. The *P*-values for pairwise *t*-tests comparisons (with Benjamini-Hochberg correction) of cell abundance across CMS are shown in the figure. Note “NS”: *P* > 0.05, *P </=0.05, **P </=0.01, ***P </=0.001, ****P </=0.0001

Recent studies have identified potential heterotypic interactions of cancer-associated fibroblasts (CAF) within the CRC microenvironment [18, 19, 27]. However, CAF heterogeneity and relationship to CMS have not been evaluated at the single-cell level. On re-clustering and analyzing high-quality fibroblasts, we identified and phenotypically classified CAFs into adhesion/wound healing/CAF-S1, perivascular/CAF-S4 subtypes in all four datasets (Fig. 3A and B, Additional file 7: Table S6) [42]. CAF-S1s were identified by the expression of fibroblast-specific markers (FAP, PDPN, PDGFRA). CAF-S1s were further divided into (a) myo-fibroblastic (myCAF) (enhanced expression of collagen-related genes (COL1A1 and COL1A2) and fibroblast markers (FAP, PDPN) and (b) inflammatory (iCAF) subtypes (express chemokines such as CXCL12) (Additional file 1: Fig. S11) [36].

A recent study in breast cancer (BC) improved the resolution of CAF-S1 (myCAF, iCAF) by showing that CAF-S1 can be further subdivided into at least five subtypes [28]. To determine whether any of these five subtypes exist in CRC, we compared CRC CAF-S1 cells with those in the BC cohort. Using a computational pipeline, we analyzed a large dataset of 18,296 CAF-S1 cells from the BC cohort and 4685 CRC CAF-S1 cells (Fig. 3C, Additional file 1: Figs. S12–S18) [44]. By comparing differentially expressed genes between matched BC and CRC clusters [28], we identified all five distinct CAF-S1 subtypes in patients with CRC: ecm-myCAF (*GJB; ANTXR1*, and *SDC1*), wound-myCAF (*SEMA3C*; *ANTXR1* and *CD9*), TGF*ß*-myCAF (*CST1*; *TGFß1*; *ANTXR1* and *LAMP5*), IL-iCAF (*SCARA5*; *DLK1*), and detox-iCAF (*ADH1B*; *GPC3*) (Fig. 3C, Additional file 1: Figs. S12–S18). The ecm-myCAFs and TGF*ß*-myCAFs are known to be enriched in tumors with high regulatory T lymphocytes (Tregs) and depleted CD8+ lymphocytes, which are considered to correlate with immunosuppressive conditions. Paradoxically, wound-myCAFs are not linked to an immunosuppressive environment, and are associated with a high level of T lymphocyte infiltration in tumors. Lastly, all three subtypes (ecm-myCAFs, TGF*ß*-myCAFs, and wound-myCAF) are linked to primary immunotherapy resistance in melanoma and lung cancer [28].

The CAF-S4 population expressed pericyte markers (*RGS5*, *CSPG4*, and *PDGFRB*), CD248 (endosialin) and *EPAS1* (*HIF2*), implying that this CAF subtype is vessel-associated, with hypoxia likely contributing to invasion and metastasis as proposed by others (Fig. 3A-B, Additional file 7: Table S6) [36]. To validate the markers of these CAFs at the protein level, we performed immunohistochemistry (IHC) on an independent CRC cohort and identified CAF-S1 and CAF-S4 subtypes (Fig. 3D).

To examine CAFs in the context of CMS categorization, we utilized two bulk gene expression (GSE39582 [45], GSE17536 [48]) datasets and performed deconvolution using two independent methods (See Methods) [35, 36, 45, 46]. We were able to predict gene signatures encompassing all five CAF-S1 subtypes and CAF-S4 (Fig. 3E, Additional file 1: Fig. S19A and B). Deconvolution predicted significant CAFs and endothelial cell enrichment in CMS4 patients, which is consistent with tumors that are highly vascularized and inflammatory, and have high CAF content in their microenvironment [10]. CMS1 also had higher CAF enrichment than the CMS2 and CMS3 subtypes. A subset of CMS1 and CMS2 patients also had high levels of CAF infiltration, implying that CRCs are more heterogeneous than one would predict from bulk transcriptomics-based classifications. Taken together, these results indicate the relevance of CAFs to various subtypes within the CRC microenvironment.

### Tumor-associated macrophages are tumor suppressive M2 polarized cells in colorectal tumors

**Fig. 4:**
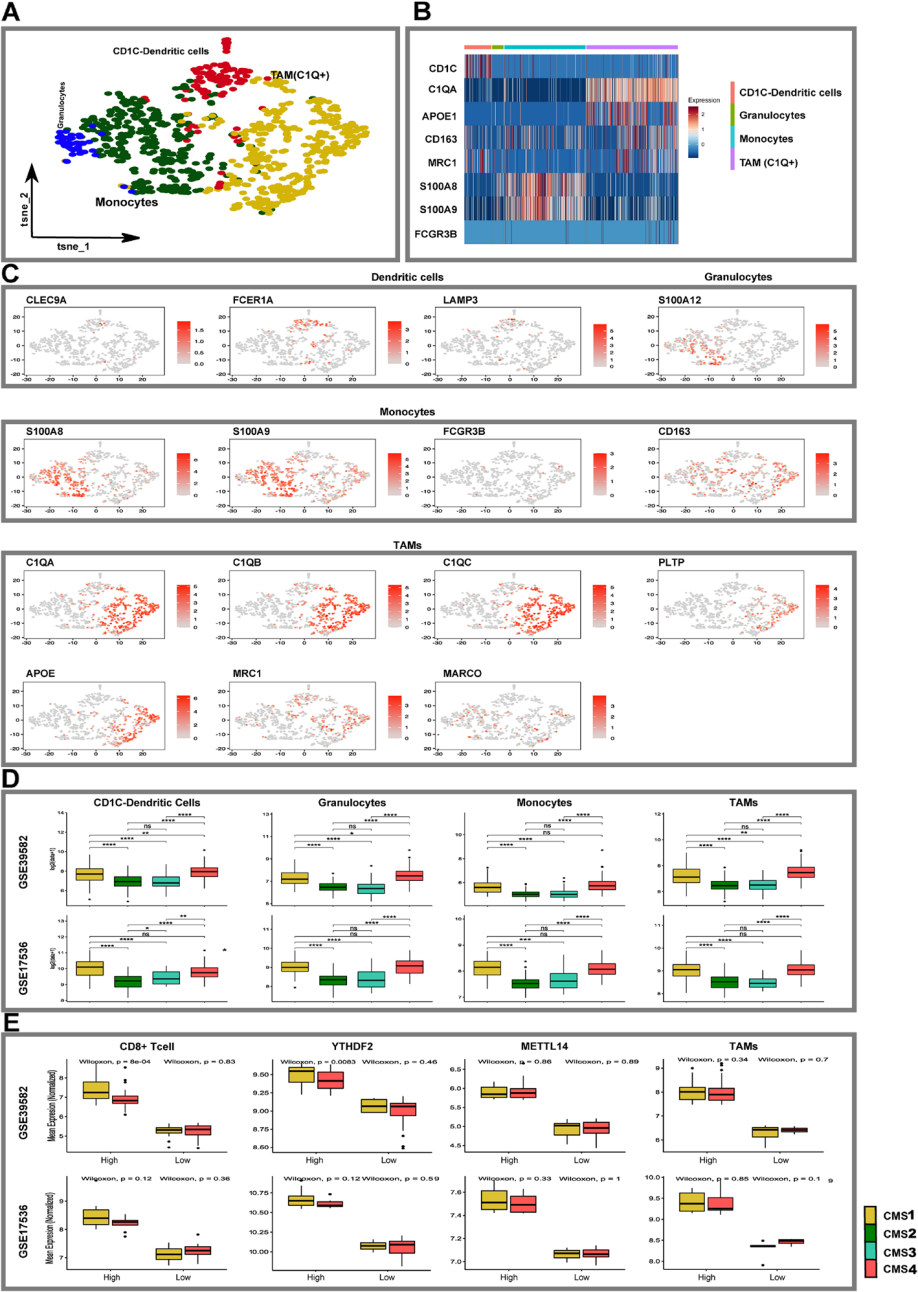
Myeloid cell clusters in colon and colorectal tumors. **A** t-SNE of myeloid cells colored. by distinct cell types. **B** Heatmap showing the variable expression of myeloid specific marker genes across various myeloid cell types. **C** Identification of various myeloid cell subtypes based on expression of specified marker genes. **D** Boxplots show the distribution of cell types in two CRC bulk expression datasets, within tumors with varying CMS status [45, 48]. The whiskers depict the 1.5 x IQR. The *P*-values for pairwise *t*-tests comparisons (with Benjamini-Hochberg correction) of cell abundance across CMS are shown in the figure. **E** Box plot show TAMs infiltration and influence on CD8+ T cells in relation to CMS1 and CMS4. Note in GSE17536 Ythdf2 deficiency trended in CMS4 but did not reach the statistical significance. Note “NS”: *P* > 0.05, *P </=0.05, **P </=0.01, ***P </=0.001, ****P </=0.0001

To examine the myeloid compartment, we re-clustered these cells and identified CD1C+ dendritic cells, tumor-associated macrophages (C1Q+ TAMs, MRC1+), monocytes (*S100A8+)* and granulocyte clusters in all four datasets (Fig. 4A-C, Additional file 1: Figs. S20–S23, Additional file 8: Table S7). Monocytes revealed proinflammatory phenotypes (1L1B, S100A8, S100A9), while TAMs showed anti-inflammatory (APOE, SEPP1, CD163) signatures [18]. TAM cells also exhibited signatures consistent with a C1Q+ phenotype which is known to be immunosuppressive (Fig. 4B) [47]. Finally, by interrogating bulk datasets, we examined whether C1Q+ TAMs were enriched in specific CMS groups (Fig. 4D, Additional file 1: Fig. S24A and B). Surprisingly, C1Q+ TAMs were enriched not only in CMS1, but also in CMS4, with no differences between CMS2 and CMS3 subtypes. Recently, C1Q+ TAMs have been reported to influence CD8+ T cell enrichment in tumors and Mettl14 or Ythdf2 deficiency in TAMs impedes tumor eradication by reducing cytotoxic T cell infiltration and encourage the accumulation of defective CD8+ T cells [47]. Interestingly, we noted lower CD8+ effector signatures in CMS4 compared to CMS1 (Fig. 4E). Further, we found lower Ythdf2 gene expression in CMS4, which could potentially account for the lower CD8+ T effector signature observed in CMS4 (Fig. 4E). In conclusion, we found C1Q+ TAMs were enriched in CMS1 and CMS4, and CD8 T effectors were reduced in CMS4, most likely related to Ythdf2 deficiency. Collectively, these findings suggest that targeting C1Q+ TAMs in CRC tumors exhibiting these signatures may enhance immunotherapies and possibly improve patient outcomes.

### Distinct states of CD8+ and CD4+ T cells shape the CRC ecosystem

**Fig. 5:**
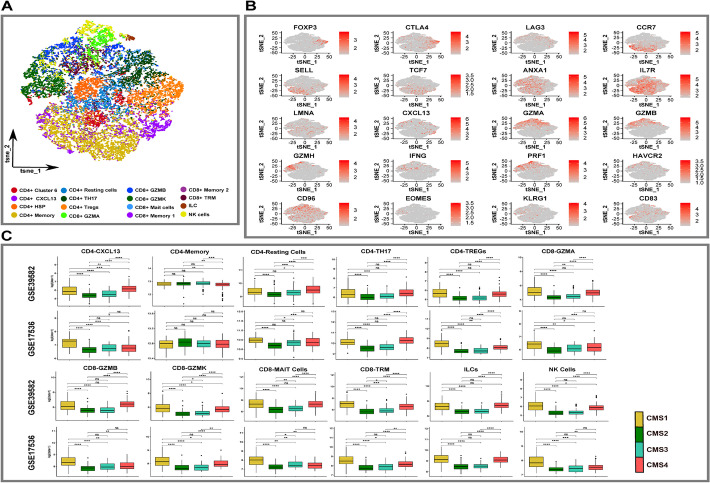
T cell clusters in colon and colorectal tumors. **A** t-SNE of 22525 T cells colored by distinct clusters. **B** t-SNE plot showing the variable expression of T cell specific marker genes across various clusters. **C** Boxplots show the distribution of cell types in two CRC balk expression datasets, within tumors with varying CMS status. The whiskers depict the 1.5 x IQR. The *P*-values for pairwise t-tests comparisons (with Benjamini-Hochberg correction) of cell abundance across CMS are shown in the figure. Note “NS”: *P* > 0.05, *P </=0.05, **P </=0.01, ***P </=0.001, ****P </=0.0001

With the exception of MSI tumors (5%), CRC patients are immune cold or lack a significant degree of immune cell infiltration, and immunotherapy in the form of immune checkpoint blockade has not improved survival in these patients [49]. Understanding how T cell diversity influences CRC TME will be crucial in designing effective treatments. Analysis of T cells identified 11 CD4+ T cell and 10 CD8+ T cell clusters, each populated by cells from multiple samples, implying shared states in CRC. Additionally, we identified natural killer (NK cells) and innate lymphoid cell (ILC) clusters (Fig. 5A and B, Additional file 1: Fig. S25A and B, Additional file 9: Table S8). Within the CD4+ T cells, we identified multiple CD4+ cell states based on gene expression markers. FOXP3 CD4+ Tregs expressing immune checkpoint markers (PD-1, LAG3, CTLA4) were among the most abundant T cells in the CRCTME compared to non-malignant tissue (Fig. 5A, Additional file 8: Table S7 and Additional file 10: S9). In addition, other CD4+ cell types were identified, including a) CD4+ memory cells expressing CCR7, SELL, TCF7; b) CD4+ resting cells expressing ANXA1, IL7R, LMNA; c) CXCL13 activated CD4+ cells (Fig. 5A, B, Additional file 9: Table S8) which have been linked to better outcomes in MSI CRC, bladder, and stomach malignancies [50–52], but were also identified in normal tissue (perhaps attributable to sequencing issues); and d) Th17 CD4+ T cells that represent critical antitumor effector cells [51].

Among the CD8+ T cell states, CD8+ GZMK, CD8+ GZMA, and CD8+ GZMB were identified as three distinct clusters of CD8+ cytotoxic cells (Fig. 5A, B, Additional files 8 and 9: Tables S7 and S8). These cell states have been identified in various tumor types [50, 53–58]. CD8+GZMK and CD8+GZMB expressed granzymes (GZMA, GZMH), interferon-gamma (IFNG), perforin (PRF1 in CD8+GZMB), and CCL4, which have been shown to mediate effector functions [26, 50–52]. However, these two cell states also demonstrated intriguing distinctions. The CD8+ GZMB population accounted for 77% of the total cells in both MSI-H CRC samples. This cell state displayed activation (CXCL13) and exhaustion (LAG3, HAVCR2, CD96) markers, which may account for their participation in the immune checkpoint inhibitor sensitivity of MSI-H colorectal cancers. CD8+GZMK cells, on the other hand, have been identified as pre-dysfunctional T cells that express pre-dysfunctional markers such as EOMES and KLRG1 [26, 50, 54]. To corroborate our findings, we examined the Pelka et al. [27] T cells (*n* = 72,209) and discovered that their T cells exhibit a similar level of cellular diversity as the primary CRC cohort (Additional file 1: Fig. S26). Additionally, CD8+ cytotoxic cells, specifically CD8+ GZMB, were shown to be overrepresented in MSI-H tumors, implying that these cell types play a role in the immune response to check point inhibitors (Additional file 1: Fig. S27).

Guided by these findings from the single-cell analysis, we interrogated bulk transcriptomic data to examine the T cell enrichment in CMS groups (Fig. 5C, Additional file 1: Fig. S28). Tumors of immune (CMS1) and mesenchymal subtypes (CMS4) exhibited strong T cell infiltration, whereas CMS2 and CMS3 had low T cell enrichment, consistent with previous data. CMS1 were predominantly enriched in NK cells and CD8+ TRM cells. Other CD4+ and CD8+ T cell types were poorly discriminative between CMS1 and CMS4 in our datasets. Overall, our analysis, based on scRNAseq and bulk data, showed that CMS1/CMS4 were immune rich whereas CMS2 and CMS3 were immune deficient. Additionally, scRNAseq revealed a CD8+ GZMB population expressing an exhaustion phenotype that was enriched in CMS1 MSI tumors, which could account for responses to immunotherapy observed in patients harboring such tumors.

### CRC patient transcriptomics distributed in a continuum, not discrete subtypes

**Fig. 6:**
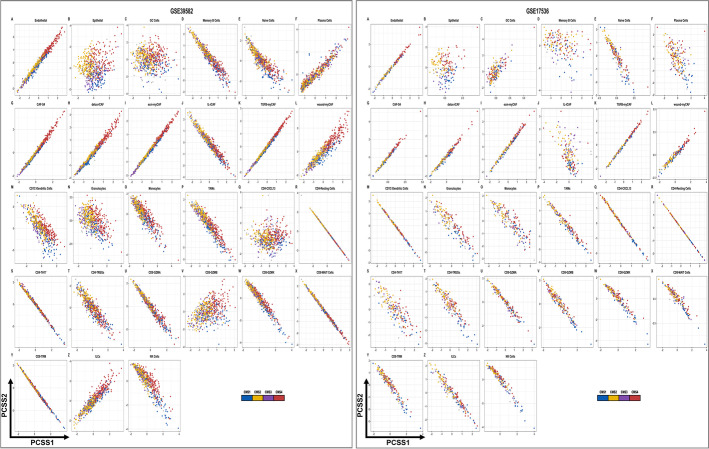
Continuous subtype scoring across cell type (GSE39582 [45], GSE1736 [48]). **A**, **B** Continuous scores reported by CMS classification across cell types show minimal separation in the top 2 principal components in GSE39582 and GSE17536 datasets respectively. All cell types are represented in CMS1-4 using PCSS1 and PCSS2 scores. Note that the cell types largely form a continuum along CMS status and are not clustered in discrete subtypes separate from one another. Cells and markers are colored by bulk CMS status accordingly to the tumor sample of origin. (PCSS1 = PC Cluster Subtype Scores1, PCSS2 = PC Cluster Subtype Scores1)

The presence of stromal and immune cells across CMS subgroups and CMS heterogeneity within tumor epithelial cells implies that CRCs are more heterogeneous than originally recognized in bulk gene expression studies. We hypothesized that CRC exists in a continuum as opposed to distinct subtypes. We used a previously continuous score model developed by a member of our research group [59]. According to this model, continuous subtype scores outperformed the distinct CMS classification scheme in characterizing clinical, biological and pathological variables that distinguish CRC tumors. Using a continuous score model, we applied single-cell signatures on two independent (*n* = 743) CRC samples (see Methods) [45, 48]. We discovered that all cell types encompassed in CRC tumors and CRC TMEs were present across the CMS groups (Fig. 6A, B, Additional file 1: Figs. S29 and S30, Additional files 10 and 11: Tables S9 and S10). Thus, CRC appears to exist in a transcriptomic continuum not only with respect to the tumor cells themselves, but also in terms of the other cell types that make up the TME. These aspects were not apparent on bulk transcriptomics analysis using continuous score model. In sum, single-cell analysis offered novel insights into CRC heterogeneity beyond previous bulk transcriptomic analyses, confirming that the CRC ecosystem does not exhibit discrete subtypes but rather is more accurately represented in a transcriptomic continuum.

### CRC outcomes were defined by CAF and macrophage enrichment across CMS subgroups

**Fig. 7:**
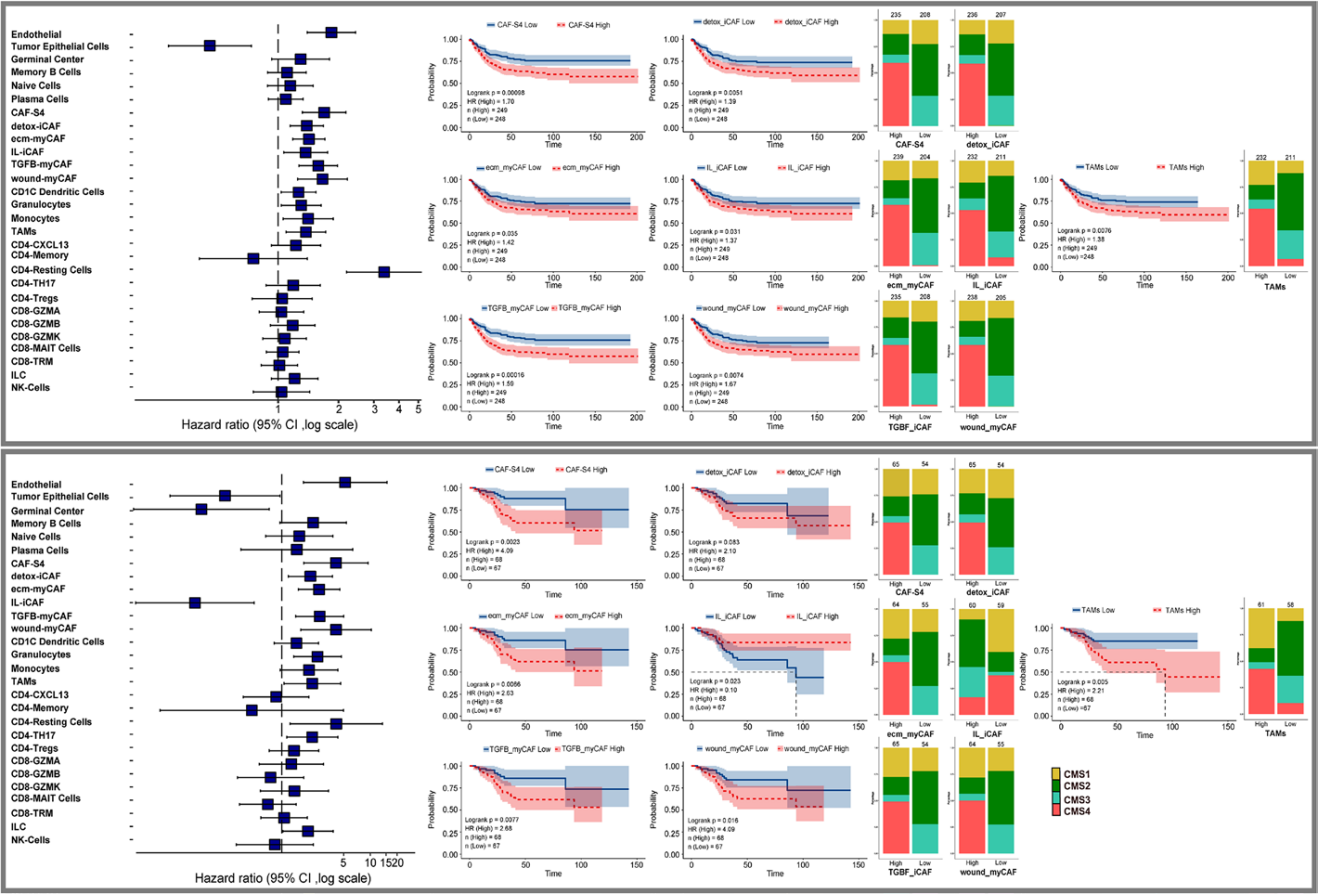
Survival analysis of two independent bulk dataset. The relationship between relative cell abundance and disease-free survival (DFS) in the GSE39582 [45] (**A**) and GSE17536 [48] (**B**) (COX regression analysis). Kaplan Meier curves depicting DFS in GSE39582 [45] (**A**) and GSE17536 [48] (**B**). Note, in addition to CMS4, CMS1-3 subgroups (good prognosis subtypes) with high CAF and C1Q+ TAMs signatures were associated with poor DFS. CAF's and C1Q+ TAMs stratified all CMS subgroups into high and low-poor survival subgroups beyond CMS categorization. HR and *P* values are indicated

Given the findings of the tumor immune stromal continuum, we next wondered which cell types impacted clinical outcomes in CRC. We analyzed two CRC patient datasets with available outcomes data and unbiasedly performed univariate Cox proportional hazard regression utilizing cell-specific expression signatures of all cellular phenotypes of CRC (Table 1) [45, 48]. CAFs, endothelium cells (EC), and C1Q+TAMs were strongly associated with poor disease-free survival (DFS) rates (Fig. 7A, B, Additional file 12: Table S11). To confirm these findings, we also performed multivariate Cox regression analysis and again demonstrated that CAFs and C1q+ TAMs were strong independent prognosticators of short DFS, adjusted by clinical features, pathological stage, chemotherapy receipt, and common mutational status (Additional file 13: Table S12) (hazard ratio for tumor recurrence (HR) > 1, *P* < 0.05).

**Table 1:** Clinical data, demographics, stage, chemotherapy, and key mutational status in GSE39582 [45] and GSE17536 [48] datasets

Characteristic	GSE39582 (***N*** = 502)	GSE17536 (***N*** = 138)
**Age at diagnosis**		
Median (IQR)	69 (59–76.9)	67 (57–75)
**Gender**		
Male	276 (45%)	71 (51%)
Female	226 (55%)	67 (49%)
**Tumor stage**		
1	33 (6.6%)	24 (17%)
2	264 (52.6%)	57 (41%)
3	205 (40.8%)	57 (41%)
**KRAS**		
M	184 (38%)	–
WT	297 (62%)	–
NA	21	–
**BRAF**		
M	45 (10%)	–
WT	403 (90%)	–
NA	54	–
**TP53**		
M	169 (53%)	–
WT	147 (47%)	–
NA	186	–
**Chemotherapy**		
Y	203 (41%)	
*N*	297 (59%)	
NA	2	
**Grade**		
WD	–	15 (11%)
MD	–	106 (77%)
PD	–	17 (12%)

*N* Number of samples, *NA* Not available, *M* Mutated, *WT* Wild type, *MD* Moderately differentiated, *PD* Poorly differentiated, *WD* Well differentiated

The CMS4 subtype demonstrated significant infiltration of CAFs and C1Q + TAMs, which is consistent with the CMS classification. We found that a subset of patient tumors in other CMS groups were also enriched with CAFs and TAMs, and such patients also had shorter DFS. Thus, CAF and TAM enrichment distinguish high-risk patients not only in CMS4, but also across the other CMS groups, further stratifying CRC patients beyond the CMS classification (Fig. 7A, B). Our findings show that CAFs and C1Q+ TAMs contributed to a significant portion of the TME in all four CMS subgroups and which are linked with poor prognosis, implying that CRC pathology can transcend CMS classification in these cases.

### CAFs and TAMs modulate immune suppression in CRC

**Fig. 8:**
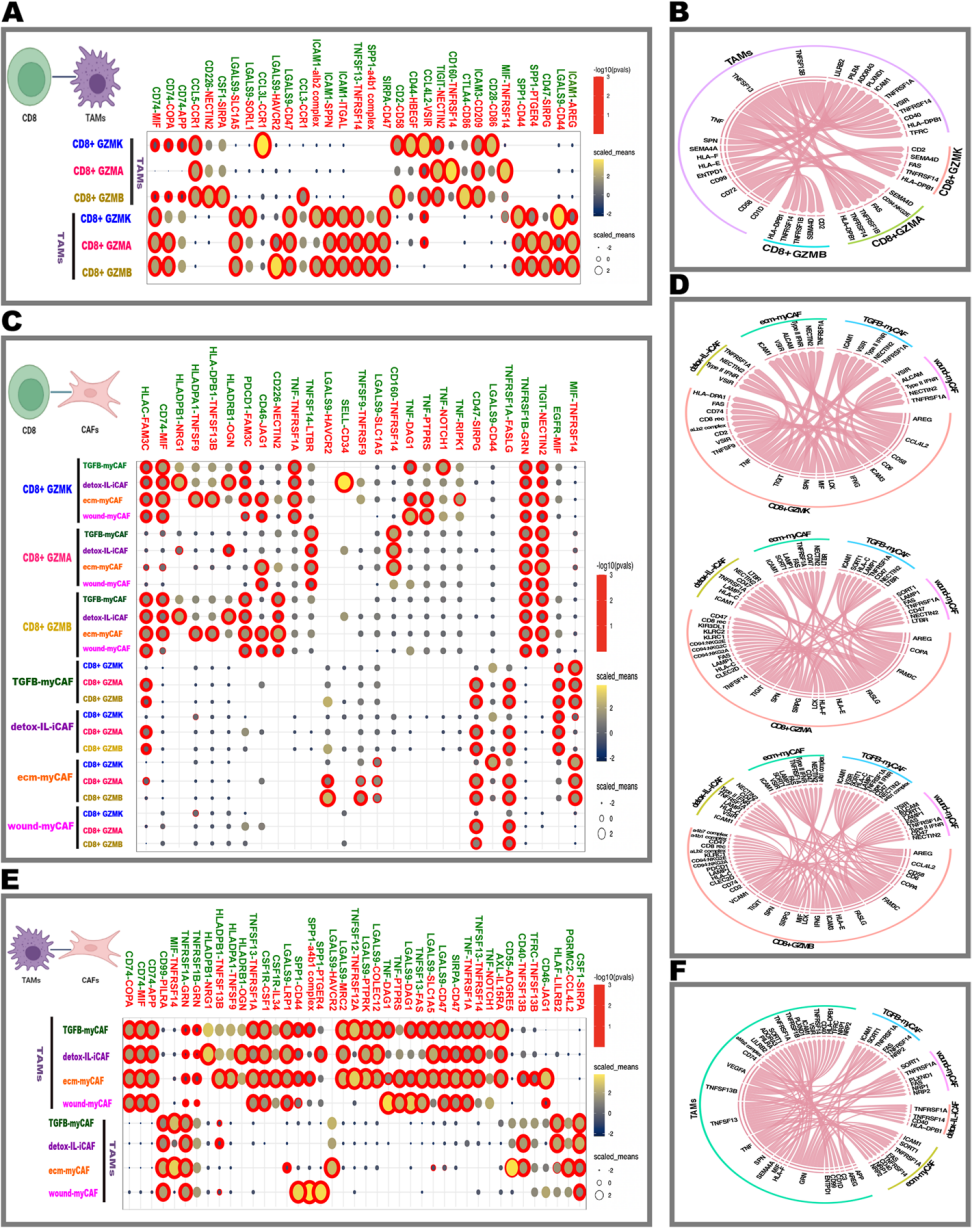
CAF, TAM and CD8+ T-cell interactions in the CRC microenviroment. **A** Dotplot showing cell-cell interaction between CD8+ T cells and TAMs. **B** Circle plot directed ligand receptor interactions in CD8+ T cells and TAMs for better visualization. **C** Dotplot showing cell-cell interaction between CD8+ T cells and CAFs. **D** Circle plot directed ligand receptor interactions in CD8+ T cells and CAFs for better visualization. **E** Dotplot demonstrating cell-cell interaction between TAMs and CAFs. **F** Circle plot directed ligand receptor interactions in TAMs and CAFs for better visualization

Given the discovery CAFs and TAMs are associated with a poor prognosis in CRC, we postulated that these cellular phenotypes govern CRC pathobiology. While single-cell analysis cannot conclusively establish cell-to-cell signaling, cell-specific receptor and ligand expression patterns can be hypothesis generating as shown in previous studies [18, 26, 32, 60]. Using cellphoneDB [61], a manually curated database of receptors, ligands, and their interactions, we found that CAF and C1q+ TAM interactions resulted in signatures that are associated with T cell dysfunction and M2-like polarization (Fig. 8A, B, Supplementary Fig. S31A-B) [60]. These ligand/receptor-mediated effects primarily involved immunological checkpoints (CD40LG:CD40, TIGIT: NECTIN2, CD74: COPA, CD28:CD86, SIRPA:CD47, and CD86:CTLA4) [60]. CTLA4-mediated trans-endocytosis of the co-stimulatory molecule CD86 from antigen-presenting cells (APCs) diminishes APCs’ ability to co-stimulate T cells [32, 62]. TAM-T cell interaction via TIGIT-NECTIN2 has been linked to immunosuppression in hepatocellular carcinoma [63]. Recent studies further demonstrate that the CD44-SPP1 signal confers resistance in glioma patients, and is associated with increased macrophage infiltration and poor overall survival [64]. CD8+ T cells expressed M2-like polarization-inducing genes (CD74:MIF, SIRPA:CD47) [60]. Our findings suggest a substantial bidirectional inhibitory crosstalk between CD8+ and C1q+TAM cells, as previously described in renal cancer, and which likely contributes to the immunosuppressive microenvironment in CRC [60].

Next, we investigated how different stromal subtypes may interact with TAMs and CD8+ T cells. We observed that both distinct and common immune checkpoint interactions exist between CAF subtypes and various CD8+ T cells (Fig. 8C, D). The TIGIT-NECTIN2 relationship, for example, was shared across CAFS1 subtypes and CD8+ T cells, but the T cell ligand- receptor inhibitory signal (LGASL9-HAVCR2) was specific to the ecm-myCAF and CD8+ GZMB pair which was abundant in our MSI-H CRC dataset. CAF-S1 and TAM interactions were mediated by CAF-S1-expressed C3 and CSF1 molecules and the TAMs receptors C3AR1, CXCR4, and SIRPA [65]. Notably, CAF-TAM interaction via MIF-CD74 increases CD44 binding in CAFs, leading to ERK1/2 activation, which promotes proliferation and inhibits apoptosis (Fig. 8E, F) [66]. Of note, others have reported that in melanoma MIF-CD74 interactions lead to myeloid-derived suppressor cell-mediated immunosuppression [67]. In addition, we found that ecm myCAFs can interact with TAMs via TGFB1-TGFBR1/2 pairings, which have been shown to promote cancer progression [68]. Taken together, our cell-cell interaction analysis suggests that CAFs and TAMs play a significant immunosuppressive role in CRC.

## Discussion

Using independent cohorts and unbiased single-cell profiling of CRC tumors and their associated microenvironment, our work provides a high-resolution depiction of cellular diversification and heterogeneity within the tumor, immune, and stromal compartments of CRC within the broader CMS context that cannot be described by bulk-level subtyping. We unearthed distinct cellular phenotypic and biological states of fibroblasts, T cells, and the myeloid compartment within the CRC CMS classification scheme. One critical finding of our work is that despite significant heterogeneity among CRC patients beyond that of the CMS classification, a dichotomy with respect to TME composition exists, with patients having higher CAF and C1Q+ TAM enrichments across the different CMS groups exhibiting relatively poorer outcomes. Thus, the status of CAFs and C1Q+ TAMs at time of diagnosis may predict clinical outcomes of CRC patients.

Our findings indicate that CRCs are intricately linked to the stroma, suggesting stromal-targeted combinatorial approaches may be a potential treatment strategy. Targeting CAFs in solid tumors has been explored in multiple clinical trials with variable results [69]. CAF heterogeneity, lack of patient stratification based on CAF signatures (thus not biomarker-driven), and their intricate interactions with the other cells in the TME are largely unaddressed in such studies. The CAF landscape in CRC remains largely unknown, and the prognostic role of stromal signatures in CRC was demonstrated using bulk transcriptomics, which lacked the resolution to clearly identify the cellular origins of CAFs [7, 11]. In the present report, we analyzed the entire ecosystem and discovered that stromal and TAM signatures, among other cellular phenotypes, predict poor outcomes in CRC.

More recently, a small study identified two CAF subtypes using only 26 single cells (17 CAF and 9 normal fibroblasts) and rely on the expression of a few marker genes, resulting in considerable classification uncertainty [36, 70]. Another recent study focused primarily on the myofibroblast component of CAFs without comparing them to established nomenclature utilized in other studies [18, 24, 28, 42, 71, 72]. We comprehensively analyzed and dissected a large number of CAF populations using comparative analysis in four independent datasets, illustrating and confirming the detailed complexity of CAF subtypes known to date and uncovering at least five CAF-S1 subtypes with clinical and therapeutic relevance. Among these, ecm-myCAF, TGFß-myCAF, and wound-myCAF subtypes are known drivers of immunosuppressive environments and immunotherapy resistance [28]. Stratifying patients based on CAF subtype signatures and targeting these subtypes may mark a critical next step in developing combinatorial immunotherapies for CRC tumors. These findings are crucial because responses to immunotherapy in microsatellite stable (MSS) CRC are lacking [4, 49], and MSS tumors account for nearly 95% of metastatic CRC. We speculate that using small molecules, biologics, or even cell-based therapies to target the ecm-myCAF, TGFß-myCAF and wound-myCAF subtypes could improve upon the current checkpoint blockade strategies against CRC [28, 56].

Tumor-associated macrophages (TAMs) promote cancer growth and metastasis while also contributing to an immune-suppressive microenvironment [73–75]. TAMs are also antagonistic to newer immunotherapies targeting PD-1/PD-1 L and the CTLA-4 axis [76–78]. We found C1Q+ M2 polarized immunosuppressive TAMs were enriched in CMS1 and CMS4. Furthermore, we observed lower Ythdf2 gene expression in the CMS4 subtype, which resulted in lower CD8+ T effector infiltration and an immunosuppressive TME within this subgroup. Conceivably, depleting C1Q+ M2 TAM cells may be therapeutically useful in CRC.

We investigated for the first time whether intrinsic characteristics of tumor cells contribute to stromal and immune infiltration in CMS subgroups at the single-cell resolution level. In spite the fact that CMS group reproducibility was demonstrated in primary CRC tumor cells, our single-cell analysis adds another level of complexity and shows that CMS subgroups shared several pathways, reflecting intra-tumoral CMS heterogeneity more consistent with a continuum than discrete subtypes. These findings contradict the findings of Lee et al. [18], who reported that tumor epithelial cells aligned along their transcriptional CMS features. However, on reanalyzing their data by applying current best practices and retaining only high-quality cellular phenotypes, we found their data aligned perfectly with our observations showing intra-tumoral CMS heterogeneity beyond that described by bulk-level CMS subtyping (Additional file 1: Figs. S2–S5). Our results illustrate and clarify why cancers with diverse clonal populations respond in unpredictable ways to monolithic treatment strategies based on bulk sequencing that “target” average expression profiles. Developing precision CRC therapeutics targeting sub-clonal transcriptome programs is likely to be more effective.

Another interesting finding of our study was the similar pathway activation profiles at the tumor epithelial level among MSI-H and MSS CRCs within the CMS1 subgroup. The differences between these tumors lie in MSI-H CRCs’ decreased DNA repair ability, resulting in the generation of tumor-related neoantigens that attract CD8+ cytotoxic T cells in their TME, thus accounting for the response of MSI-H CRC tumors to immunotherapy [27, 79, 80].

Our study has some noteworthy limitations. Despite validating the clinical relevance of CAF subtypes and C1Q+ TAMs using large bulk transcriptomics data, we believe that other cell types may also play roles in CRC biology. However, bulk transcriptomics has inherent limitations such as stromal confounding and the presence of varied cell-type mixtures. In addition, using a modest number of samples and single biopsies for each sample might have understated the heterogeneity in our results. Future studies should be designed to use a broader and larger cohort of multiregional CRC biopsies for single-cell studies, as well as spatial transcriptomics, to further the understanding of CAFs and other cellular subtypes that appear to be omnipresent in CRC.

## Conclusions

In conclusion, this work explored and addressed a fundamental question as to what drives poor CRC prognosis via CAF subtypes and M2 polarized C1Q+ TAMs. Our findings provide rationale to discover therapeutic targets against CAFs, especially ecm-myCAF, TGFß-myCAF and wound-myCAF, in combination with anti-angiogenic agents, myeloid targeted therapies and immune checkpoint inhibitors. Highlighting the importance of CRC heterogeneity, we suggest the term “tumor stromal immune transcriptomic continuum” to denote the inherent non-discrete nature of this disease process*.* We have made available this comprehensive high quality cellular and molecular ontology of CRC CAFs, which should help promote the development of novel drugs and more accurate clinical biomarkers.

We hope our observations and dataset serve as a starting point for further dissection of CRC tumor biology and stratification of patients for precision medicine.

## Methods

### Experimental model and subject details

#### Collection and processing of patient tumor samples

Patients with resectable untreated CRC who underwent curative colon resection at Rush University Medical Center (Chicago, IL, USA) were included in this Institutional Review Board (IRB)-approved study. CRC specimens from 16 patients, including nine Caucasian, six African American, and one Asian patient with corresponding 8 adjacent normal tissue samples, were processed immediately after collection at Rush University Medical Center Biorepository and sent for scRNA-seq. Thus, our scRNA-seq atlas represents a diverse patient population. The study was conducted in accordance with ethical standards and all patients provided written informed consent.

### Method details

#### Droplet based scRNA-seq - 10× library preparation and sequencing

Single-cell RNA sequencing (scRNA-seq) was performed using 10X Genomics Single Cell 5’ Platform. Tumors and normal colon samples were enzymatically dissociated (*Miltenyi*), filtered through a 70-micron cell strainer, pelleted after centrifugation at 300 x*g* and resuspended in DAPI-FACS buffer (PBS, 0.04% BSA). Samples were sorted and viable singlets were gated on the basis of scatter properties and DAPI exclusion. Approximately 3000 cells were pelleted and resuspended in PBS, and cells underwent single cell droplet-based capture on 10X Chromium instruments according to the 10X Genomics Single Cell 5′ Platform protocol. Transcriptome libraries were prepared post-fragmentation, end-repair, and A-tailing double-sided size selection, and subsequent adaptor ligation as per the manufacturer’s protocol. Illumina *NextSeq 550* was used for library sequencing and data were mapped and counted using Cellranger-v3.1.0 (*GRCh38/hg38*).

#### scRNA-seq data quality control, gene-expression quantification, dimensionality reduction, and identification of cell clusters

*Cell Ranger* was utilized to process the raw gene expression matrices per sample and all samples from multiple patients were combined in R package (v3.6.3 2020-02-29] – “*Holding the Windsock*”). Seurat package (v3.2.2) was used in this integrative multimodal analysis [21]. Genes detected in fewer than three cells and cells expressing less than 200 detected genes were filtered out and excluded from analysis. In addition, cells expressing > 25% mitochondria were removed. Cell cycle scoring was performed, (for the S phase and the G2M phase) and the predicted cell cycle phases were calculated. Doublet detection and any higher-order multiplets that were not dissociated during sample preparation were removed via the *DoubletFinder* (v2.0.2) package using default settings [81]. Following quality control, one normal colon sample (B-cac13) was discarded due to poor data quality. Finally, 49,859 cells remained and were utilized for downstream analysis.

We adopted the general protocol described in Stuart et al. [44] to group single cells into different cell subsets. We employed the following steps: clustering the cells within each compartment (including the selection of variable genes for each dataset based on a variance stabilizing transformation [VST]), canonical correlation analysis (CCA) to remove batch effects among the samples, reduction of dimensionality, and projection of cells onto graphs [82, 83]. Principal component analysis (PCA) was carried out on the scaled data of highly variable genes [84] The first 30 principal components (PCs) were used to cluster the cells and to perform a subtype analysis by nonlinear dimensionality reduction (t-SNE) [85, 86]. We identified cell clusters under the optimal resolution by a shared nearest neighbor (SNN) modularity optimization-based clustering method. We implemented the *FindClusters* function of the Seurat package, which first calculated *k-nearest neighbors* and constructed the SNN graph. We implemented the original *Louvain algorithm* (algorithm = 1) for modularity optimization. Additionally, we utilized Clustree (v0.4.3) and manual review for identifying the best clustering resolution [87].

#### Major cell type detection and data visualization

To identify all major cell types, we evaluated differentially expressed markers in each identity cell group by comparing them to other clusters using the Seurat *FindAllMarkers* function. We used positively expressed genes with an average expression of >/= 2-fold higher in that subcluster than the average expression in the rest of the other subclusters. We utilized known marker genes, which have the highest fold expression in that cluster with respect to the other clusters. Additionally, we utilized SingleR (v0.99.10, R Package) and Bioturing, which leverage large transcriptomic datasets of well-annotated cell types and manual annotation for cell-type identification [25, 53, 88, 89]. Depending on the presence of known marker genes the clusters were grouped as: epithelial cells (*EPCAM*, *KRT8*, and *KRT18*), fibroblasts (*COL1A1*, *DCN*, *COL1A2*, and *C1R*), endothelial cells (CD31+), myeloid cells (*LYZ*, *MARCO*, *CD68*, and *FCGR3A*), CD4 T cells (*CD4*), CD8 T cells (*CD8A* and *CD8B*), and B cells (*MZB1*) [25, 36, 89–94]. The cells were eventually assembled into DGE matrices within each compartment, containing all six cell types.

#### Major-cell type subclustering and data visualization

Each major cell type, including epithelial cells, endothelial cells, T cells, B cells, myeloid cells, and fibroblasts, was reclustered and reanalyzed to study each compartment at a higher resolution to detect granular cellular heterogeneity in CRC. Clustree (v0.4.3) and manual review were utilized for optimal cluster detection. For cell annotation of each cell type, we utilized published literature gene expression signatures and manual review of differential genes among clusters [25, 26, 47, 51, 58, 95]. Additionally, we again utilized SingleR (v0.99.10) and BioTuring for unbiased cell annotation. We utilized t-SNE for visualization purposes. The cells expressing hybrid markers were removed for downstream analysis. For validation, we analyzed 65,362 cells from 23 patients and applied the similar quality control metrics as outlined above, retaining 31,383 high-quality single cells for further analysis [96]. For validation, we analyzed additional datasets of 15,964 cells from Zhou et al. [19], 370,115 cells from Pelka et al. [27], and report 119,554 cells comprising of TME from this data in our study. We also analyzed 2212 tumor epithelial cells  from the Belgian cohort of Lee et al. for trajectory analysis [18] (see Trajectory analysis methods below) (Additional file 1: Figs. S1–S7).

The InferCNV (v1.2.1) package was used with default parameters to identify somatic large-scale chromosomal copy number alterations in epithelial cells (*EPCAM+*, *KRT8+*, *KRT18+*) [97]. Normal epithelial cells were used as the control group (Additional file 1: Fig. S32).

#### Trajectory analysis

We used Monocle v.2 (v2.14.0), a reverse graph embedding method to reconstruct single-cell trajectories in tumor and normal epithelium [40]. In brief, we used UMI count matrices and the *negbinomial.size*() parameter to create a *CellDataSet* object in the default setting. We grouped projected cells on t-SNE in default settings for visualization of monocle results. We defined the cumulative duration of the trajectory to show the average amount of transcriptional transition that a cell undergoes as it passes from the starting state to the end state. We also used slingshot R package which uses minimum spanning tree designed for multiple branching lineages for trajectory analysis. We performed slighshot wrapper function with the UMAP dimensionality reduction and cluster labels as in Seurat object objects to identify the trajectory in our study [41].

#### Pathway-gene set variation analysis (GSVA)

Pathway analysis was performed on the customized collection of 51 CRC-related gene sets listed in Additional file 13: Table S12. We used GSVA (v1.34.0), a non-parametric, unsupervised method to estimate the gene set variations and evaluation of pathway enrichment, and pathway scores were calculated for each cell using standard settings [98, 99].

#### Comparative analysis

Integration of the breast cancer (Kieffer et al.) and CRC dataset (Our CRC data and Zhou et al.) scRNA-seq with our dataset was carried out using standard Seurat functions [19, 28]. Datasets were normalized and variable features were identified using *FindVariableFeatures()* function. Features were selected based on their repeated variable datasets for integration and anchors were identified using *FindIntegrationAnchors*() function. The identified anchors were used to integrate the datasets together with *IntegrateData*() function. 30 principal components (PC) were used in the weighting procedure. Data were scaled using *ScaleData*() function. we Computed k.param nearest neighbors and constructed a shared nearest neighbor graph by calculating the neighborhood overlap (Jaccard index) between every cell and its k.param nearest neighbors using *FindNeighbors*() function. The *FindClusters*() function was used to identify clusters of cells using a shared nearest neighbor (SNN) modularity optimization based clustering algorithm with a resolution parameter of 0.5. For regression, the variables “nUMI” and “percent.mt” were used.

#### DNA and bulk RNA library construction

DNA and bulk RNA sequencing was performed as previously described [100]. One hundred nanograms of DNA from each tumor was mechanically sheared to an average size of 200 bp. Using the *KAPA Hyper Prep Pack*, DNA libraries were packed, hybridized into the *xT probe* package, and amplified with the *KAPA HiFi HotStart ReadyMix*. For uniformity, each sample needed to have 95% of all targeted base pairs sequenced to a minimum depth of 300x. One hundred nanograms of RNA per tumor sample was heat fragmented to a mean size of 200 base pairs in the presence of magnesium. Using random primers, the RNA was used for first-strand cDNA synthesis, followed by second-strand synthesis and A-tailing, adapter ligation, bead-based cleanup, and amplification of the library. After library planning, the *IDT xGEN Exome Test Panel* was hybridized with samples. Streptavidin-coated beads and target recovery were carried out, accompanied by amplification using the *KAPA HiFi* library amplification package. The RNA libraries were sequenced on an *Illumina HiSeq 4000* using patterned flow cell technology to achieve at least 50 million reads.

#### Detection of somatic variation on DNA sequencing data

The tumor and normal FASTQ files were paired. For quality management measurement, FASTQ files were evaluated using FASTQC and matched with Novoalign (Novocraft, Inc.) [100, 101]. SAM files were generated and converted to BAM files. The BAM files were sorted, and duplicates were marked. Single nucleotide variations (SNVs) were called after alignment and sorting. For discovery of copy number alterations, the de-duplicated BAM files and the VCF generated from the variant calling pipeline were processed to computate read depth and variance of heterozygous germline SNVs between the tumor sample and normal sample. Binary circular segmentation was introduced and segments with strongly differential log_2_ ratios between the tumor and its comparator were chosen. From a combination of differential coverage in segmented regions and estimation of stromal admixture provided by analysis of heterozygous germline SNVs, an estimated integer copy number was determined.

#### Microsatellite instability status

Probes for 43 microsatellite regions were developed using *Tempus xT* assay [100]. Tumors were categorized into three groups by the MSI classification algorithm as described by Tempus: microsatellite instability-high (MSI-H), microsatellite stable (MSS) or microsatellite equivocal (MSE). MSI screening for paired tumor-normal patients used reads mapped to the microsatellite loci with at least 5 bps flanking the microsatellite. The sample was graded as MSI-H if there was a > 70% chance of MSI-H classification. If the likelihood of MSI-H status was 30–70%, the test findings were too ambiguous to interpret and those samples were listed as MSE. If there was a < 30% chance of MSI-H status, the sample was called MSS. Additionally, IHC results were used to classify tumors into MSS or MSI molecular subtypes. Both of these modalities were concordant and produced the same results.

#### Bulk RNA-seq and microarray analysis

We downloaded gene expression datasets GSE17536 [48] and GSE39582 [45] to validate our findings from the single cell compartments by deconvoluting the bulk gene expression profiles into pseudo single-cell resolutions. We used Affy (v1.64.0) for the data analysis and for exploration of Affymetrix oligonucleotide array probe level data [102]. Batch correction was carried out using the *removeBatchEffect* (v3.42.2) function of the LIMMA program and CMScaller for the CMS classification (see below) [103]. To identify the top correlated marker genes for each cell types (all subtypes of B cells, Endothelial, Epithelial, Fibroblast, Myeloid, and T cells) in the bulk gene data sets, the marker genes with an average log2 FC > = 0.5 and adjusted *P* < 0.05 obtained from the SC analysis of each cell type were separately intersected with the bulk gene expression sets individually. Genes that have an average Spearman correlation score greater than 0.5 with others were kept as the cell signatures of the corresponding cell type within the bulk gene expression. Afterwards, we removed the highly correlated genes from the cell signature gene lists, if they exist in more than one cell type to make the lists mutually exclusive. Thereby, we obtained the marker gene list that is unique for each cell type.

We also used CIBERSORTx v1 to estimate composition of various cell populations in GSE39582 [45] and GSE17536 [48]. Signature gene matrices were created using the expression profiles of cells as the reference single cell profile. We ran the “hires” module with default parameters except for the “rmbatchBmode,” and the bulk-mode batch correction argument was set to true. After the deconvolution process, we normalized the gene expressions according to the cell fractions in each sample and calculated each gene’s *Z*-transformed expression values. The average normalized expression of each cell type across all samples was plotted with the heatmap.3 R function of the GMD package (v0.3.3) [19]. A signature matrix highlighting marker genes of the different cell types was prepared with a heatmap.2 R function of ggplot (v3.1.1).

#### Pseudo-bulk differential expression

We used a pseudo-bulk approach to perform differential gene expression (DGE) analysis. We used DESeq2 to normalize the count data to account for differences in library sizes and RNA composition between CMS types [104]. The normalized counts were used for QC at the gene and CMS level. Normalization and log2-transformed counts were used for unsupervised distances/clustering. We have used median of ratios method for count normalization and a regularized log transform (*rlog*) of the normalized counts for CMS-level QC as it moderates the variance across the mean, improving the clustering. DESeq2 was used to model the raw counts, using normalization factors (size factors) to account for differences in library depth. Then, we have estimated the gene-wise dispersions to generate more accurate estimates of dispersion to model the counts to fit the negative binomial model and perform hypothesis testing using the Wald test.

#### Batch-effect correction for malignant cells

We merged tumor cells from the Lee et al. [18]. (*n* = 23) and our CRC (*n* = 15) samples (*N* = 7,530 cells) and used *canonical correlation analysis* (CCA) to perform batch correction [96]. We reduced the dimensionality of the data and captured the most correlated data features, which allowed us to align the data batches. The cell mappings across datasets were then found and the data was reconstructed in a shared space using the *mutual closest neighbors* (MNNs) approach [105]. The Pseudobulk expression counts were calculated using the adjusted anchors from the pooled data. In addition, we used the *Limma* function *removeBatchEffect* [103] to remove the batch effect from the Pseudobulk expression counts (Additional file 1: Supplementary Fig. S35).

#### Consensus molecular subtyping of colorectal cancer (CMS classification)

We used R package CMScaller (v0.9.2), a nearest template prediction (NTP) algorithm, for the classification of gene expression datasets [106]. We set the permutation number to 1000 to predict the CMS classes of the samples in the GEO datasets with a *P*-value < 0.05. We ran CMScaller with default parameters.

#### Dissecting the cell-cell communications using CellPhoneDB

For cell-to-cell interaction study, we retrieved ligand and receptor information from the CellPhoneDB repository (https://www.cellphonedb.org) [61]. Among the ligands and receptors discovered in our datasets, we only kept the ones that were expressed at least in 10% of the cells from each patient. We performed a permutation test for each cell at 1000 times to compute the significance of each pair. The threshold for screening was *P* value 0.05. *P*-values and log2 mean expression was calculated and expressed on dotplots using ktplots [107]. Adapted from “Single-Cell Sequencing” and “Icon Pack - Tumor Types(Colon cancer)”, by BioRender.com. Retrieved from https://app.biorender.com/biorender-templates. CAF, TAMs and CD8+ T-cell  images (Icon Pack - Immunology) in Fig. 8 were downloaded from BioRender.com

#### Statistics and reproducibility

All statistical analyses and graphs were created in R (v3.6.3) and using a Python-based computational analysis tool. Schematic representations were made using the Inkscape (https://inkscape.org/) software. Dim plots, bar plots and box plots were generated using the dittoSeq (v1.1.7) package with default parameters [108]. Violin plots were generated using the patchwork (v1.1.0) package and ggplot2 (v3.3.2) package in R with default parameters. Heatmaps were generated using Morpheus.R with default parameters [109]. To compare the normalized expression profiles of the marker genes across the CMS classes, ANOVA, and the pair-wised *t*-tests were performed in R using ggpubr R (v0.4.0) package [110]. The box plots were generated using boxplot function in R base package with default parameters. The mean value of the normalized expression levels of the samples in each CMS group was demonstrated with a horizontal straight line within each box. Length of a boxplot corresponds to the interquartile range (IQR), which is defined as the range between the first and third quartiles (Q1 and Q3), whereas the whiskers are the upper and lower extreme values of the data (either data's extremum values, or the Q3+1.5*IQR and Q1–1.5*IQR values, whichever is less extreme) [111].

#### Immunohistochemistry

A board-certified GI Pathologist selected the CRC cases. IHC analysis was carried out in accordance with our previously reported methods [111, 112]. The immunostained tissue parts were examined using an EpiNIKON microscope, and images were taken using an attached camera. GI Pathologist reviewed final images for confirming fibroblast staining. The following antibodies were used to detect specific proteins: FAP Monoclonal Antibody(F11-24), eBioscience Cat# BMS 169, PDGFRA-ß: SC-19995, RGS5(B-4): SC-514184, CD146(MCAM) Mouse Monoclonal Antibody [Clone ID: UMAB154] CAT#UM800051CF.

#### Survival analysis

Survival curves were obtained by using the Kaplan-Meier method from the R package survfit (v3.2-7). The differences between the survival distributions were assessed by Log-rank test. The patients were divided into two groups (high and low) according to the median expression values of the corresponding marker gene(s) (survminer (v0.4.8)).

The proportional hazard assumption was tested to examine the fit of the model for the survival of the samples in two GEO datasets GSE17536 [48] and GSE39582 [45] with respect to the normalized mRNA expression levels. The relationship between normalized mRNA expression values of each cell and disease-free survival outcome in GSE17536 dataset was assessed using multivariable Cox proportional hazard regression adjusted by age, stage, race, gender, and grade. The relationship between normalized mRNA expression values of each cell and disease-free survival outcome in GSE39582 dataset was assessed using multivariable Cox proportional hazard regression adjusted by age, stage, gender, BRAF mutation status, KRAS mutation status, and TP53 mutation status. Statistical significance level was set to be 0.05 and *P*-values were two-sided. The multivariable Cox proportional hazard regression was performed in R (4.0.5) using “survival” package.

## Supplementary Information

**Additional file 1.** Supplementary Figures S1–S35.

**Additional file 2: Table S1.** Patient information, CMS classification and mutational status.

**Additional file 3: Table S2.** Cell count by cell type and patient from stromal, immune and Epithelial compartments.

**Additional file 4: Table S3.** Cell counts by cell subtypes and samples from stromal and immune and epithelial compartments.

**Additional file 5: Table S4.** Cell count by CMS subtype and patient from Epithelial compartments of Lee et al. 2020 [18] data and Our CRC data.

**Additional file 6: Table S5.** List of significant Pseudo Bulk differentially expressed genes in CMS from malignant cells.

**Additional file 7: Table S6.** List of significant differentially expressed genes from Fibroblast.

**Additional file 8: Table S7.** The *P*-values for pairwise t-tests comparisons (with Benjamani-Hochberg correction) of cell abundance across CMS.

**Additional file 9: Table S8.** List of significant differentially expressed genes from Myeloid cells.

**Additional file 10: Table S9.** List of significant differentially expressed genes from T-cells.

**Additional file 11: Table S10.** Continuous subtype scoring across cell type (GSE39582 [45], GSE17536 [48]).

**Additional file 12: Table S11.** List of proportional hazard assumption ratio for survival from the samples in GSE17536 [45] and GSE39582 [48.

**Additional file 13: Table S12.** Multivariate-Cox regression analysis.

**Additional file 14: Table S13.** Pathway analysis on the customized collection of 51 CRC-related gene sets.

**Additional file 15.** Review history.

## Data Availability

Processed scRNA-seq and metadata are available in the NCBI Gene Expression Omnibus (GEO) database under the accession code GSE200997 [113]. Additionally, Seurat objects, matrix files are available on GitHub [43]. It is also been deposited to Zenodo (https://zenodo.org/) with assigned DOI: 10.5281/zenodo.6466249 [114]. Public datasets used in our analysis were downloaded from GEO under accession numbers GSE39582 [45], GSE17536 [48], GSE132465 [18], GSE144735 [18], and GSE178341 [19]; raw counts were directly obtained from the author [19], and scRNA-seq data from Kieffer et al. [28] was downloaded from Bioturing platform [21]. Due to privacy concerns for human patients, the raw FASTQ data used in this study will be made available upon request for scientific research.
