# Correction: Refining colorectal cancer classification and clinical stratification through a single-cell atlas

**DOI:** 10.1186/s13059-022-02724-9

**Published:** 2022-07-13

**Authors:** Ateeq M. Khaliq, Cihat Erdogan, Zeyneb Kurt, Sultan Sevgi Turgut, Miles W. Grunvald, Tim Rand, Sonal Khare, Jefrey A. Borgia, Dana M. Hayden, Sam G. Pappas, Henry R. Govekar, Audrey E. Kam, Jochen Reiser, Kiran Turaga, Milan Radovich, Yong Zang, Yingjie Qiu, Yunlong Liu, Melissa L. Fishel, Anita Turk, Vineet Gupta, Ram Al-Sabti, Janakiraman Subramanian, Timothy M. Kuzel, Anguraj Sadanandam, Levi Waldron, Arif Hussain, Mohammad Saleem, Bassel El-Rayes, Ameen A. Salahudeen, Ashiq Masood

**Affiliations:** 1 Indiana University School of Medicine, Indianapolis, IN USA; 2 Isparta University of Applied Sciences, Isparta, Turkey; 3 Northumbria University, Upon Tyne, Newcastle, UK; 4 Yildiz Technical University, Istanbul, Turkey; 5 Rush University Medical Center, Chicago, IL USA; 6 Tempus Labs, Inc., Chicago, IL USA; 7 The University of Chicago, Chicago, IL USA; 8 Inova Schar Cancer Institute, Fairfax, VA USA; 9 Institute of Cancer Research, London, UK; 10 CUNY Graduate School of Public Health and Health Policy, New York, NY USA; 11 University of Maryland Marlene and Stewart Greenebaum Comprehensive Cancer Center, Baltimore, MD USA; 12 University of Alabama, O’Neil Comprehensive Cancer Institute, Birmingham, AL USA

**Correction: Genome Biol 23, 113 (2022)**

**https://doi.org/10.1186/s13059-022-02677-z**

Following publication of the original article [1], the authors noticed an error in Additional file 1. The incorrect Figure S5 was published.

The corrected Additional file 1 is published in the correction and the original article [1] has been updated.

## Supplementary Information

**Additional file 1:** Supplementary Figures S1–S35.
