# Supplementary material for: Refining colorectal cancer classification and clinical stratification through a single-cell atlas
Source: Genome Biol. 2022 May 11;23:113. doi: 10.1186/s13059-022-02677-z (PMC9092724; doi:10.1186/s13059-022-02677-z)
Supplement: Supplementary file 1 — Additional file 1. Supplementary Figures S1–S35. [file 13059_2022_2677_MOESM1_ESM.pdf]

Refining colorectal cancer classification and clinical stratification through a single-cell atlas

Ateeq M. Khaliq[1,] Cihat Erdogan[2,] Zeyneb Kurt[3,] Sultan Sevgi Turgut[4], Miles W. Grunvald[5], Tim Rand[6], Sonal Khare[6], Jeffrey A. Borgia[5], Dana M. Hayden[5], Sam G. Pappas[5], Henry R. Govekar[5], Audrey E. Kam[5], Jochen Reiser[5], Kiran Turaga[7], Milan Radovich[1], Yong Zang[1], Yingjie Qiu[1], Yunlong Liu[1], Melissa L. Fishel[1], Anita Turk[1], Vineet Gupta[5], Ram Al-Sabti[5], Janakiraman Subramanian[8], Timothy M. Kuzel[5], Anguraj Sadanandam[9] Levi Waldron[10], Arif Hussain[11], Mohammad Saleem[5], Bassel El-Rayes[12], Ameen A. Salahudeen[6], Ashiq Masood[*]

[1]Indiana University School of Medicine, Indianapolis, IN, USA

[2]Isparta University of Applied Sciences, Isparta, Turkey

[3]Northumbria University, Newcastle Upon Tyne, UK

[4]Yildiz Technical University, Istanbul, Turkey

[5]Rush University Medical Center, Chicago, IL, USA

[6]Tempus Labs, Inc., Chicago, IL, USA

[7]The University of Chicago, Chicago, IL, USA

[8] Inova Schar Cancer Institute, Fairfax, VA, USA

[9]Institute of Cancer Research, London, UK

[10]CUNY Graduate School of Public Health and Health Policy, New York, NY, USA

[11]University of Maryland Marlene and Stewart Greenebaum Comprehensive Cancer Center, Baltimore, MD, USA

[12] University of Alabama, O'Neil Comprehensive Cancer Institute, Birmingham, AL, USA

[13]Lead Contact

*Correspondence: asmasood@iu.edu

SUPPLEMENTARY INFORMATION: FIGURES

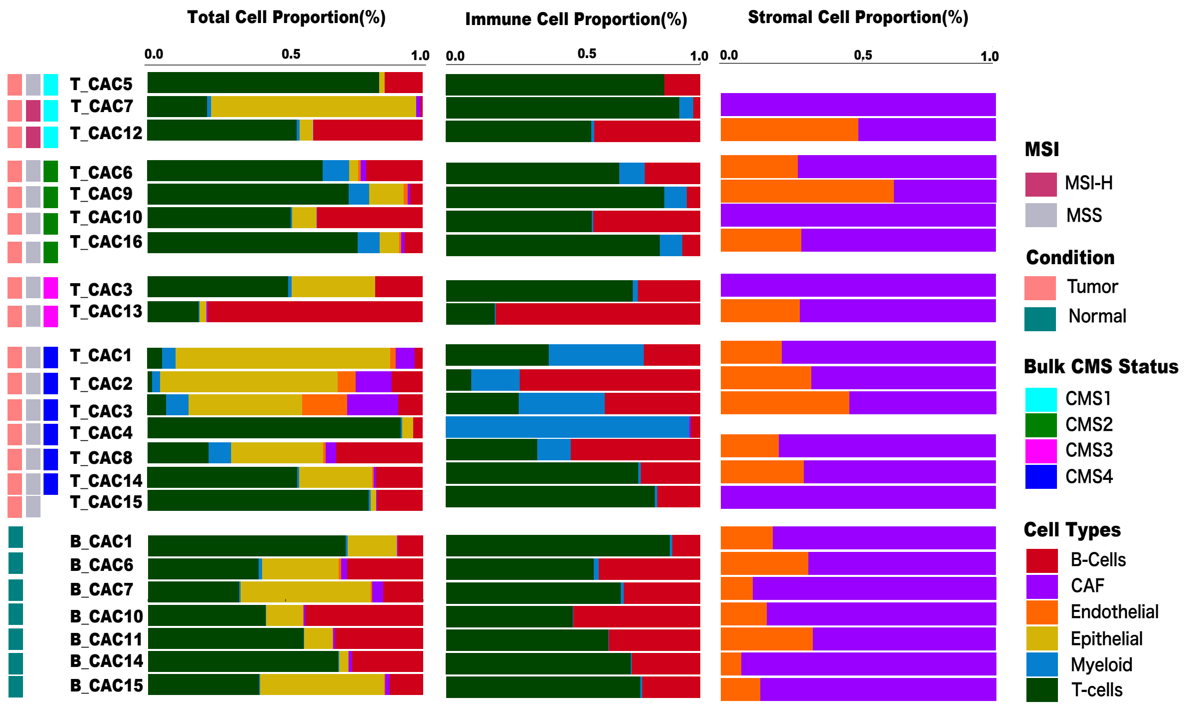

Fig. S1. Identification and clustering of single cells in primary CRC cohort. Immune and Stromal cell type proportions relative to the total cell count per patient. Each stacked bar represents a patient for which the total immune and stromal cell count proportions is scaled.

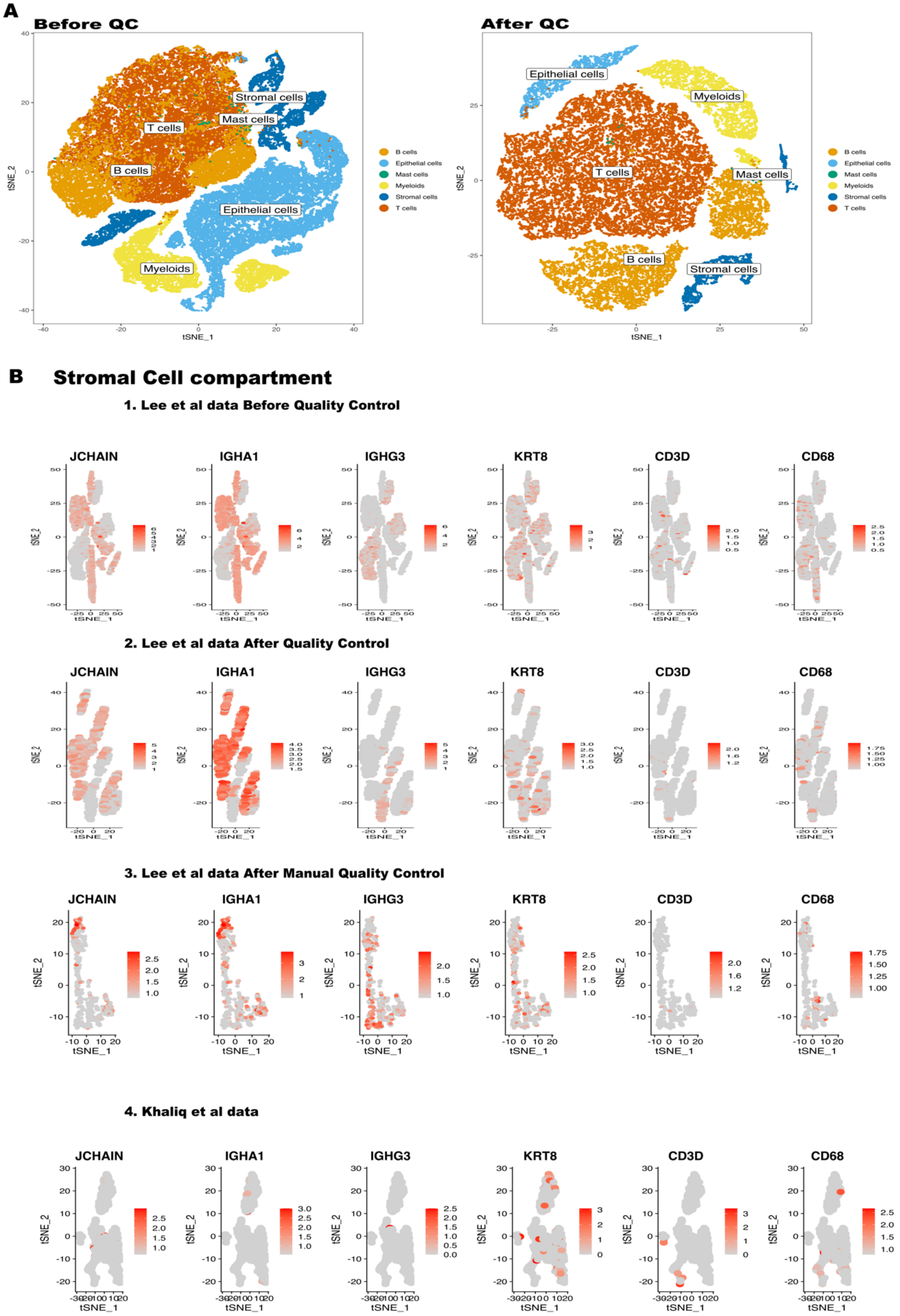
A
Before QC
After QC
T cells
B cells
Stromal cells
Mast cells
Epithelial cells
Myeloids
tSNE_1
tSNE_2
B
Stromal Cell compartment
1. Lee et al data Before Quality Control
2. Lee et al data After Quality Control
3. Lee et al data After Manual Quality Control
4. Khaliq et al data
JCHAIN
IGHA1
IGHG3
KRT8
CD3D
CD68

Fig. S2 Quality control and retained high-quality cellular phenotypes using current best practices. A, t-SNE plots showing before and after Quality Control (QC) and cell type identification. We downloaded 63689 cells from GSE132465 after quality control we only found high quality 31383 after applying the current standard practice of single cell analysis. On reclustering each cellular compartment, we noted hybrid markers in datasets that were manually removed **B, 1**. t-SNE plots showing stromal cell contamination based on the expression of specified non-stromal cell markers before QC metrics. **2**. t-SNE plots showing persistence of stromal cell contamination based on the expression of specified non-stromal cell markers after QC. **3**. We manually removed cells expressing hybrid markers and only retained only high-quality cells for our analysis. **4**. Data from our current study where we applied similar metrics including manually removing cells expressing hybrid markers to retain high quality cells.

c **Myeloid Cell Compartment**

1. Lee et al data Before Quality Control

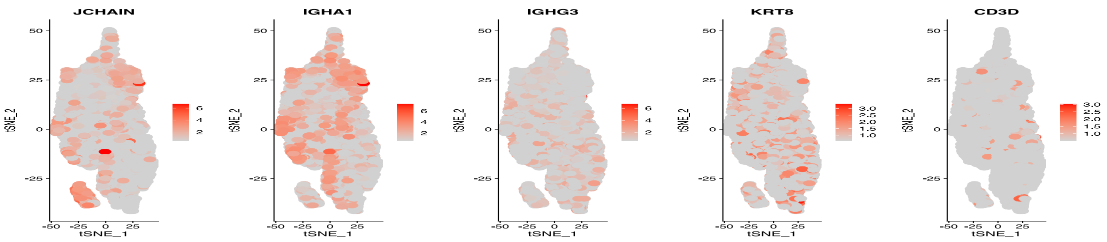

2. Lee et al data After Quality Control

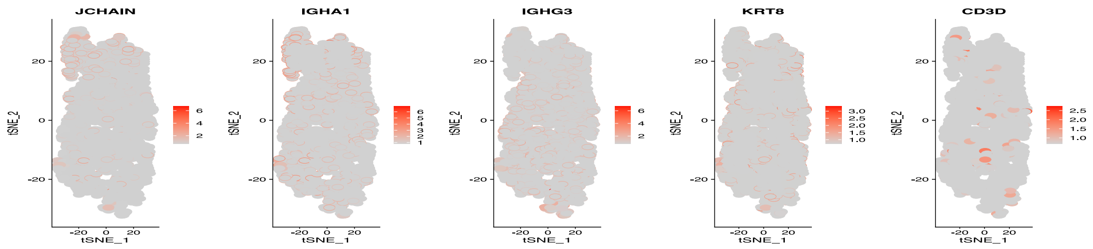

3. Lee et al data After Manual Quality Control

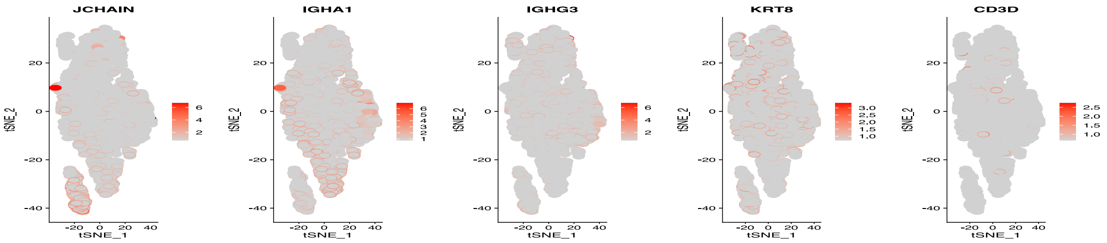

4. Khaliq et al data

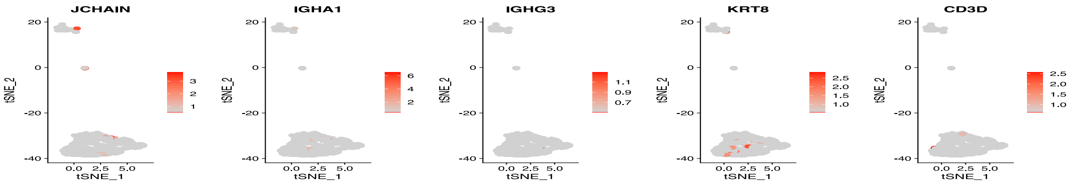

Fig. S3. Quality control and retained high-quality cellular phenotypes using current best practices. 1. t-SNE plots showing myeloid cell contamination based on the expression of specified non-myeloid cell markers before QC metrics. **2**. t-SNE plots showing persistence of myeloid cell contamination based on the expression of specified non-myeloid cell markers after QC. **3**. We manually removed cells expressing hybrid markers and only retained high quality cells. **4**. Data from our current study where we applied similar metrics including manually removing cells expressing hybrid markers to retain high quality cells.

D **T Cell compartment**

1. Lee et al data Before Quality Control

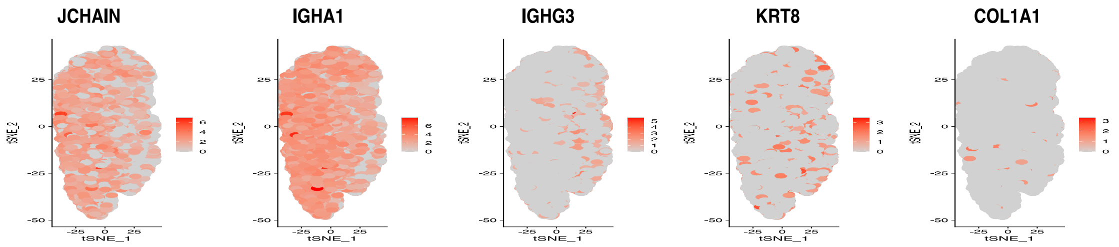

2. Lee et al data After Quality Control

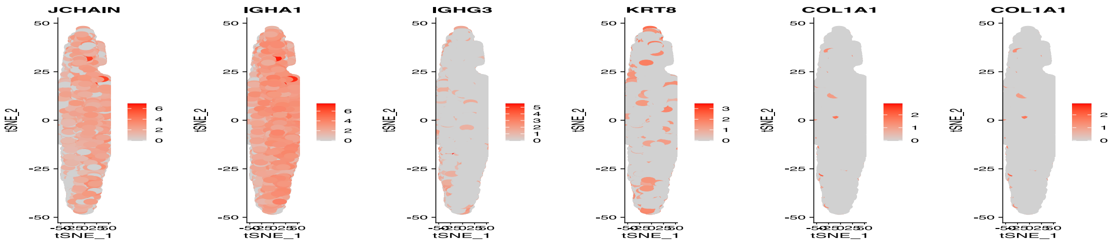

3. Lee et al data Manual After Quality Control

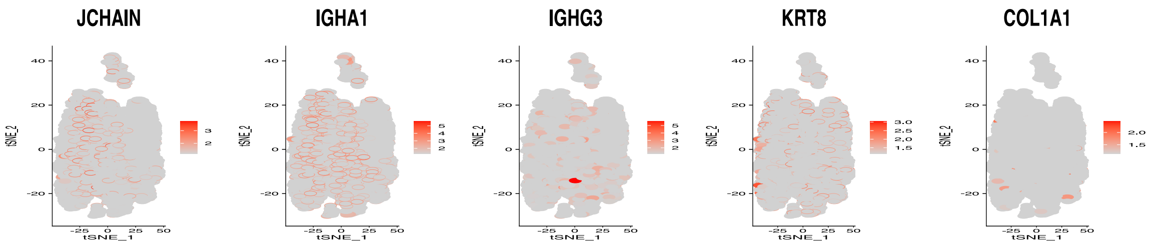

4. Khaliq et al data

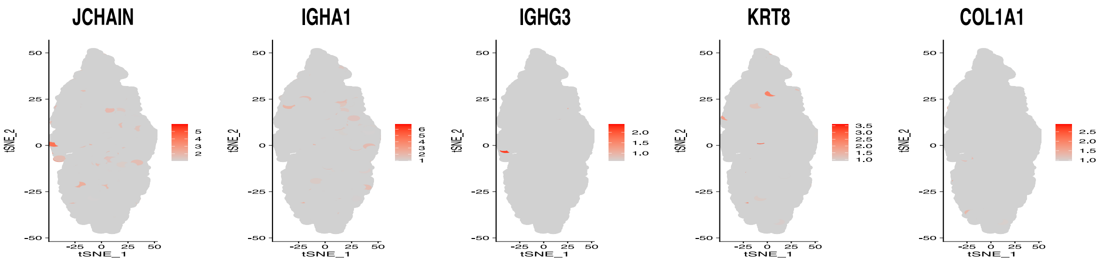

Fig. S4. Quality control and retained high-quality cellular phenotypes using current best practices. 1. t-SNE plots showing T cell contamination based on the expression of specified non-T cell markers before QC metrics. **2**. t-SNE plots showing persistence of myeloid cell contamination based on the expression of specified non-T cell markers after QC. **3**. We also manually removed cells expressing hybrid markers and only retained high quality cells. **4**. Data from our current study where we applied similar metrics including manually removing cells expressing hybrid markers to retain high quality cells.

D

T Cell compartment

1. Lee et al data Before Quality Control

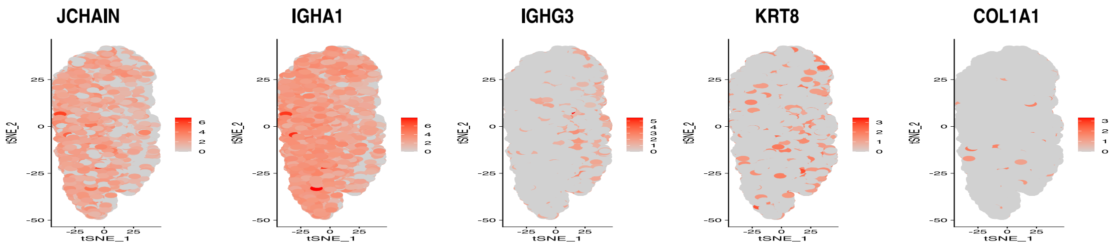

2. Lee et al data After Quality Control

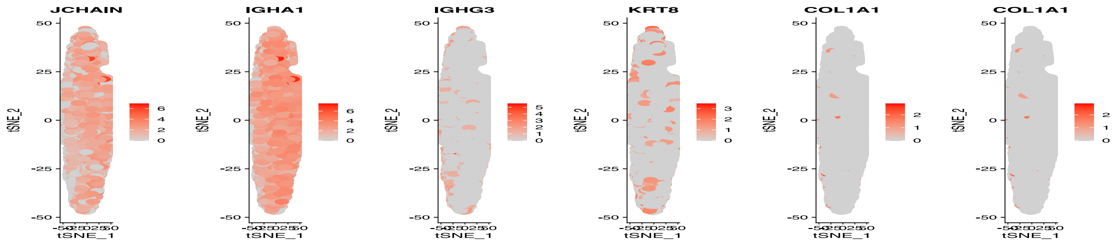

3. Lee et al data Manual After Quality Control

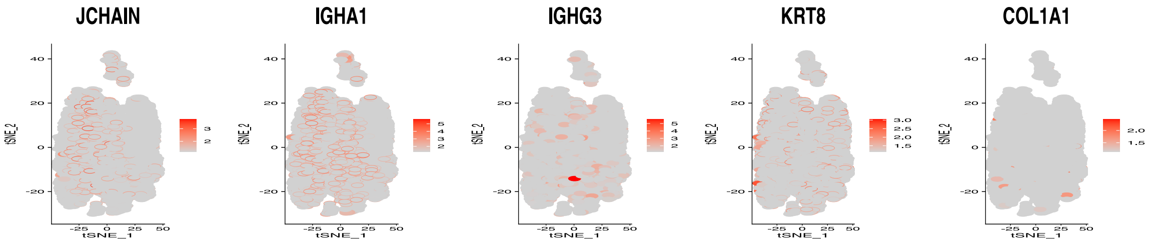

4. Khaliq et al data

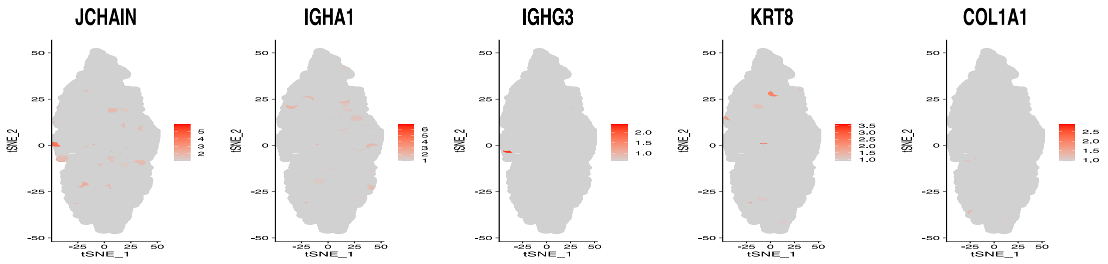

Fig. S5. Quality control and retained high-quality cellular phenotypes using current best practices. **1**. t-SNE plots showing epithelial cell contamination based on the expression of specified non-epithelial cell markers before QC metrics. **2.** t-SNE plots showing persistence of epithelial cell contamination based on the expression of specified non-epithelial cell markers after QC. **3**. We also manually removed cells expressing hybrid markers and only retained **high quality** cells. **4**. Data from our current study where we applied similar metrics including manually removing cells expressing hybrid markers to retain high quality cells.

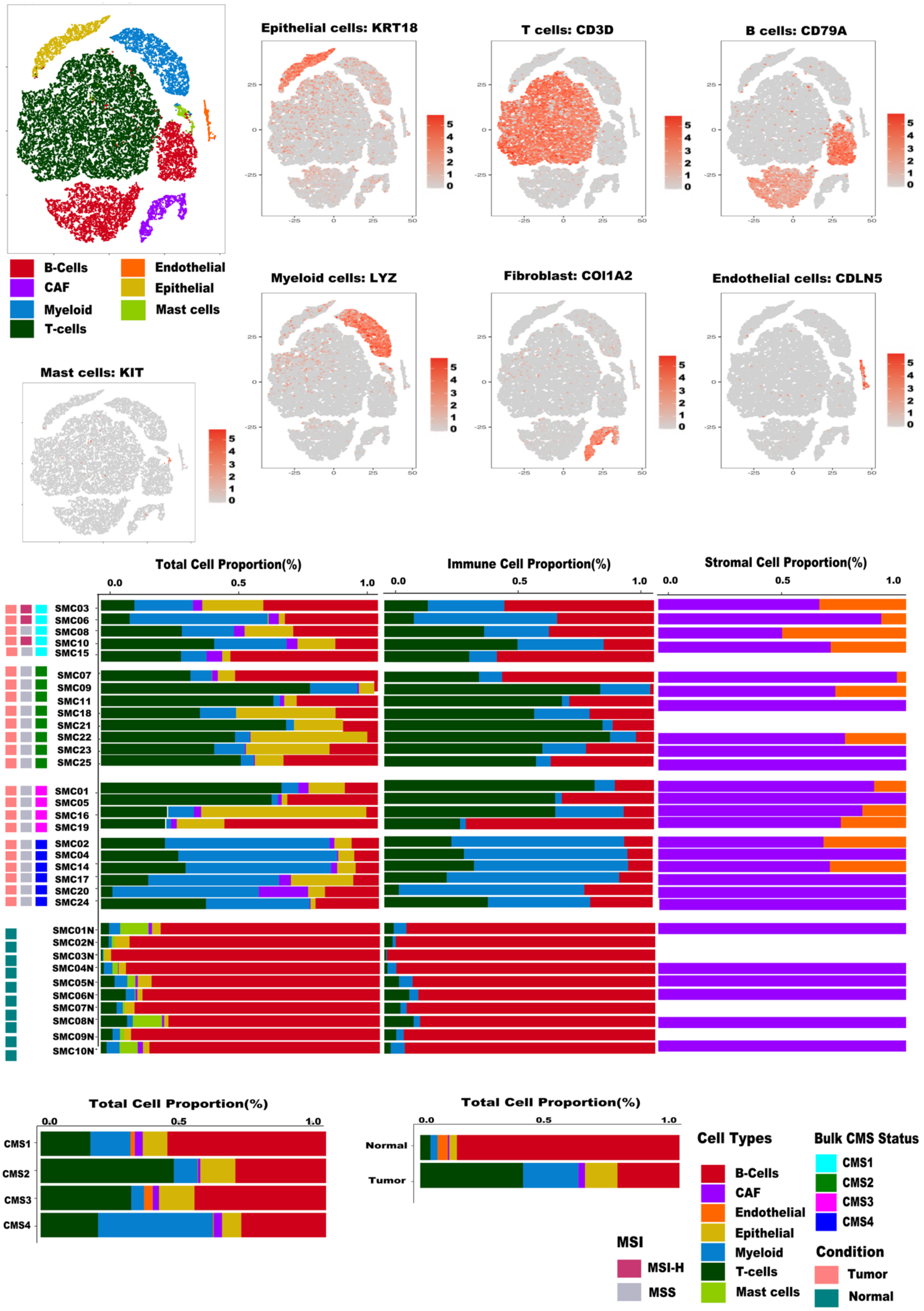

B-Cells
CAF
Myeloid
T-cells
Endothelial
Epithelial
Mast cells
Epithelial cells: KRT18
T cells: CD3D
B cells: CD79A
Myeloid cells: LYZ
Fibroblast: COl1A2
Endothelial cells: CDLN5
Mast cells: KIT
Total Cell Proportion(%)
Immune Cell Proportion(%)
Stromal Cell Proportion(%)
SMC03
SMC06
SMC08
SMC10
SMC15
SMC07
SMC09
SMC11
SMC18
SMC21
SMC22
SMC23
SMC25
SMC01
SMC05
SMC16
SMC19
SMC02
SMC04
SMC14
SMC17
SMC20
SMC24
SMC01N
SMC02N
SMC03N
SMC04N
SMC05N
SMC06N
SMC07N
SMC08N
SMC09N
SMC10N
Total Cell Proportion(%)
CMS1
CMS2
CMS3
CMS4
Total Cell Proportion(%)
Normal
Tumor
Cell Types
B-Cells
CAF
Endothelial
Epithelial
Myeloid
T-cells
Mast cells
Bulk CMS Status
CMS1
CMS2
CMS3
CMS4
MSI
MSI-H
MSS
Condition
Tumor
Normal

Fig. S6. Identification and clustering of Lee et al., 2020 single cell data. **A,** t-SNE characterization of the high-quality cells profiled. **B,** Identification of various cell types based on expression of specified marker genes. **C,** Immune and Stromal cell type proportions relative to the total cell count per patient. Each stacked bar represents a patient for which the total immune and stromal cell count proportions is scaled. **D,** Characterization of the proportion of cell types identified in each sample in tumor vs. normal colon tissue and Consensus Molecular Subtypes (CMS) of bulk RNA-seq data.

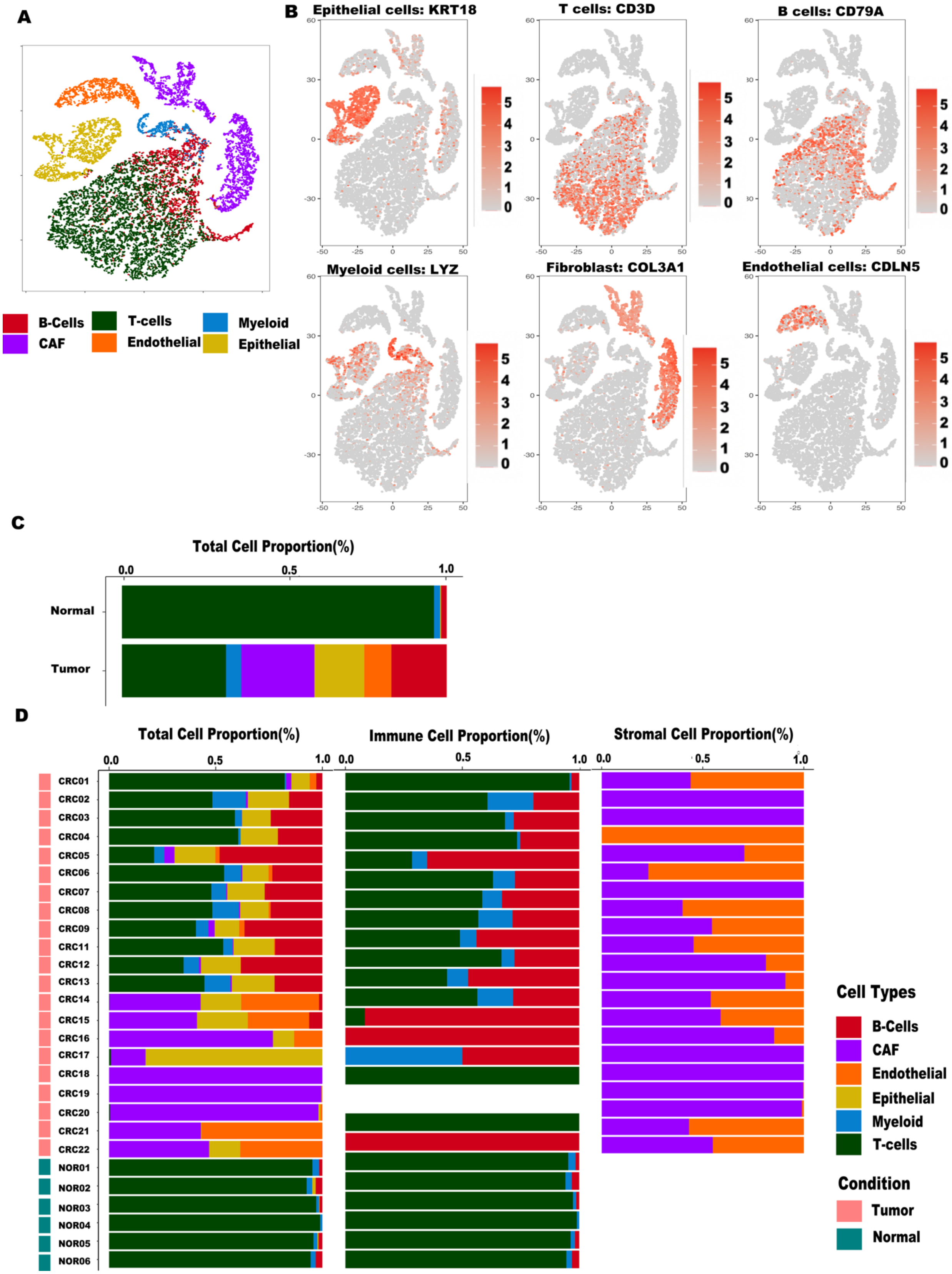
A
B-Cells
CAF
T-cells
Endothelial
Myeloid
Epithelial
B
Epithelial cells: KRT18
T cells: CD3D
B cells: CD79A
Myeloid cells: LYZ
Fibroblast: COL3A1
Endothelial cells: CDLN5
C
Total Cell Proportion(%)
Normal
Tumor
D
Total Cell Proportion(%)
Immune Cell Proportion(%)
Stromal Cell Proportion(%)
CRC01
CRC02
CRC03
CRC04
CRC05
CRC06
CRC07
CRC08
CRC09
CRC11
CRC12
CRC13
CRC14
CRC15
CRC16
CRC17
CRC18
CRC19
CRC20
CRC21
CRC22
NOR01
NOR02
NOR03
NOR04
NOR05
NOR06
Cell Types
B-Cells
CAF
Endothelial
Epithelial
Myeloid
T-cells
Condition
Tumor
Normal

Fig. S7. Identification and clustering of Zhou et al., 2020, single cell data. **A,** t-SNE characterization of the high-quality cells profiled. **B,** Identification of various cell types based on expression of specified marker genes. **C,** Characterization of the proportion of cell types identified in each sample in tumor vs. normal colon tissue and Consensus Molecular Subtypes (CMS) of bulk RNA-seq data. **D,** Immune and Stromal cell type proportions relative to the total cell count per patient. Each stacked bar represents a patient for which the total immune and stromal cell count proportions is scaled.

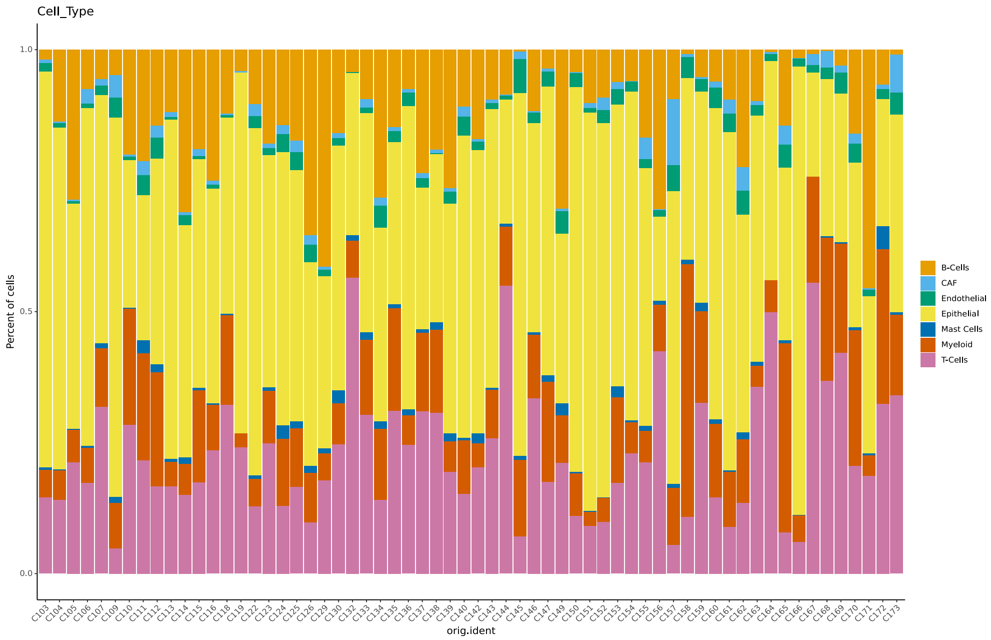

Fig. S8. Cell subtype proportions in Pelka et al., 2021 cell clusters. cell type proportions relative to the total cell count per patient. Each stacked bar represents a patient for which the total cell subtypes cell count proportions is scaled.

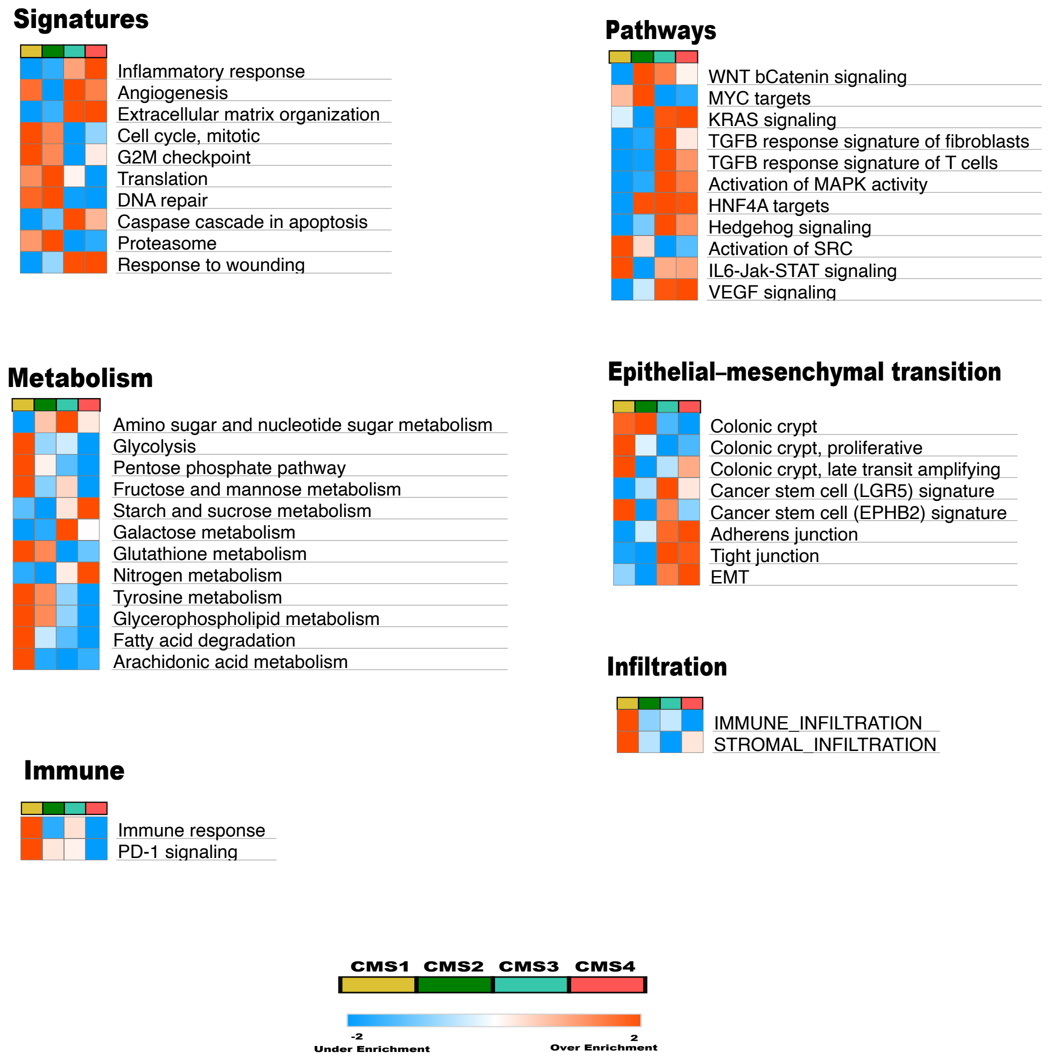

Fig. S9. Gene set enrichment of MSS tumor cells. A, gene set variation expression analyses of MSS samples, combined Lee et al., 2020 and our CRC data within the tumor epithelial cell compartment. Pseudo-bulk mRNA enrichment analysis showing gene signatures and pathway analysis.

A

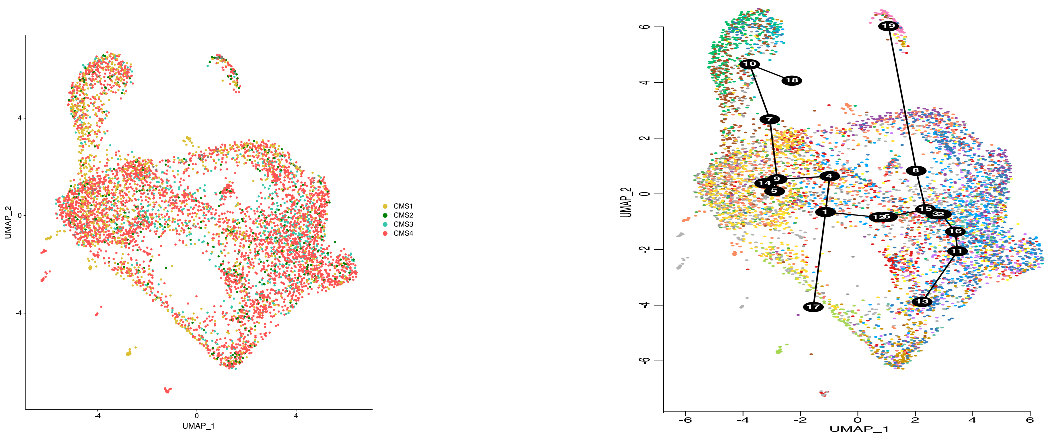
CMS1
CMS2
CMS3
CMS4
UMAP_1
UMAP_2

B

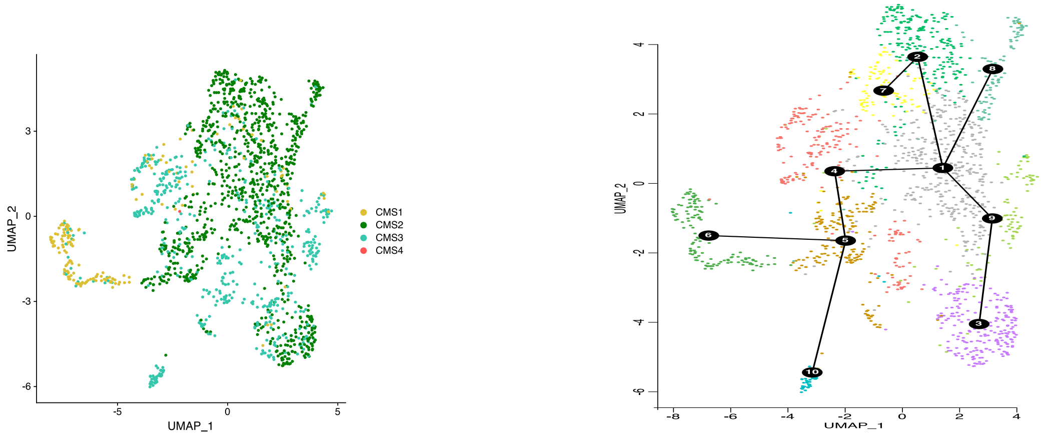
CMS1
CMS2
CMS3
CMS4
UMAP_1
UMAP_2

C

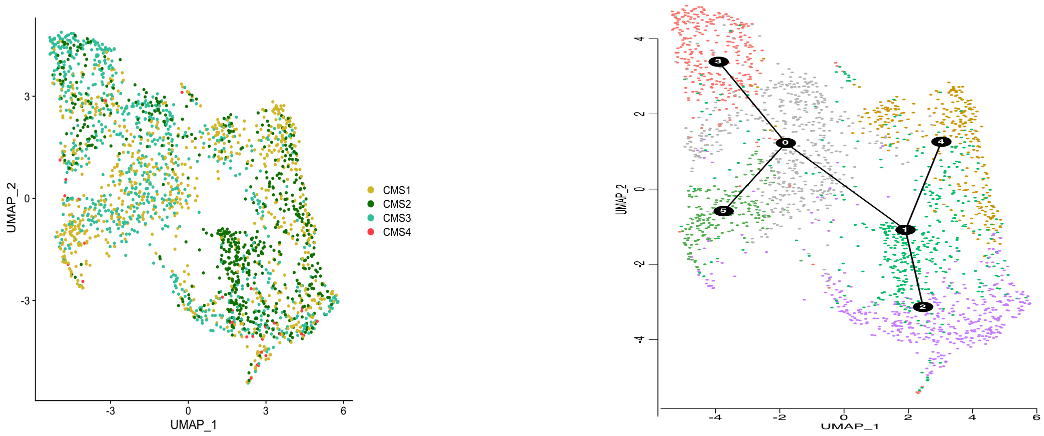
CMS1
CMS2
CMS3
CMS4
UMAP_1
UMAP_2

Fig. S10. Slingshot trajectory analysis of tumor cells. A, Trajectory analysis of primary CRC dataset colored by bulk CMS status. **B**, Trajectory analysis of Lee et al., 2020. (Korean cohart) CRC dataset colored by bulk CMS status. **C**, Trajectory analysis of Lee et al., 2020. (Belgian cohart) CRC dataset colored by bulk CMS status

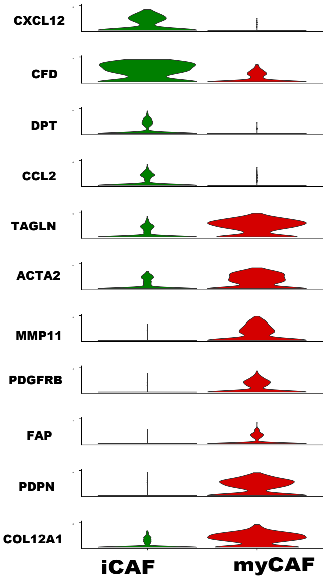

Fig. S11. Fibroblast clusters in colon and colorectal tumors. Violin plots showing the variable expression of CAF-S1 specific marker genes across myCAF and iCAF subtypes.

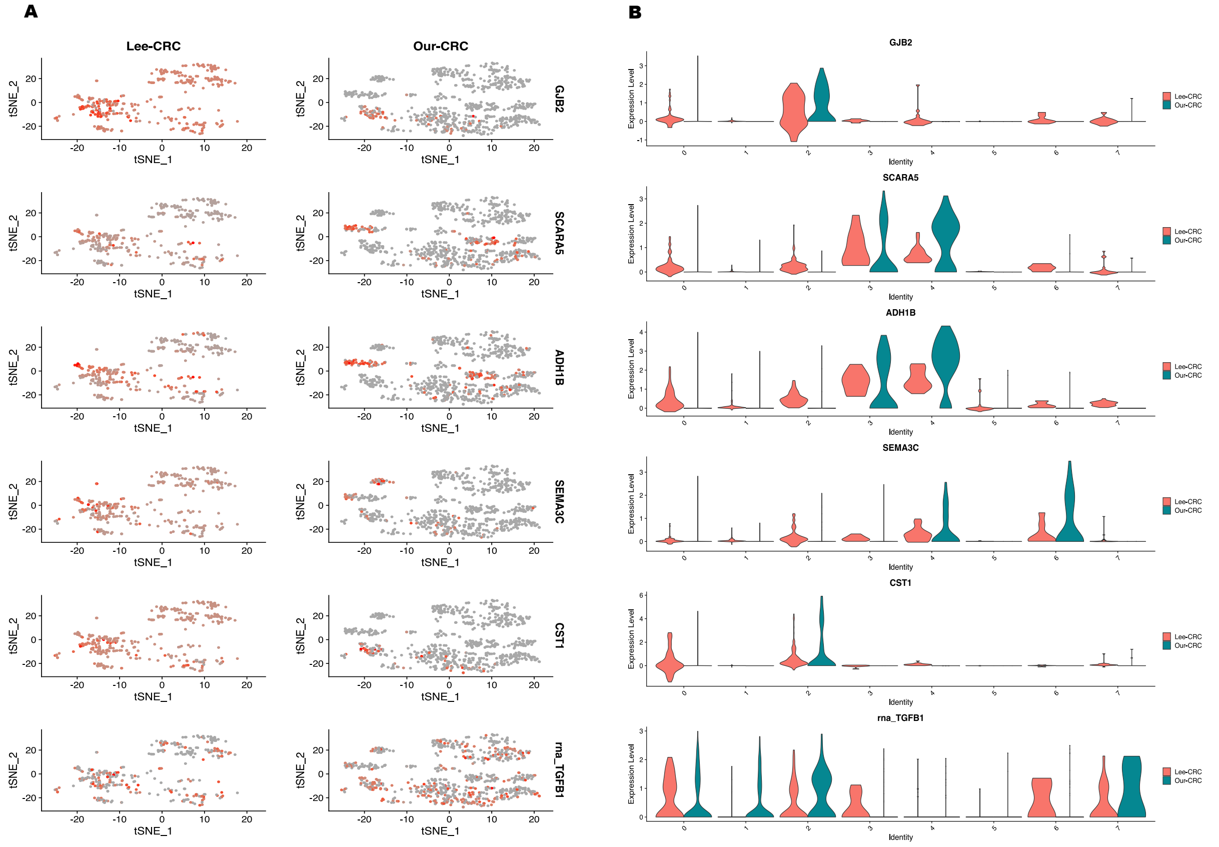

Fig. S12. Fibroblast integration analysis between Lee et al., 2020. and this study. A, comparative analysis of five CAFs subtypes from a between Lee et al., 2020 and primary CRC data. **B,** Violin plots showing the variable expression levels of five CAFs subtypes specific marker genes across integrating clusters.

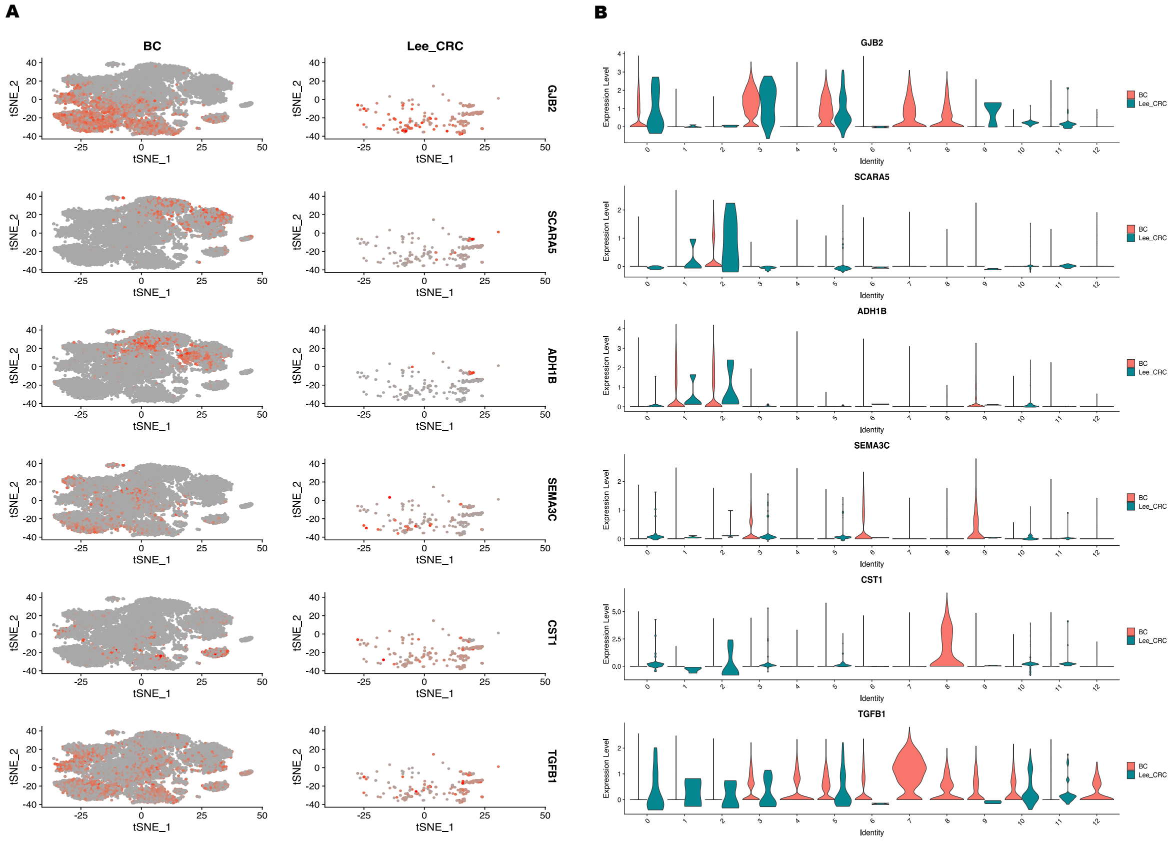

Fig. S13. Fibroblast comparative analysis between Lee et al., 2020. CRC and Breast cancer cohort. A, comparative analysis of five CAFs subtypes between breast cancer data (Kieffer et al.) and Lee et al., 2020 CRC data. **B,** Violin plots showing the variable expression levels of five CAFs subtypes specific marker genes across integrating clusters.

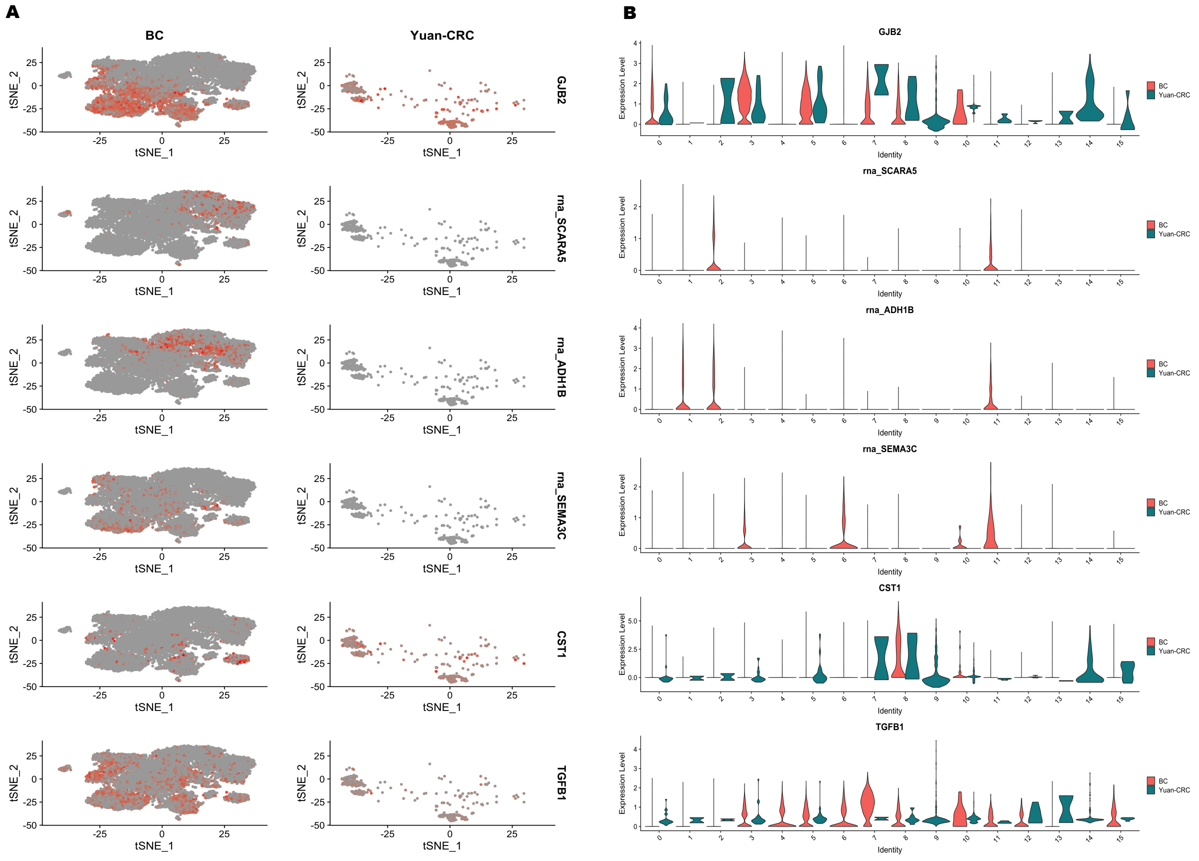

Fig. S14. Fibroblast comparative analysis between Zhou et al., 2020 CRC and Breast cancer cohort. A, comparative analysis of five CAFs subtypes between breast cancer data (Kieffer et al.) and Zhou et al., 2020 CRC data. **B,** Violin plots showing the variable expression levels of five CAFs subtypes specific marker genes across integrating clusters.

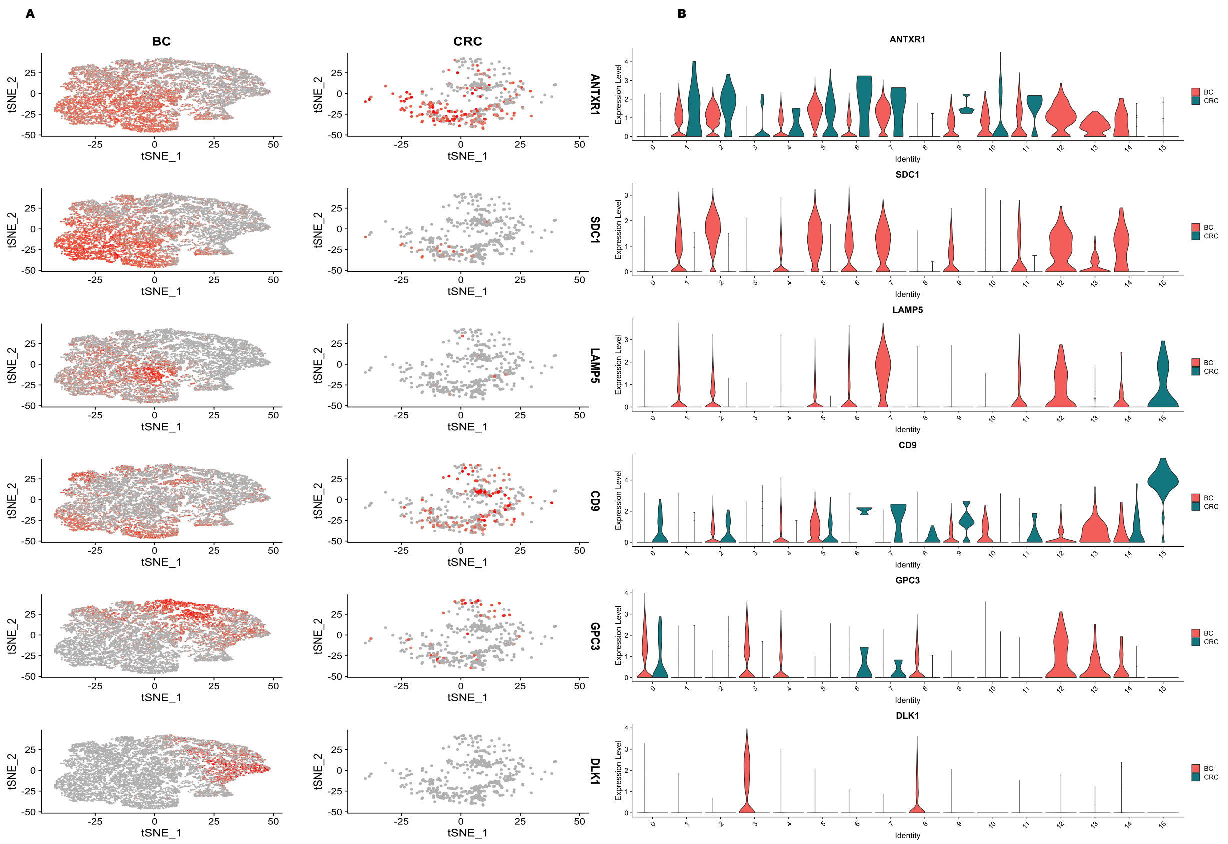

Fig. S15. Fibroblast comparative analysis between this study and Breast cancer cohort. A, comparative analysis of five CAFs subtypes between breast cancer data (Kieffer et al.) and primary CRC data. **B,** Violin plots showing the variable expression levels of five CAFs subtypes specific marker genes across integrating clusters.

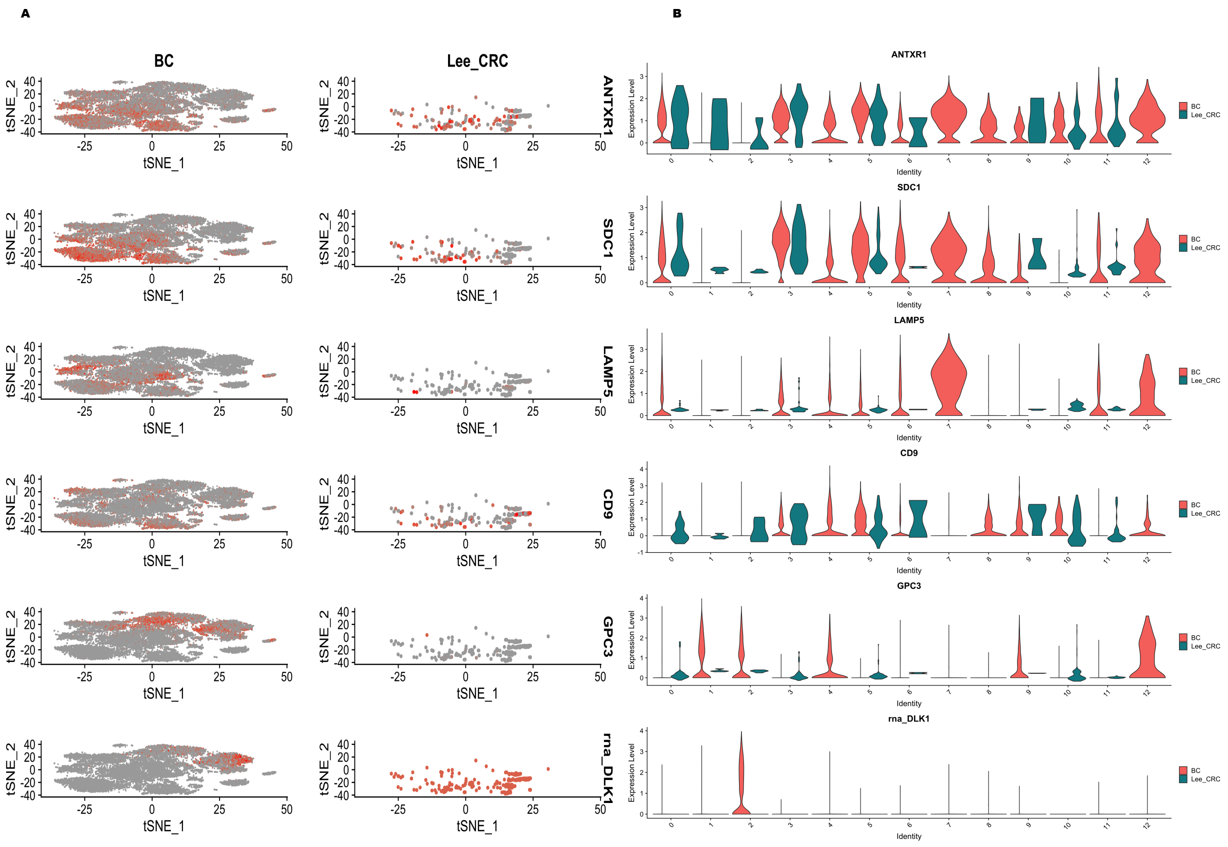

Fig. S16. Fibroblast comparative analysis between Lee et al., 2020. CRC and Breast cancer cohort. A, comparative analysis of five CAFs subtypes between breast cancer data (Kieffer et al.) and Lee et al., 2020 CRC data. **B,** Violin plots showing the variable expression levels of five CAFs subtypes specific marker genes across integrating clusters.

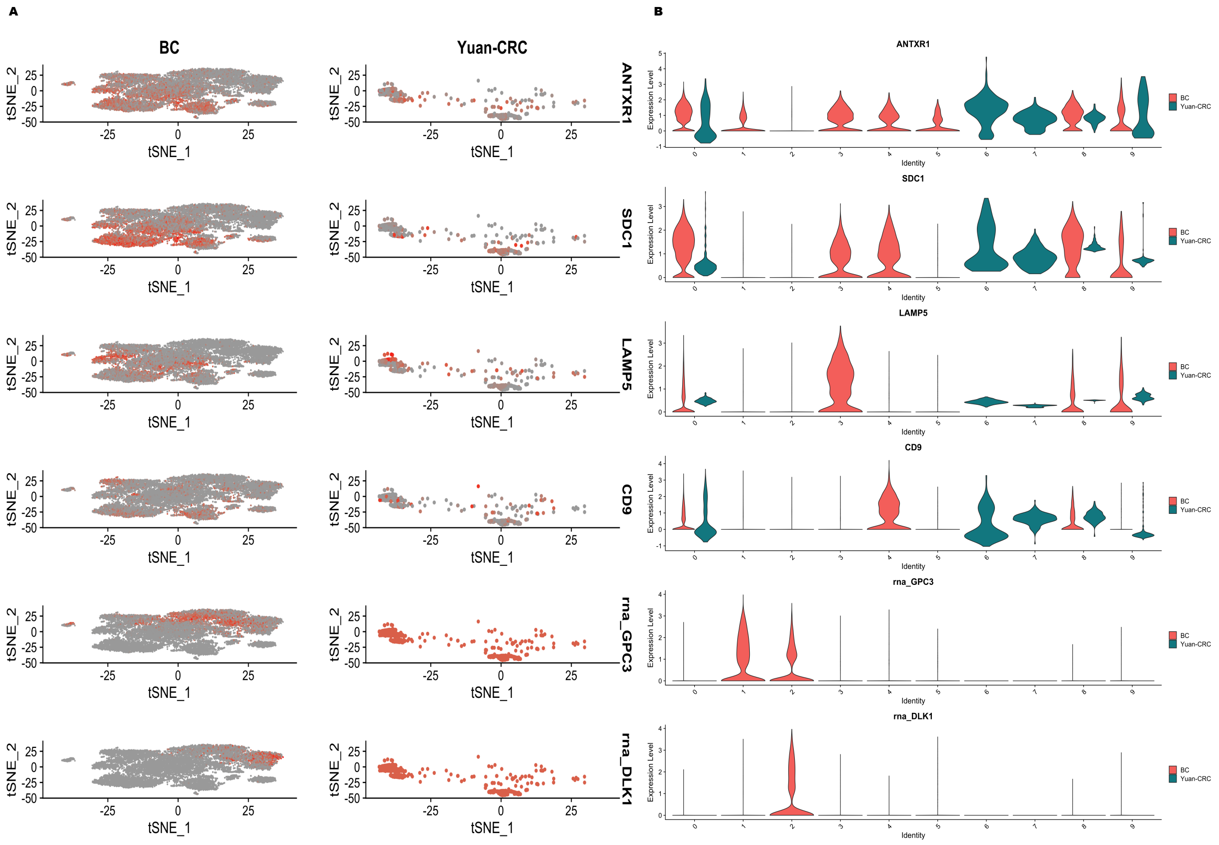

Fig. S17. Fibroblast comparative analysis between Zhou et al., 2020 CRC and Breast cancer cohort. A, comparative analysis of five CAFs subtypes between breast cancer data (Kieffer et al.) and Zhou et al., 2020, CRC data. **B,** Violin plots showing the variable expression levels of five CAFs subtypes specific marker genes across integrating clusters.

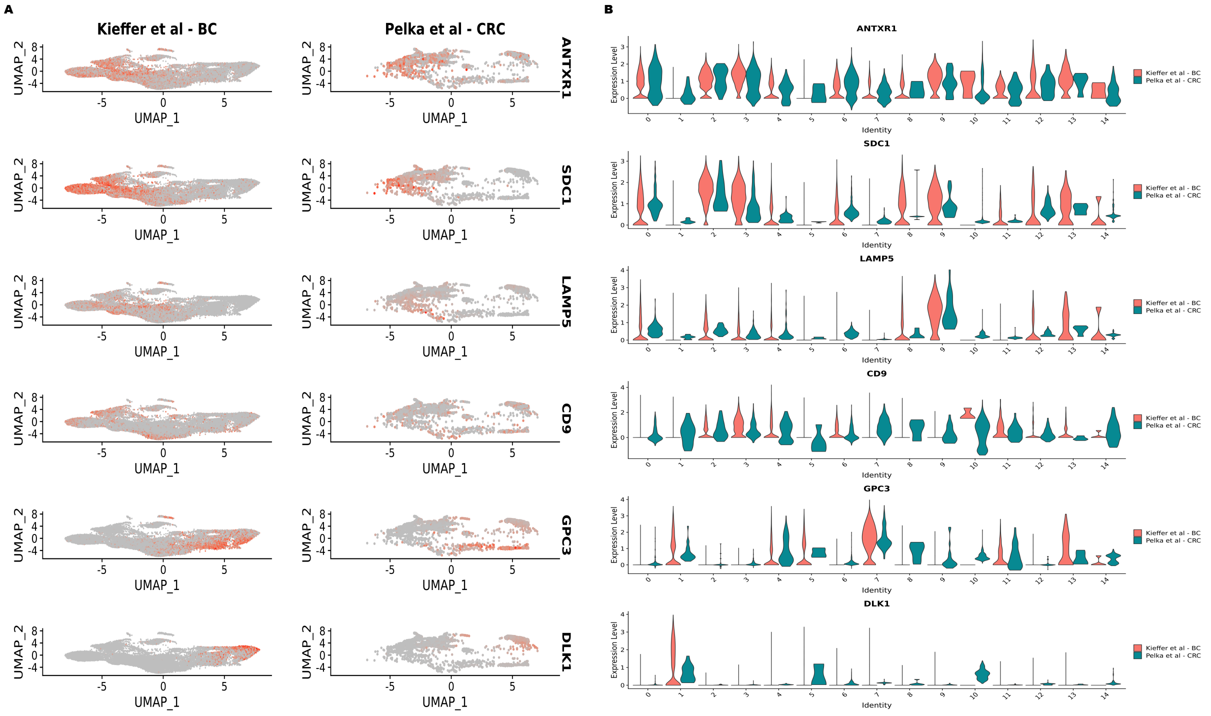

Fig. S18. Fibroblast comparative analysis between Pelka et al., 2021 CRC and Breast cancer cohort. A, comparative analysis of five CAFs subtypes between breast cancer data (Kieffer et al.) and Pelka et al., 2021 CRC data. **B,** Violin plots showing the variable expression levels of five CAFs subtypes specific marker genes across integrating clusters.

A

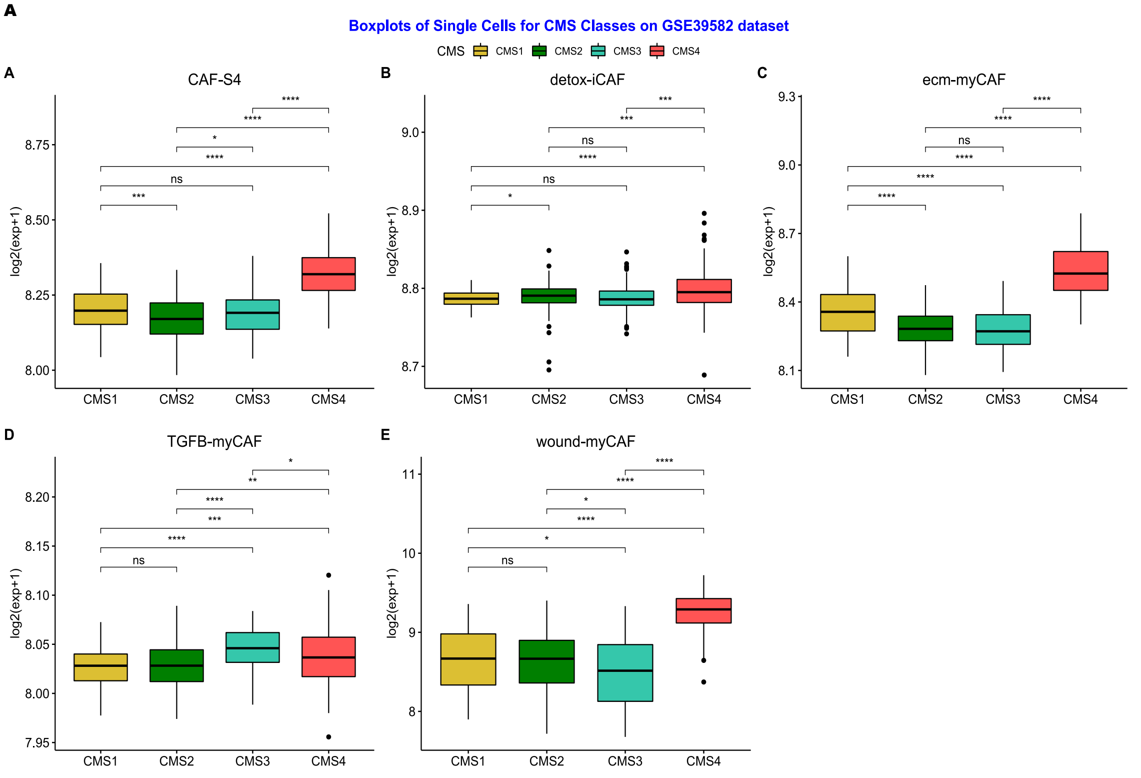

*, P < 0.05; **, P < 0.001; ***, P < 0.0001 and ****, P < 0.00001; ns, not significant.

B

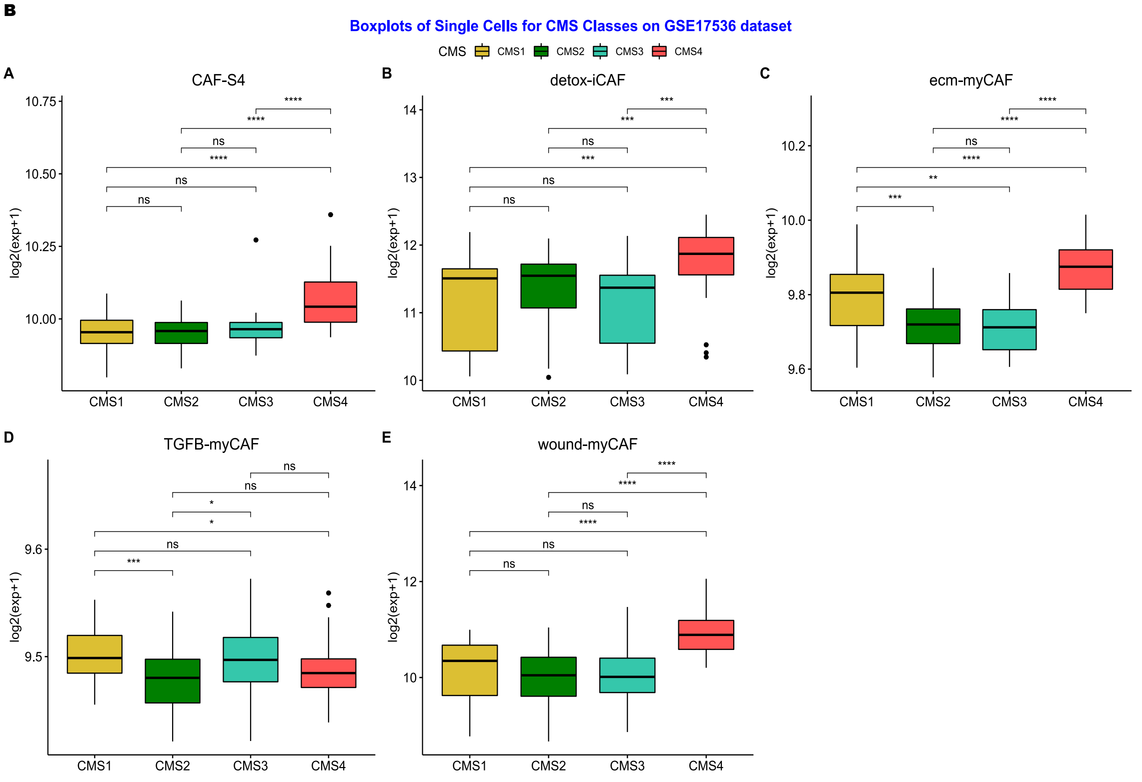

*, P < 0.05; **, P < 0.001; ***, P < 0.0001 and ****, P < 0.00001; ns, not significant.

Fig. S19. Boxplots show the distribution of CAFs. A, Distribution of fibroblast subtypes in GSE39582 CRC bulk expression datasets, based on CIBERSORTx within tumors based on CMS status. The whiskers depict the 1.5 x IQR. **B,** Distribution of fibroblast subtype in GSE17536 CRC bulk expression datasets based on CIBERSORTx, within tumors based on CMS status. The whiskers depict the 1.5 x IQR. Note 'NS': P>0.05, *:P </=0.05, **:P </=0.01, ***:P </=0.001, ****:P </=0.0001.

A

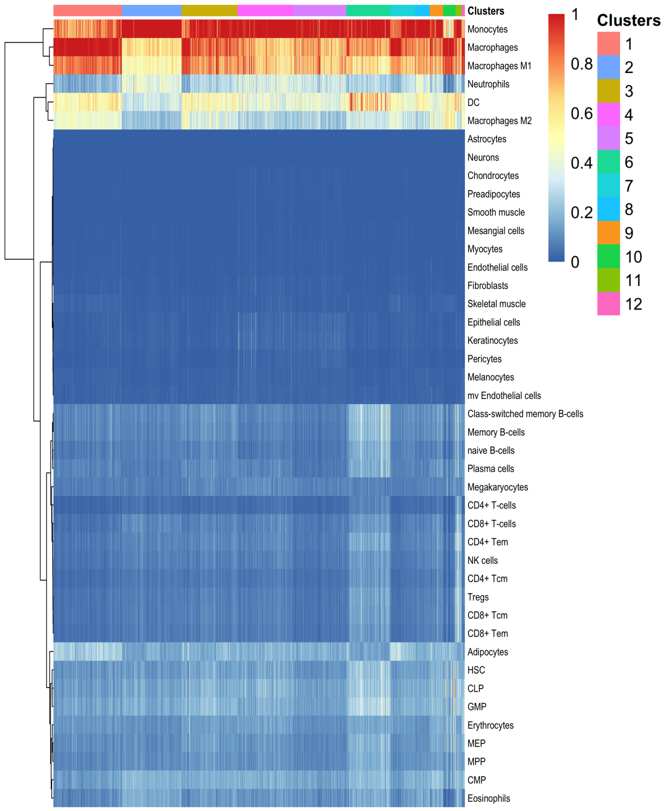

Fig. S20. Reclustering of myeloid cell compartment. SingleR heatmap demonstration of myeloid subtype distribution in primary CRC data.

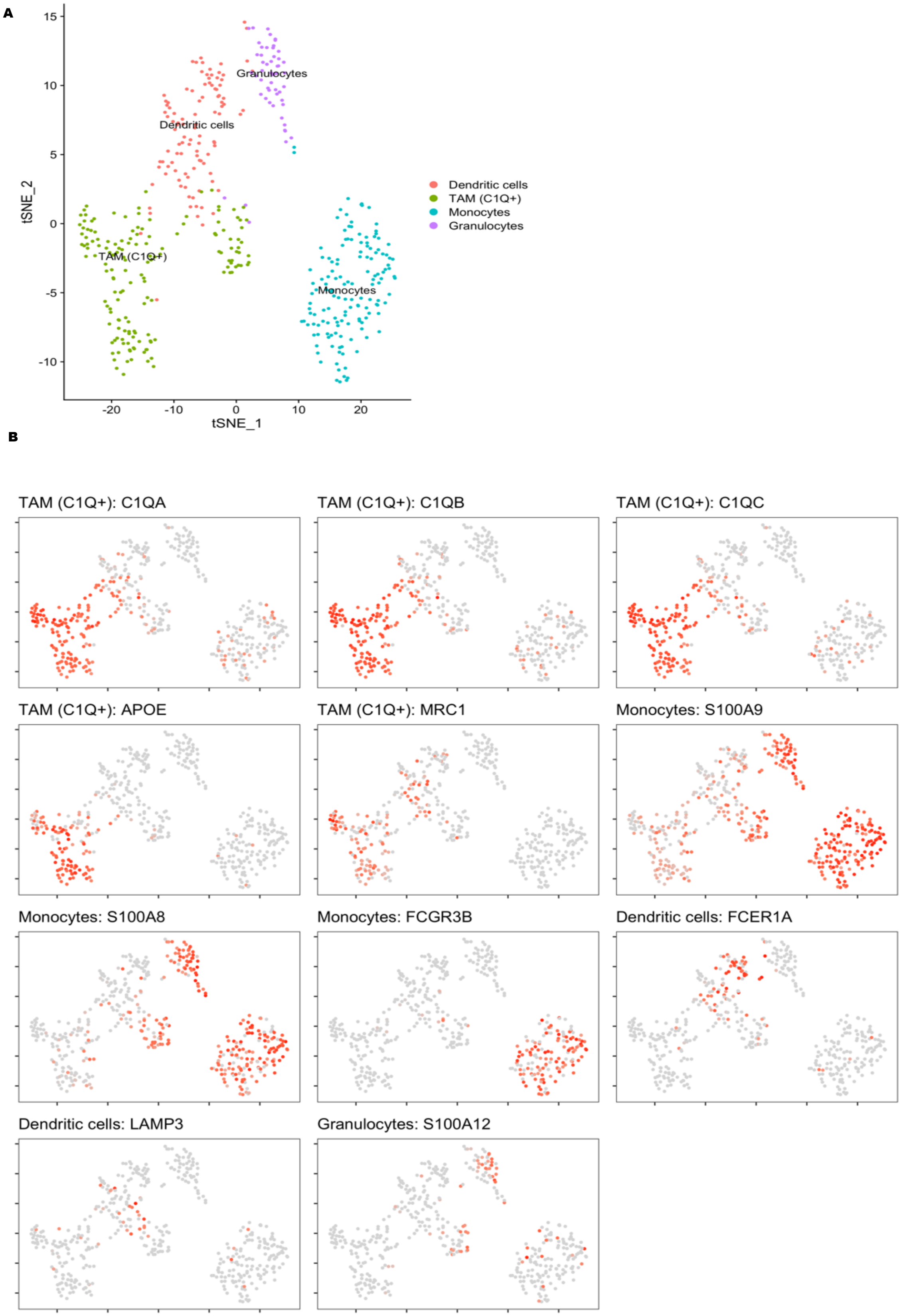
A
Granulocytes
Dendritic cells
TAM (C1Q+)
Monocytes
tSNE_2
tSNE_1
15
10
5
0
-5
-10
-20
-10
0
10
20
Dendritic cells
TAM (C1Q+)
Monocytes
Granulocytes
B
TAM (C1Q+): C1QA
TAM (C1Q+): C1QB
TAM (C1Q+): C1QC
TAM (C1Q+): APOE
TAM (C1Q+): MRC1
Monocytes: S100A9
Monocytes: S100A8
Monocytes: FCGR3B
Dendritic cells: FCER1A
Dendritic cells: LAMP3
Granulocytes: S100A12

Fig. S21. Myeloid cell clusters in Zhou et al., 2020 CRC. A, t-SNE of myeloid cells colored by distinct subtypes **B**, Identification of various myeloid cell subtypes based on expression of specified marker genes.

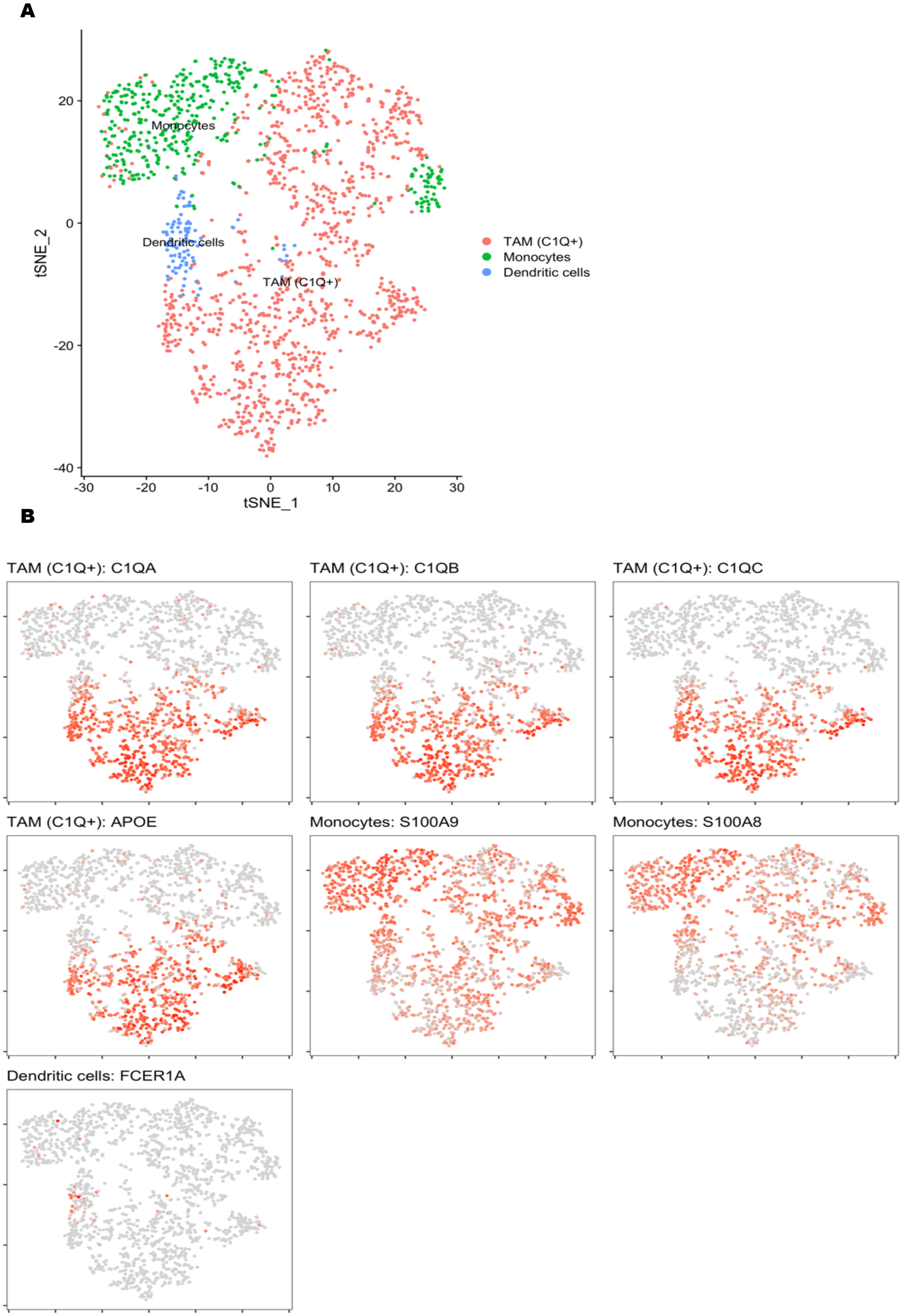
A
Monocytes
Dendritic cells
TAM (C1Q+)
tSNE_2
tSNE_1
20
0
-20
-40
-30
-20
-10
0
10
20
30
TAM (C1Q+)
Monocytes
Dendritic cells
B
TAM (C1Q+): C1QA
TAM (C1Q+): C1QB
TAM (C1Q+): C1QC
TAM (C1Q+): APOE
Monocytes: S100A9
Monocytes: S100A8
Dendritic cells: FCER1A

Fig. S22. Myeloid cell clusters in Lee et al., 2020., 2020 CRC. A, t-SNE of myeloid cells colored by distinct clusters. **B**, Identification of various myeloid cell subtypes based on expression of specified marker genes.

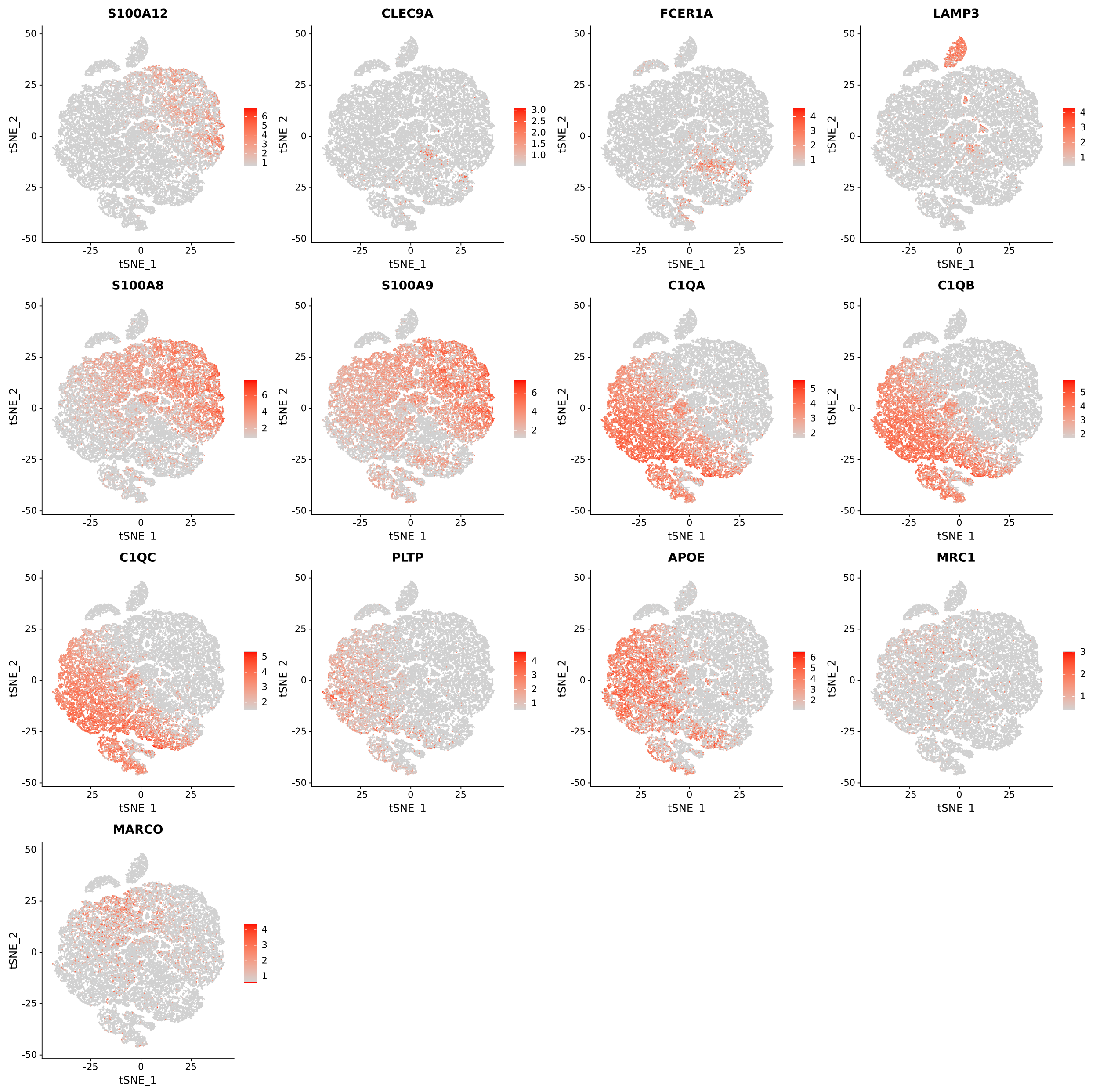

Fig. S23. Myeloid cell clusters in Pelka et al., 2021 CRC. t-SNE Identification of various myeloid cell subtypes based on expression of specified marker genes.

A

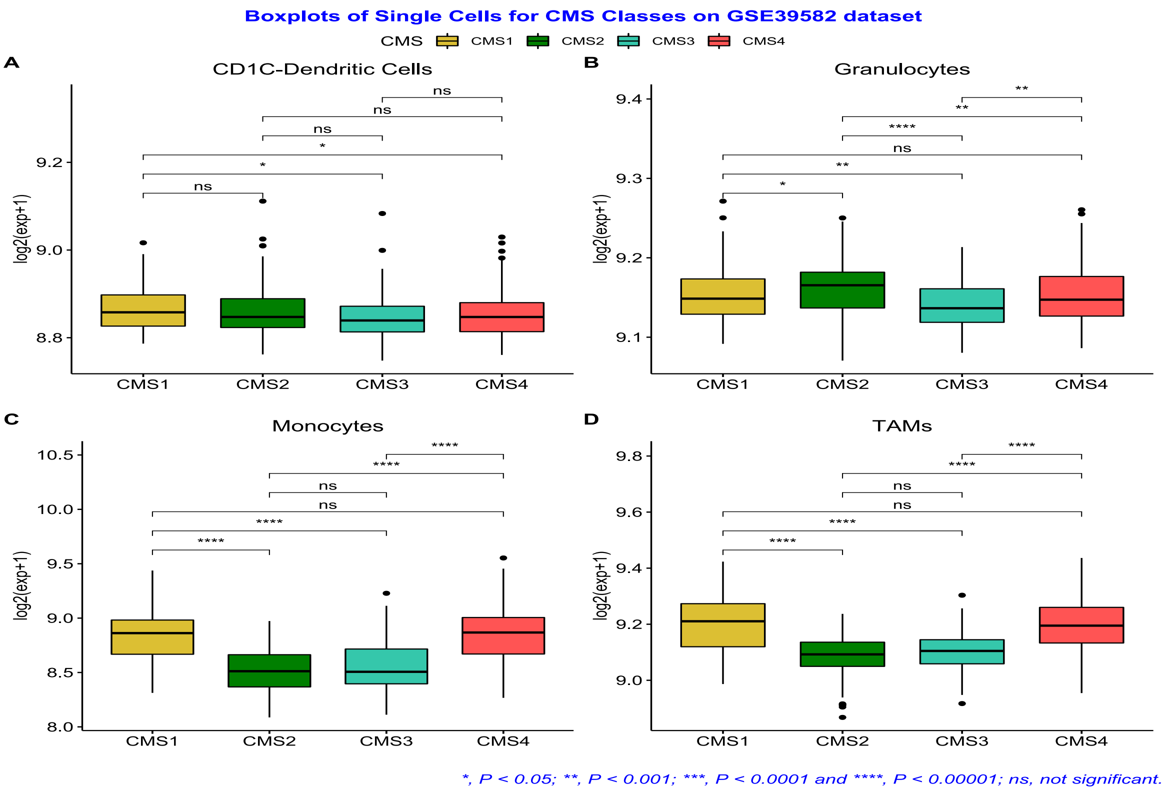
Boxplots of Single Cells for CMS Classes on GSE39582 dataset
CMS CMS1 CMS2 CMS3 CMS4
A
CD1C-Dendritic Cells
B
Granulocytes
C
Monocytes
D
TAMs
log2(exp+1)
CMS1 CMS2 CMS3 CMS4
*, P < 0.05; **, P < 0.001; ***, P < 0.0001 and ****, P < 0.00001; ns, not significant.

B

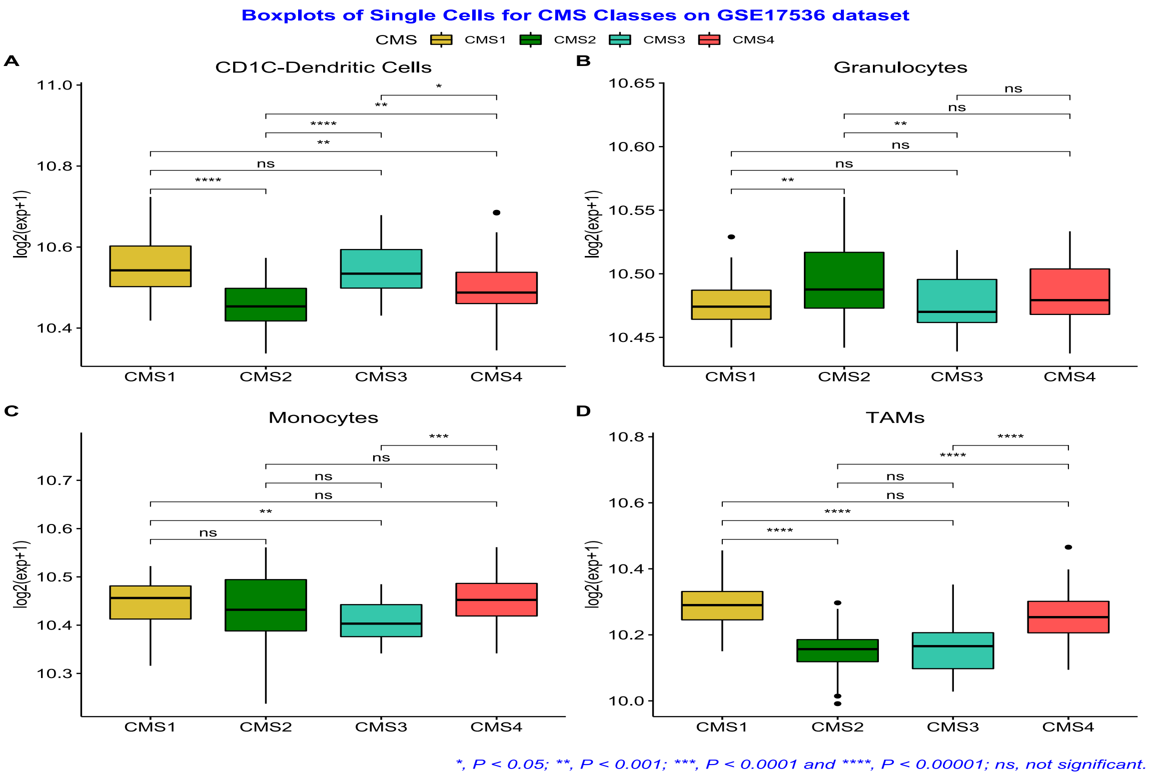
Boxplots of Single Cells for CMS Classes on GSE17536 dataset
CMS CMS1 CMS2 CMS3 CMS4
A
CD1C-Dendritic Cells
B
Granulocytes
C
Monocytes
D
TAMs
log2(exp+1)
CMS1 CMS2 CMS3 CMS4
*, P < 0.05; **, P < 0.001; ***, P < 0.0001 and ****, P < 0.00001; ns, not significant.

Fig. S24. Boxplots show the distribution of cell types. A, Distribution of myeloid subtypes in GSE39582 CRC bulk expression datasets obtained by CIBERSORTx, within tumors based on CMS status. The whiskers depict the 1.5 x IQR. **B,** Distribution of myeloid subtype in GSE17536 CRC bulk expression datasets, within tumors based on CMS status. The whiskers depict the 1.5 x IQR. Note ‘NS’: P>0.05, *:P </=0.05, **:P </=0.01, ***:P </=0.001, ****:P </=0.0001.

A

B

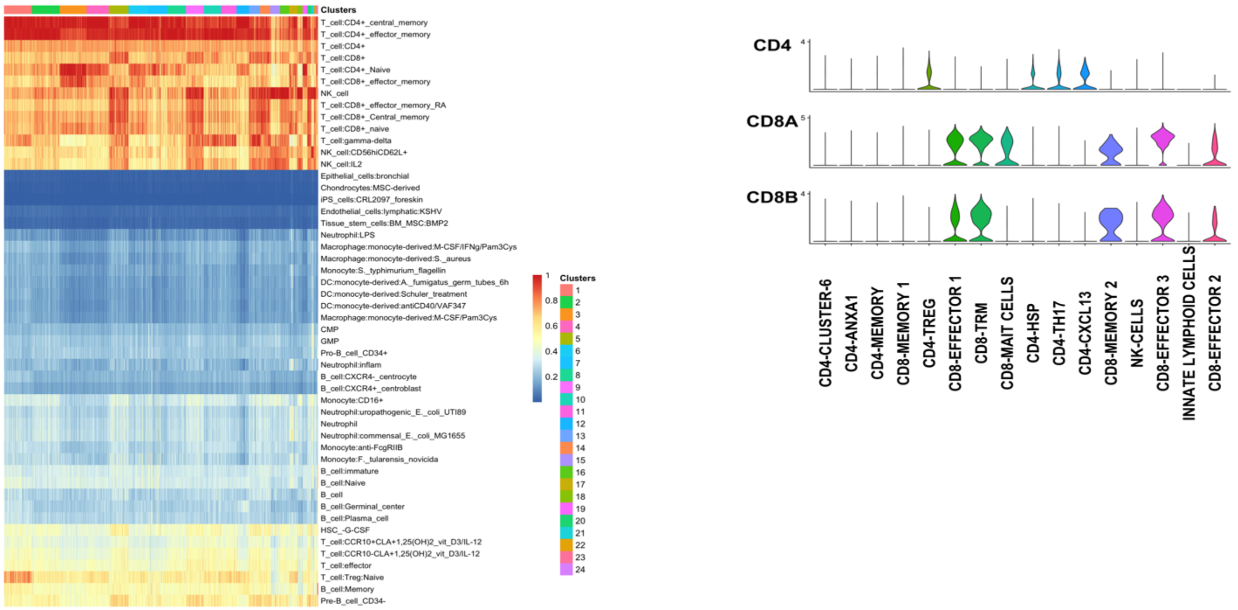

Fig. S25. T cell identification and characterization. A, SingleR heatmap cell type identification within each cluster. Note that genes associated with T cells show increased expressions, confirming the quality of the data. Note doublets were removed from the further analysis. **B,** Violin plots showing the differential expression of T cell specific marker genes between CD4 and CD8 phenotypes.

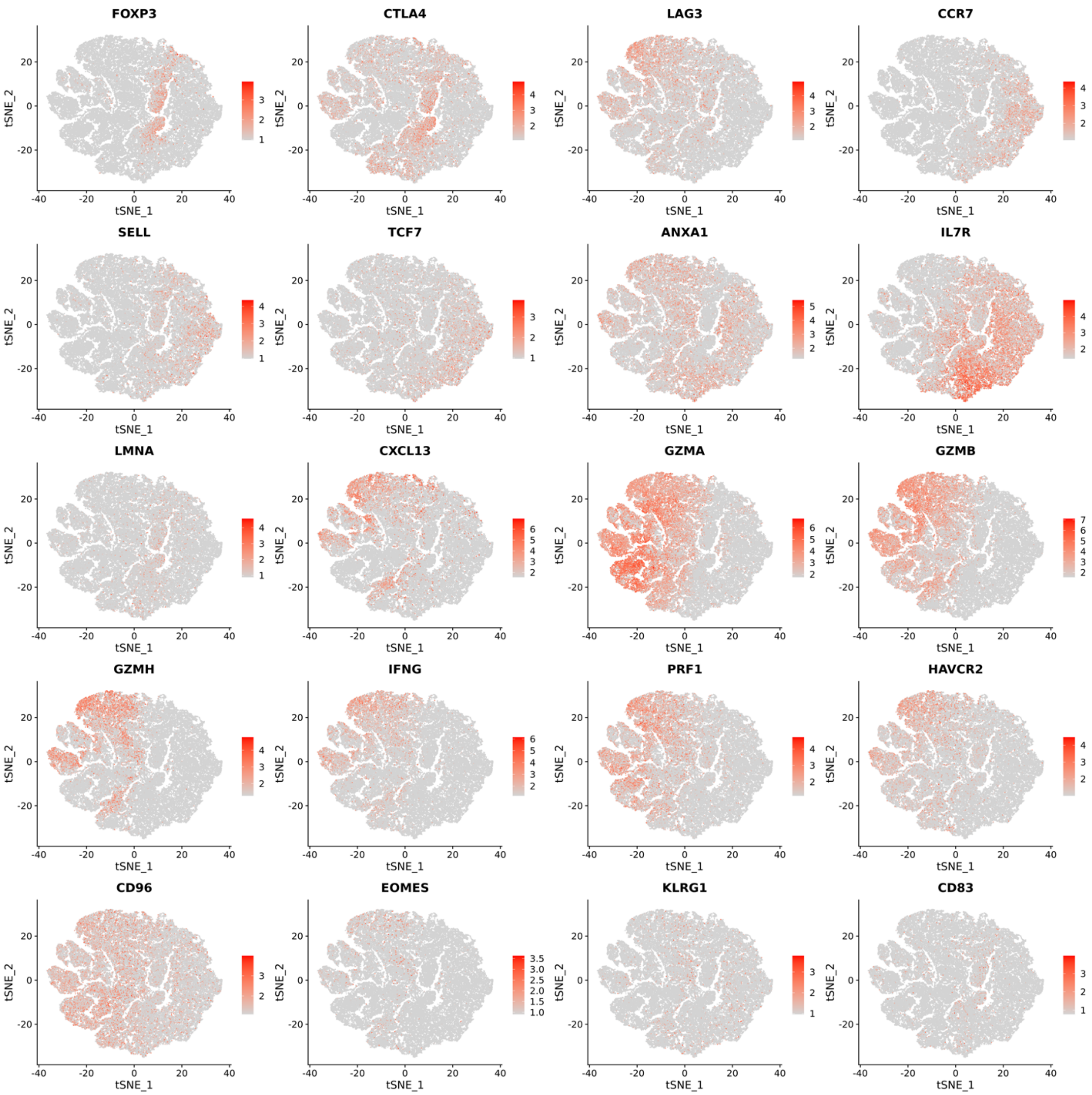

Fig. S26. T-cell clusters in Pelka et al., 2021 CRC. tSNE showing the variable expression of T-cell specific marker genes across various clusters.

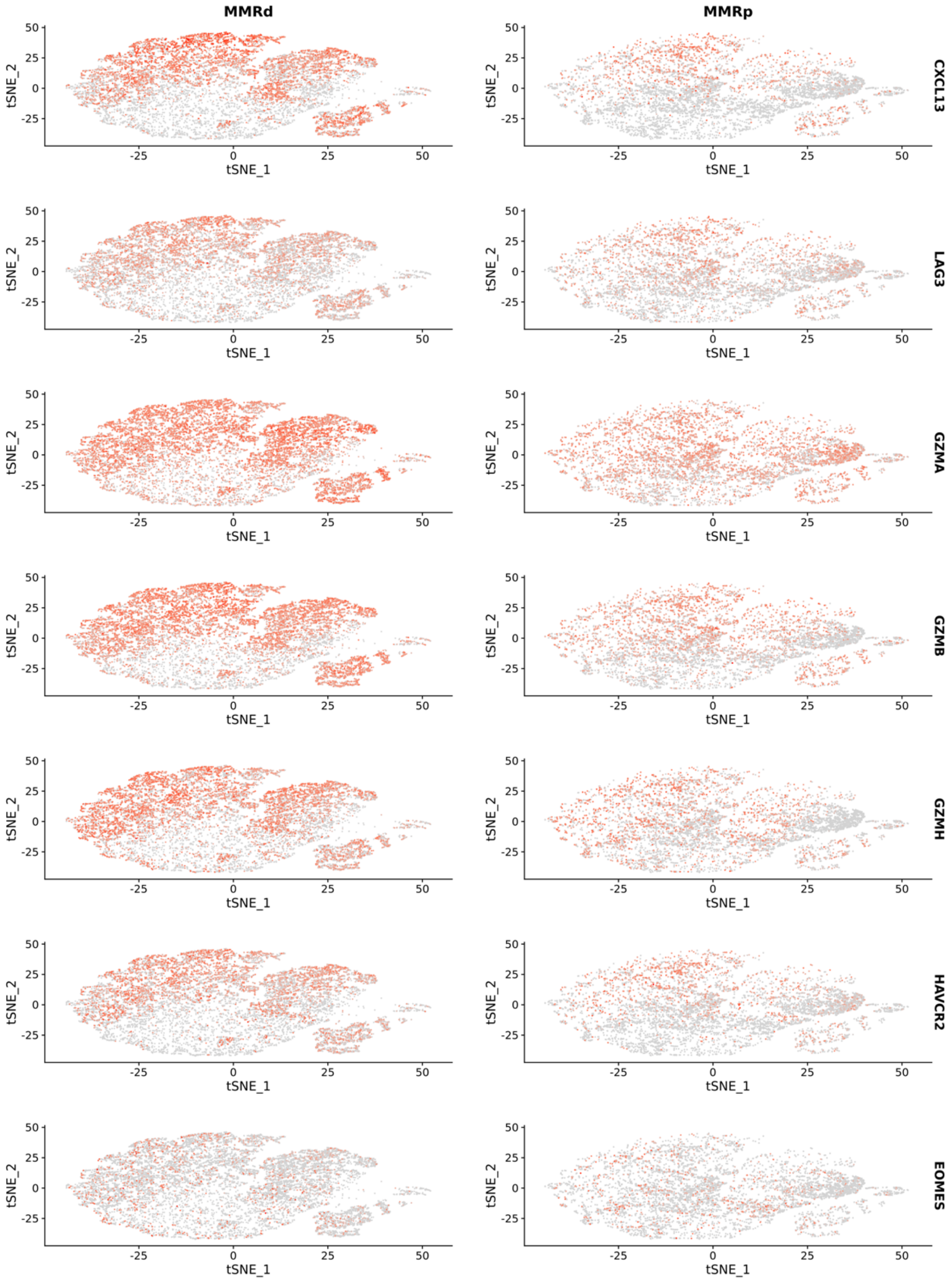

Fig. S27. t-SNE of CD8+ T-cells showing the variable expression of CD8+ cytotoxic T-Cell specific marker genes between MSS and MSI-H tumors.

A

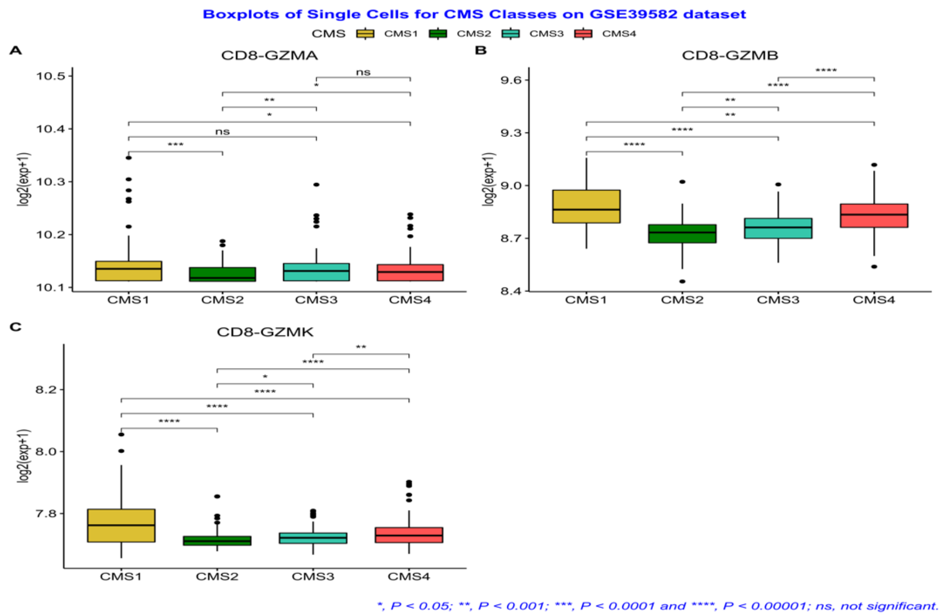

B

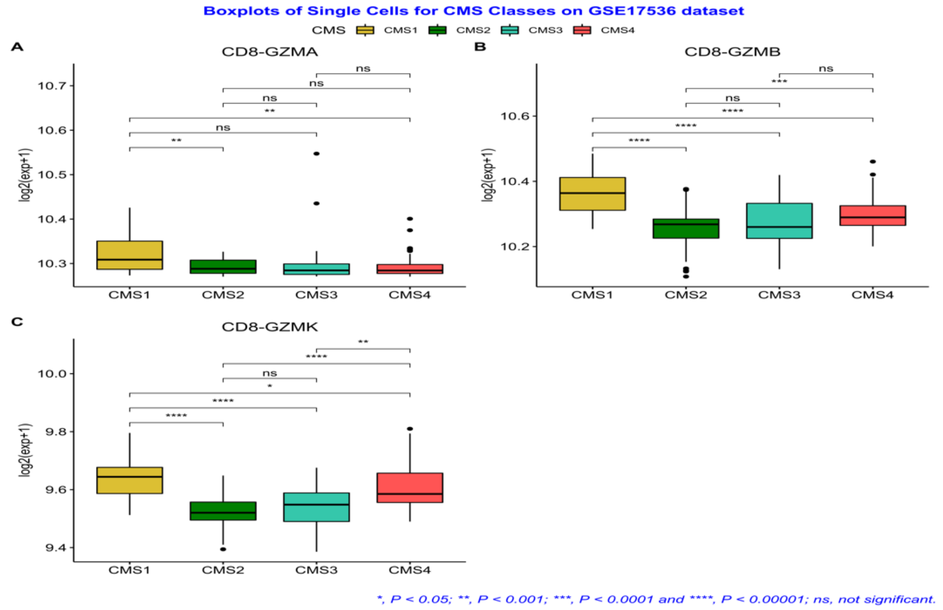

Fig. S28. Boxplots show the distribution of cell types. A, Distribution of T-cells subtypes in GSE39582 CRC bulk expression datasets based on CIBERSORTx, within tumors based on CMS status. The whiskers depict the 1.5 x IQR. **B,** Distribution of T-cells subtype in GSE17536 CRC bulk expression datasets, within tumors based on CMS status. The whiskers depict the 1.5 x IQR. Note 'NS': P>0.05, *:P </=0.05, **:P </=0.01, ***:P </=0.001, ****:P </=0.0001.

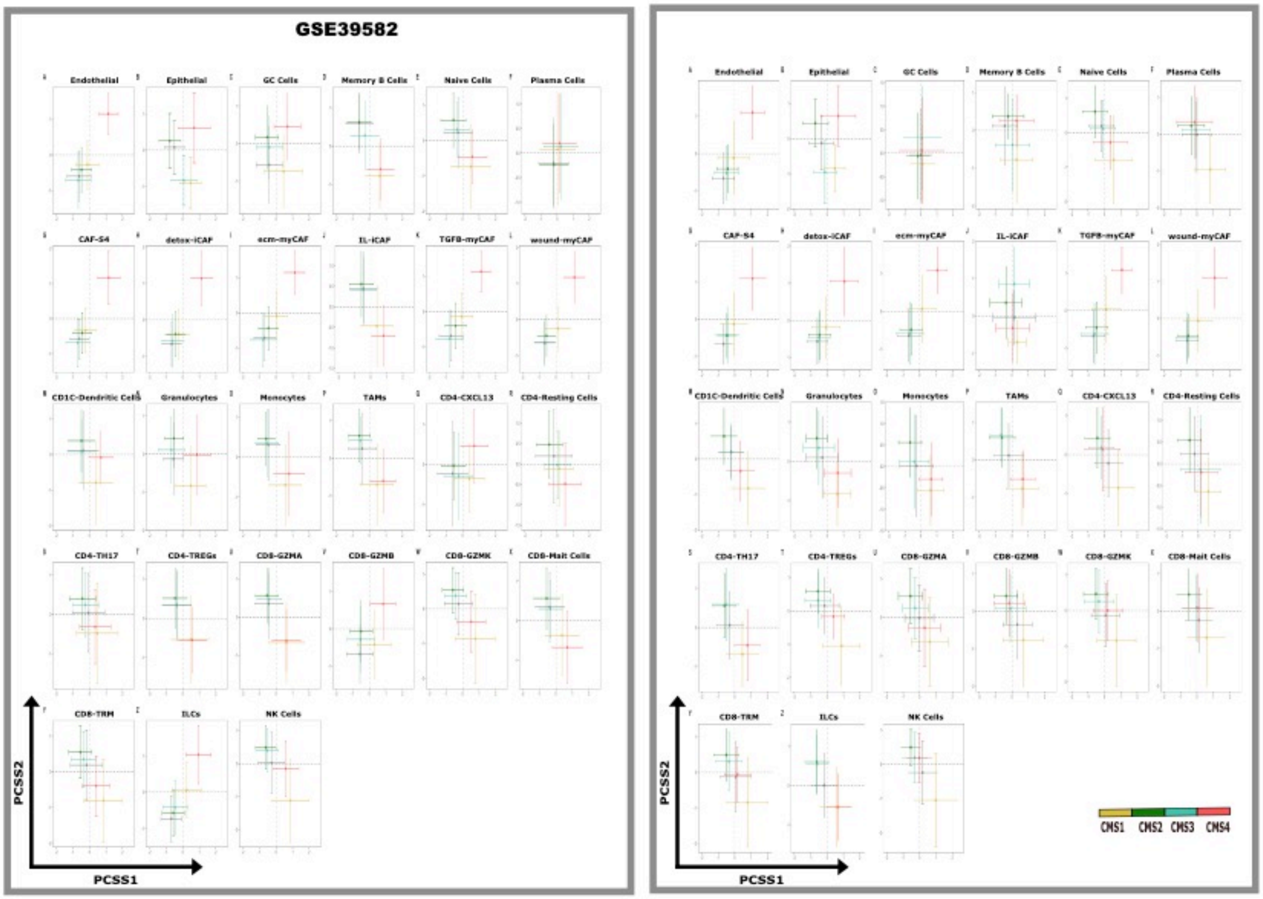

Fig. S29. Continuous subtype scoring across cell type (GSE39582, GSE1736). A, Continuous scores reported by CMS classification across cell types show minimal separation in the top 2 principal components in GSE39582 dataset. **B,** Continuous scores reported by CMS classification across cell types show minimal separation in the top 2 principal components in GSE17536 dataset, all cell types projected on four quadrants representing CMS1-4 using PCSS1 and PCSS2 scores. Note that the cell types largely form a continuum along CMS status and are not clustered in discrete quadrants separate from one another except CAF-S4. Cells and markers are colored by bulk CMS status accordingly to the tumor sample of origin.

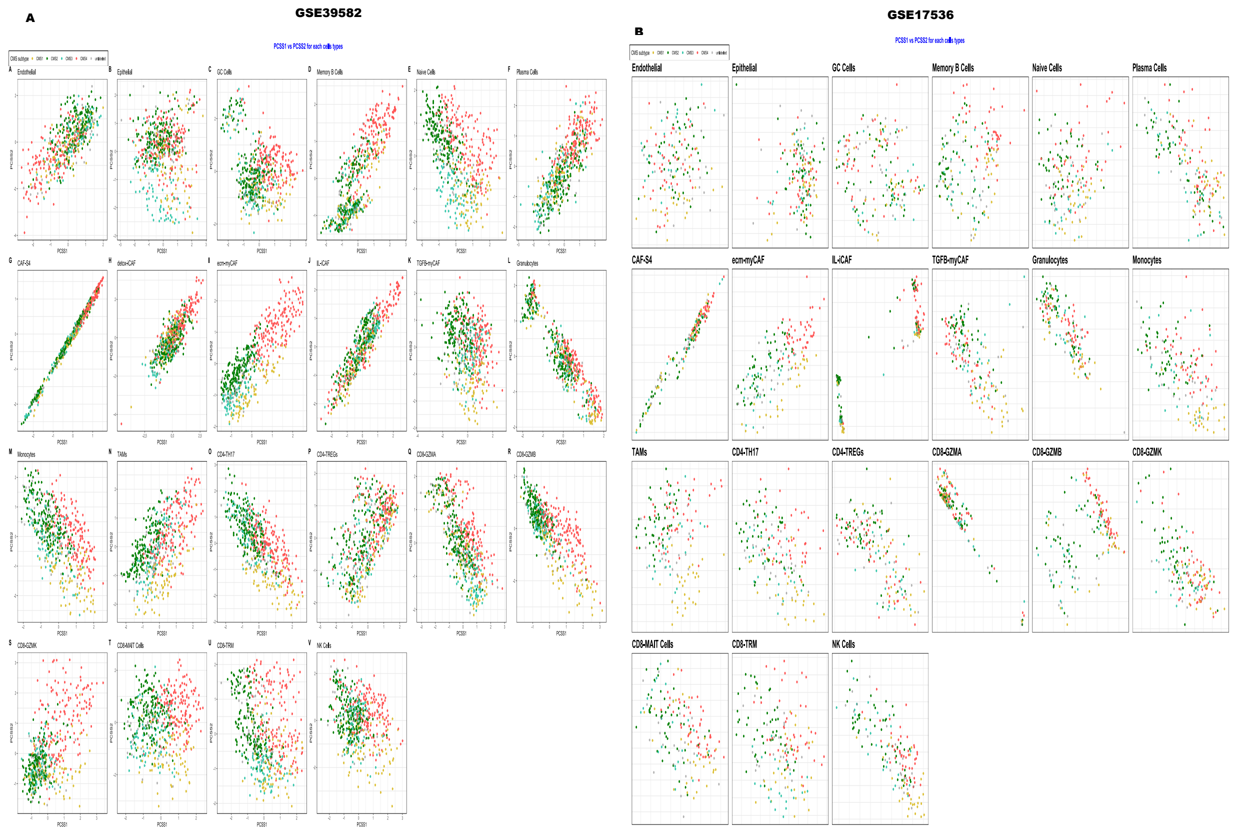

Fig. S30 Continuous subtype scoring across cell type (GSE39582, GSE1736). A-B Continuous scores reported by CMS classification across cell types show minimal separation in the top 2 principal components in GSE39582 and GSE17536 datasets respectively. Continuous scores reported by CMS classification across cell types show minimal separation in the top 2 principal components in both datasets, all cell types representing CMS1-4 using PCSS1 and PCSS2 scores. Gene signatures were obtained using CIBERSORTx algorithm.

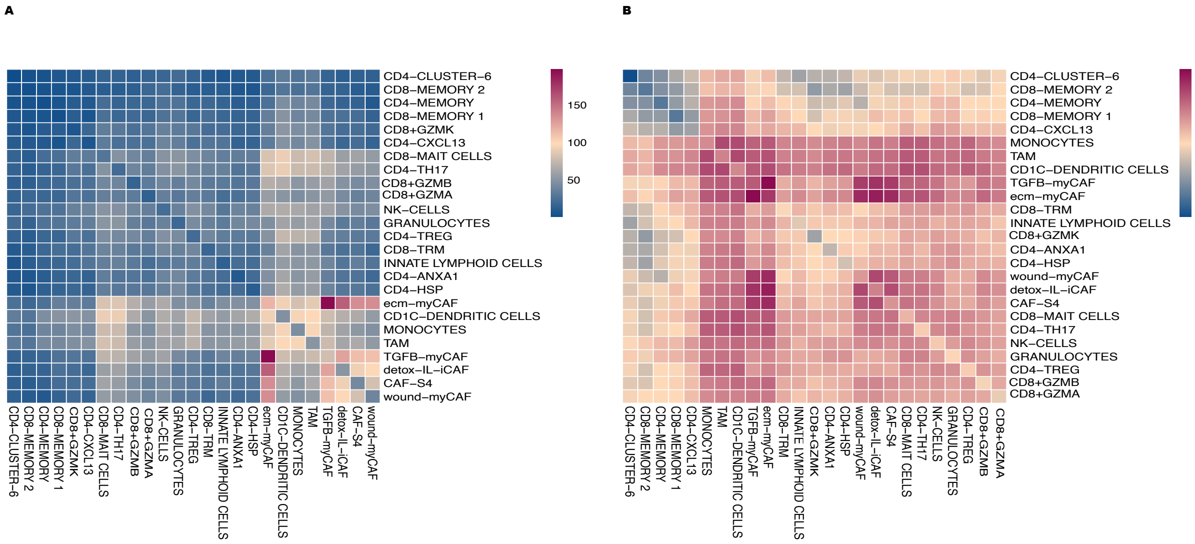

Fig. S31. Tumor and TME interactions. A, Heatmap showing the total number of interactions between various cell types in primary CRC dataset using *CellPhoneDB*. **B,** Heatmap of log normalized counts showing the total number of interactions between cell types in primary CRC dataset.

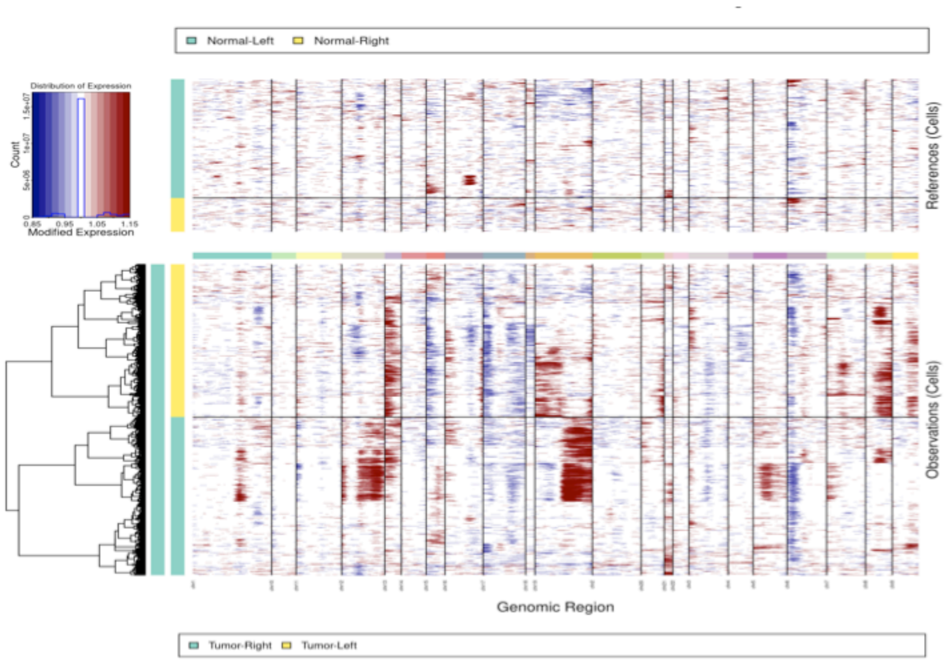

Fig. S32. Analysis of copy number variation amongst epithelial cells. A, CNV analysis was conducted on the entire epithelial cell compartment. Increase CNV is seen in tumor derived epithelial cells (observation). Non-malignant derived epithelial cells were used as control.

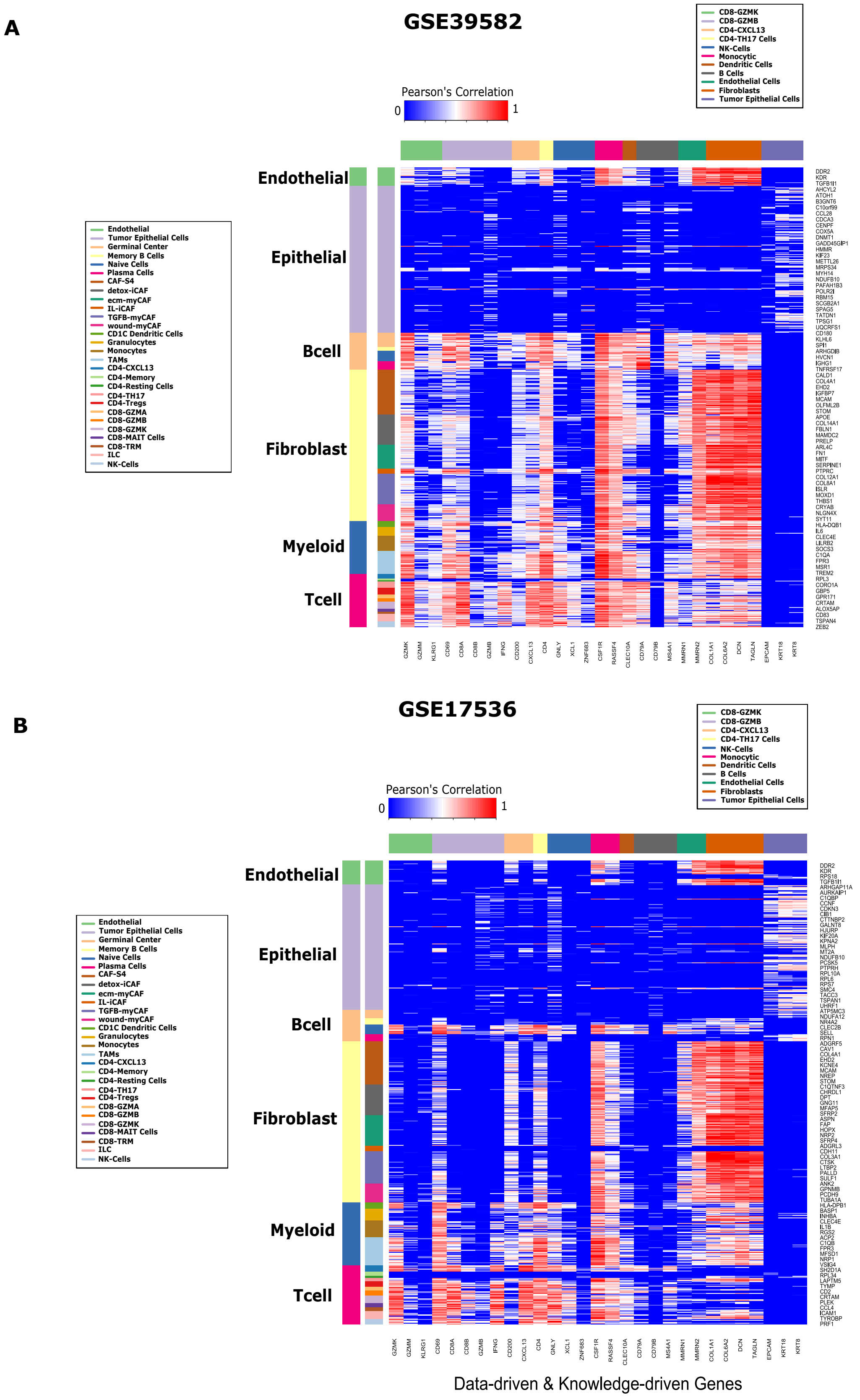
A
GSE39582
Pearson's Correlation
0
1
CD8-GZMK
CD8-GZMB
CD4-CXCL13
CD4-TH17 Cells
NK-Cells
Monocytic
Dendritic Cells
B Cells
Endothelial Cells
Fibroblasts
Tumor Epithelial Cells
Endothelial
Tumor Epithelial Cells
Germinal Center
Memory B Cells
Naive Cells
Plasma Cells
CAF-S4
detox-iCAF
ecm-myCAF
IL-iCAF
TGFB-myCAF
wound-myCAF
CD1C Dendritic Cells
Granulocytes
Monocytes
TAMs
CD4-CXCL13
CD4-Memory
CD4-Resting Cells
CD4-TH17
CD4-Tregs
CD8-GZMA
CD8-GZMB
CD8-GZMK
CD8-MAIT Cells
CD8-TRM
ILC
NK-Cells
Endothelial
Epithelial
Bcell
Fibroblast
Myeloid
Tcell
B
GSE17536
Data-driven & Knowledge-driven Genes

Fig. S33. Correlation Heatmap for the top correlated marker genes for each cell types in bulk expression data set (GSE39582, GSE17536). A, the top correlated marker genes for each cell types (all subtypes of B-cells, Endothelial, Epithelial, Fibroblast, Myeloid, and T-cells) in GSE39582 and **B,** GSE17536 bulk expression data set, the marker genes with an average log2 FC>= 0.5 and adjusted P<0.05 obtained from the Single Cell analysis of each cell type were separately intersected with the bulk gene expression sets individually. The Genes that have an average Spearman correlation score greater than 0.5 with others, were kept as the cell signatures of the corresponding cell type within the bulk gene expression. Afterwards, we removed the highly correlated genes from the cell signature gene lists, if they exist in more than one cell type to make marker list mutually exclusive. Thereby, we obtained the marker gene list that is unique for each cell subtype.

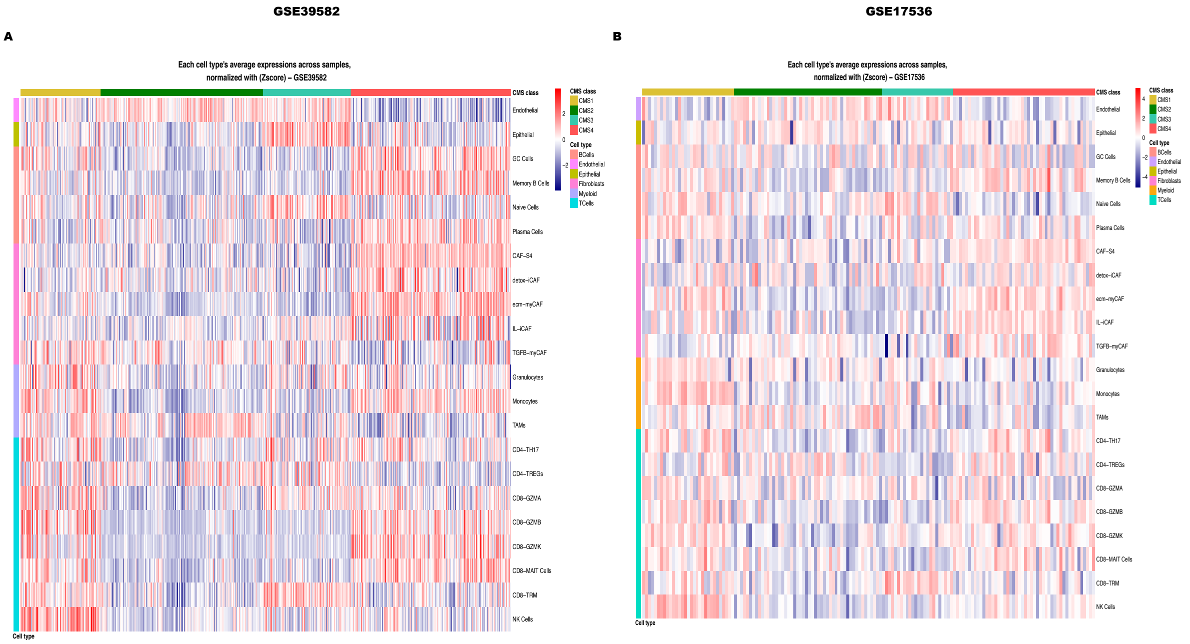

Fig. S34. CIBERSORTx results for GSE39582 (A) and GSE17536 (B). Heatmap of cell abundance predicted per sample. Row z-score are shown.

Batch-effect correction for malignant cells

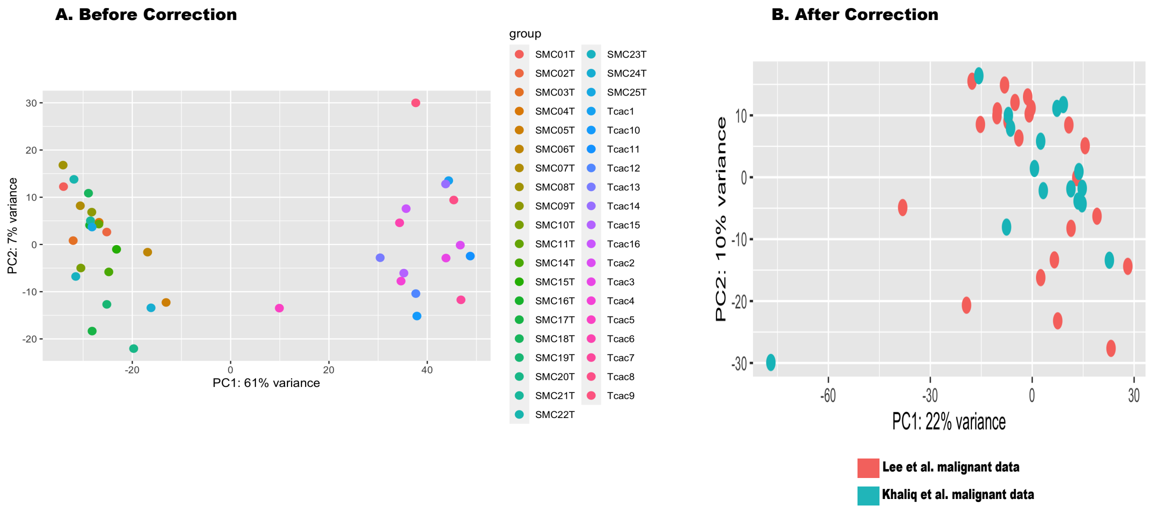

Fig. S35. Batch-effect correction for combined malignant cells. A-B, PCA showing before and after batch correction of merged tumor cells across the Lee et al., 2020 (n = 23) and our CRC (n = 16) using *canonical correlation analysis* (CCA) and *Limma* method (see methods)**.**
