# Supplementary material for: Refining colorectal cancer classification and clinical stratification through a single-cell atlas
Source: Genome Biol. 2022 May 11;23:113. doi: 10.1186/s13059-022-02677-z (PMC9092724; doi:10.1186/s13059-022-02677-z)
Supplement: Supplementary file 15 — Additional file 15. Review history. [file 13059_2022_2677_MOESM15_ESM.docx]

**Review History**

**First round of review**

**Reviewer 1**

**Were you able to assess all statistics in the manuscript, including the appropriateness of statistical tests used?**

Yes

**Comments to author:**

Overall Impression:
In this article Khaliq et al analyze a new cohort of primary, untreated CRC profiled with single cell RNAseq (n = 15 tumors) and re-analyze a previously published single cell study of a similar untreated, primary patient population. Two additional bulkseq profiled datasets are included for validation. Primary findings are that: (1) bulkseq derived CMS of CRC show significant intratumor heterogeneity, (2) specific CAF populations exist in CRC and are associated with poor survival, (3) C1Q+ TAM are associated with CD8 T cell dysfunction and poor survival. The findings related to CAF and TAM are particularly novel and lead directly to potential therapeutic strategies, and the finding related to heterogeneity within CMS although previously observed has not been described with this level of detail. Overall I am supportive of publication of this manuscript, but have a few majors concerns that would need to be addressed.

Major Comments:
1) Assignment of CMS: I find the use of pseudo-bulk expression profiles for CMS assignment to be troubling as to my knowledge the CMS, which were derived from bulk transcriptomes of untreated primary CRC tumors, have never been validated on pseudo-bulk transcriptomes. It is well known that the single cell dissociation process preferentially enriches for certain cell types (such as immune cells) and depletes other (notably hepatocytes) as these cells differ in their ability to survive the dissociation. Given that CMS assignments are known to be influenced by the percentage of immune and stromal cells (Chowdhury et al., 2021), it is likely that using pseudo-bulk for CMS assignment will yield a different result than bulk transcriptome. Given that bulk transcriptomes appear to be available for the samples with single cell, revising the CMS calls should be relatively easy.
2) Reference to prior studies of intratumor heterogeneity and CMS: Other papers have looked at the implication of intratumor heterogeneity on CMS, notably (Chowdhury et al., 2021), (Piskol et al., 2019), and (Laurent-Puig et al., 2018). These should be referenced.
3) Support of claims of "high quality" single cell data. At multiple points in main text the authors claim that the higher quality of their single cell data relative to that of the Lee et al study. However, reviewing the on-line methods it is not clear to me what has been done differently vs. Lee et al. This needs to be explicitly noted in the main text. It is possible I missed this in the supplement, but since this is a relatively central and important part of the paper a clear distinction needs to be highlighted in main text. With regard to what can be considered a gold standard for single cell RNAseq, I would consider (Slyper et al., 2020) to the best. Specific to CRC, the standard can be considered to be (Pelka et al., 2021) which is the largest single cell cohort published to date.
The initial cut offs used:
"Genes detected in fewer than three cells and cells expressing less than 200 detected genes were filtered out and excluded from analysis. In addition, cells expressing > 25% mitochondria were removed"

Seem reasonable although the 25% cutoff for mitochondria may be removing cells of adequate quality (Pelka et al., 2021).
4) Reporting of outcomes data: Not nearly enough detail is provided to interpret the clinical outcome data provided. The CRC cohorts need to be better identified and described (treated, untreated, stage, ect). Additionally, a multivariate analysis should be performed with other factors known to influence survival, such as age, stage, KRAS / BRAF mutational status, ect, and the effect on survival should be reported as a Hazard Ratio.
5) Evaluation of mutational status of known driver genes: APC, KRAS/NRAS, BRAF, TP53, ect with presence of specific cell types. Although the authors have clearly made some interesting findings, a look into how the key driver mutations influence the immune and stromal reaction would be very interesting and seems possible the available data.
6) Lack of inclusion of Broad CRC single cell dataset: Given that this cohort contains ~ 60 matched tumor and normal samples with single cell RNAseq, I was surprised that it was not used as a validation cohort.

Minor Comments:
1) line 94 - would mention exceptional cases of IO for MSI-H tumors, combination EGFR/BRAF inhibition for BRAF V600E mutant tumors
2) line 190 - why is Wnt activity unexpected, especially for CMS2 tumors?
3) line 195 - would also reference (Kunal Rai)
4) line 337 - need more description of these cohorts in main text: stage? Pre/post treatment? Primary or metastatic tumors?
5) Figure 2A is confusing, does not seem to align with the caption. Legend does not describe the labels, which I think correspond to patients
6) Figure 3E - too small to view in current format, would make larger

Chowdhury, S., Hofree, M., Lin, K., Maru, D., Kopetz, S., and Shen, J.P. (2021). Implications of intratumor heterogeneity on consensus molecular subtype (CMS) in colorectal cancer. Cancers 13, 4923.
Laurent-Puig, P., Marisa, L., Ayadi, M., Blum, Y., Balogoun, R., Pilati, C., Le Malicot, K., Lepage, C., Emile, J.F., Salazar, R., et al. (2018). Colon cancer molecular subtype intratumoral heterogeneity and its prognostic impact: An extensive molecular analysis of the PETACC-8. Annals of Oncology 29, viii18.
Pelka, K., Hofree, M., Chen, J.H., Sarkizova, S., Pirl, J.D., Jorgji, V., Bejnood, A., Dionne, D., Ge, W.H., Xu, K.H., et al. (2021). Spatially organized multicellular immune hubs in human colorectal cancer. Cell 184, 4734-4752.e4720.
Piskol, R., Huw, L., Sergin, I., Kljin, C., Modrusan, Z., Kim, D., Kljavin, N., Tam, R., Patel, R., Burton, J., et al. (2019). A Clinically Applicable Gene-Expression Classifier Reveals Intrinsic and Extrinsic Contributions to Consensus Molecular Subtypes in Primary and Metastatic Colon Cancer. Clinical Cancer Research 25, 4431-4442.
Slyper, M., Porter, C.B.M., Ashenberg, O., Waldman, J., Drokhlyansky, E., Wakiro, I., Smillie, C., Smith-Rosario, G., Wu, J., Dionne, D., et al. (2020). A single-cell and single-nucleus RNA-Seq toolbox for fresh and frozen human tumors. Nature Medicine 26, 792-802.

**Reviewer 2**

**Were you able to assess all statistics in the manuscript, including the appropriateness of statistical tests used?**

Yes

**Comments to author:**

In this manuscript, "Refining Colorectal Cancer Classification And Clinical Stratification Through A Single- Cell Atlas" Masood and collogues present a single-cell atlas of Colorectal Cancer (CRC). They profiled ~50k cells from 16 CRC tumours and eight adjacent normal tissues. A key finding of this manuscript includes the identification of fibroblast and macrophage populations and its implications in predicating CMS subtypes. In addition, they also report the association between a subset of CD8+ T-cells and its importance in predicting immunotherapy response in CMS1 (MSI-H) CRCs. In the last few years, multiple groups have reported the single-cell atlas of CRCs. This manuscript has integrated this information/data to increase the patient number and provide more generalised observations in CRCs. Overall this is an interesting manuscript and provides some interesting observations, specifically potential targeting of CAFs and C1Q TAMs to improve IO response. However, the lack of experimental validation and suboptimal analysis of bulk RNA-seq data dampens this reviewer's enthusiasm. I suggest the author employ CIBERSORTx or other deconvolution tools to analyse bulk RNA-seq and identify why higher CAF and C1Q TAMs lead to poor outcomes (cell-cell interaction analysis). Moreover, it is essential to validate these findings in retrospective patient samples (FFPE blocks) to substantiate these claims.
- In the past couple of years, multiple major scRNA-seq publications have been reported in the area of CRC (PMID: 32302573, PMID: 32451460, PMID: 34450029, PMID: 34467243 etc.). Authors have done a good job in integrating Lee et al. in their study. Still, they have not included another major atlas in their analysis, and it's not clear whether the contradiction between Lee et al. and this manuscript is consistent across other studies or specific to patient populations and experimental conditions.
Authors should also perform additional trajectory analysis such as CellRank (PMID: 35027767), which is more specific for cancer cells to compare Lee et al., and this manuscript's cell continuum observation.
- Authors have done a good job of including bulk RNA-seq in their analysis for validation etc.; however, their analysis methods are not very optimal and they should consider employing CYBERSORTx (PMID: 31061481) or EcoTyper (PMID: 34597589) to deconvolute bulk RNA-seq and validate scRNA-seq observations in bulk data.
- It is interesting to see the correlation between CAFs, TAM and poor outcomes in CRC. It's not sure if these cells are spatially enriched in TME or CRC and what kind of immunosuppressive interactions occur between them, which might play an important role in IO response

**Authors Response**

**Point-by-point responses to the reviewers’ comments:**

*We appreciate the thoughtful review and insightful critiques by the Reviewers. In response to the reviewer’s concerns and suggestion, we have substantially revised the manuscript. Specifically, we have incorporated additional studies comprising 372,327 of cells. In total we have analyzed 487,829 single cells in this study and carried out additional analyses consisting of 1) Trajectory analysis on additional dataset and performed another trajectory method using Slingshot 2) Multivariate analysis on two independent CRC cohorts 3) CIBERSORTx for deconvolution of bulk transcriptomics data 4) Cell-cell interaction analysis. Overall, we found that while our conclusions remain unchanged, the revisions strengthened and improved the quality of the manuscript.*

Reviewer #1:

Overall Impression:
In this article Khaliq et al analyze a new cohort of primary, untreated CRC profiled with single cell RNAseq (n = 15 tumors) and re-analyze a previously published single cell study of a similar untreated, primary patient population. Two additional bulkseq profiled datasets are included for validation. Primary findings are that: (1) bulkseq derived CMS of CRC show significant intratumor heterogeneity, (2) specific CAF populations exist in CRC and are associated with poor survival, (3) C1Q+ TAM are associated with CD8 T cell dysfunction and poor survival. The findings related to CAF and TAM are particularly novel and lead directly to potential therapeutic strategies, and the finding related to heterogeneity within CMS although previously observed has not been described with this level of detail. Overall, I am supportive of publication of this manuscript, but have a few majors concerns that would need to be addressed.

*Response: We want to thank Reviewer for acknowledging our work and providing rigorous attention to important details to our work. Our paper has improved because of the Reviewer’s critical feedback.*

Major Comments:
1) Assignment of CMS: I find the use of pseudo-bulk expression profiles for CMS assignment to be troubling as to my knowledge the CMS, which were derived from bulk transcriptomes of untreated primary CRC tumors, have never been validated on pseudo-bulk transcriptomes. It is well known that the single cell dissociation process preferentially enriches for certain cell types (such as immune cells) and depletes other (notably hepatocytes) as these cells differ in their ability to survive the dissociation. Given that CMS assignments are known to be influenced by the percentage of immune and stromal cells (Chowdhury et al., 2021), it is likely that using pseudo-bulk for CMS assignment will yield a different result than bulk transcriptome. Given that bulk transcriptomes appear to be available for the samples with single cell, revising the CMS calls should be relatively easy.

*Response: We agree with the reviewer that Pseudobulk transcriptomics have not been validated for CMS classification and therefore assigning CMS classification based on Pseudobulk transcriptomics will produce inconsistent results. As the reviewer correctly stated, CMS assignment can be modified by the quantity of immunological and stromal cells present, as demonstrated by Chowdhary et al., 2021. We respectfully, want to clarify that CMS calls on tumor cells are based on matched bulk-transcriptomics data in our analysis. Following that, Pseudobulk analysis was performed in accordance with previous single-cell studies in ovarian and renal cell carcinoma [1,2] . We would like to express our gratitude to the reviewer for pointing out this critical distinction, and we have revised the sentence to make it clearer to our readers.*

2) Reference to prior studies of intratumor heterogeneity and CMS: Other papers have looked at the implication of intratumor heterogeneity on CMS, notably (Chowdhury et al., 2021), (Piskol et al., 2019), and (Laurent-Puig et al., 2018). These should be referenced.

*Response: Thank you for bringing this to our attention. These references have now been incorporated in the manuscript.*

3) Support of claims of "high quality" single cell data. At multiple points in main text the authors claim that the higher quality of their single cell data relative to that of the Lee et al study. However, reviewing the on-line methods it is not clear to me what has been done differently vs. Lee et al. This needs to be explicitly noted in the main text. It is possible I missed this in the supplement, but since this is a relatively central and important part of the paper a clear distinction needs to be highlighted in main text. With regard to what can be considered a gold standard for single cell RNAseq, I would consider (Slyper et al., 2020) to the best. Specific to CRC, the standard can be considered to be (Pelka et al., 2021) which is the largest single cell cohort published to date.
The initial cut offs used:
"Genes detected in fewer than three cells and cells expressing less than 200 detected genes were filtered out and excluded from analysis. In addition, cells expressing > 25% mitochondria were removed"
Seem reasonable although the 25% cutoff for mitochondria may be removing cells of adequate quality (Pelka et al., 2021).

*Response: We appreciate the reviewer's input. We acknowledge that previous work such as Slyper et al. 2020 are excellent guides for single cell quality control. We also concur that the filtering methods described by the reviewer are essential but would like to emphasize that data-cutoffs for single cell data are study dependent. Additionally, cells that exhibit hybrid markers must be eliminated from downstream analysis. We incorporated and expanded the methodology section to fully describe our study and emphasized the reviewer’s suggestions in the main text. Our analysis is further depicted in Supplementary Figure S2-S5, where cells expressing hybrid cell type markers were manually removed before downstream analysis. Additionally, for full transparency, we have made the quality control metrics and dataset available to reviewers and readers via GitHub:* <https://github.com/masoodlab/CRC-Single-Cell/>

4) Reporting of outcomes data: Not nearly enough detail is provided to interpret the clinical outcome data provided. The CRC cohorts need to be better identified and described (treated, untreated, stage, ect). Additionally, a multivariate analysis should be performed with other factors known to influence survival, such as age, stage, KRAS / BRAF mutational status, ect, and the effect on survival should be reported as a Hazard Ratio.

*Response: The reviewer raises important points and we therefore performed a multivariate. As shown in supplementary Table 12, CAFs and C1Q+ TAM were strong independent prognosticators of short DFS, adjusted by clinical features, pathological stage, chemotherapy and common mutational status further supporting our conclusions of CAFs and TAMs in CRC pathobiology. We have included and described these new results in the manuscript. Also, we have included clinical data, demographics, stage, chemotherapy, and key mutations in a new Table 1. in the manuscript.*

5) Evaluation of mutational status of known driver genes: APC, KRAS/NRAS, BRAF, TP53, ect with presence of specific cell types. Although the authors have clearly made some interesting findings, a look into how the key driver mutations influence the immune and stromal reaction would be very interesting and seems possible the available data.

*Response: We appreciate the reviewer's acknowledgment of our current study's interesting and novel findings. We agree with the reviewer that examining the impact of key driver mutations, such as the gatekeeper APC, and other key RAS-RAF-RTK mutations on specific cell types would provide unique insights into CRC biology. However, we wish to emphasize that we unfortunately are unable to assess these mutations due to Pelka et al. and Zhou et al. not including these annotations in their single cell data sets in contrast to our annotated data set. In future studies, we aim to increase the sample size of our single cell data sets with full somatic mutation annotations to examine this important idea in greater detail.*

6) Lack of inclusion of Broad CRC single cell dataset: Given that this cohort contains ~ 60 matched tumor and normal samples with single cell RNAseq, I was surprised that it was not used as a validation cohort.

*Response: The reviewer’s suggestion has been well taken and Pelka et al., dataset have included in the manuscript as recommended by the reviewer as a validation cohort. The results from Pelka et al., and all other datasets were concordant further validating our study. We were able to unravel all CAF subtypes, C1Q+ TAMs and T-cell heterogeneity including CD8+ cytotoxic cells mainly enriched in MSI-H CRCs which are the key findings of our study.*

Minor Comments:
1) line 94 - would mention exceptional cases of IO for MSI-H tumors, combination EGFR/BRAF inhibition for BRAF V600E mutant tumors.

*Response: We appreciate the reviewer bringing these important therapies to our notice. These therapies are now described in our manuscript.*

2) line 190 - why is Wnt activity unexpected, especially for CMS2 tumors?

*Response: We apologize for the mistake in the manuscript. Our analysis showed CMS3 and CMS4 Wnt enrichment that was unexpected based on CMS classification. However, these results are consistent with Sadanadan et al., epithelial based classification where they showed CRC subtypes are associated with distinct anatomical locations of the colon crypts (phenotype), as well as differentiation states with Wnt signaling activity varied according to location (Figure below)[3]. In agreement with sadanandan et al, our data showed high Wnt-activity in CMS3(goblet like cell subtype)e and CMS4 (stemlike subtype)[4,5]. We appreciate the reviewer for pointing out this error which is now corrected.*

*Figure taken from Sadananadan et al. (3). Figure b highlighted shows wnt activity in stem-like (CMS4) and goblet-cell type (CMS3) subtype.*

3) line 195 - would also reference (Kunal Rai)

*Response: The reference has been included as advised.*

4) line 337 - need more description of these cohorts in main text: stage? Pre/post treatment? Primary or metastatic tumors?

*Response: The reviewer’s suggestion has well taken. The clinical information has been offered in Table 1.*

5) Figure 2A is confusing, does not seem to align with the caption. Legend does not describe the labels, which I think correspond to patients

*Response: We apologize for not providing a clear caption, and now have included an appropriate description.*

6) Figure 3E - too small to view in current format, would make larger

*Response: We apologize for not providing Figure 3E clearly. We now provide a magnified image and link to a high-resolution image on GitHub (*[*https://github.com/masoodlab/CRC-Single-Cell/blob/main/Figure_IHC_ASrev.pptx*](https://github.com/masoodlab/CRC-Single-Cell/blob/main/Figure_IHC_ASrev.pptx) *)*

Chowdhury, S., Hofree, M., Lin, K., Maru, D., Kopetz, S., and Shen, J.P. (2021). Implications of intratumor heterogeneity on consensus molecular subtype (CMS) in colorectal cancer. Cancers 13, 4923.

Laurent-Puig, P., Marisa, L., Ayadi, M., Blum, Y., Balogoun, R., Pilati, C., Le Malicot, K., Lepage, C., Emile, J.F., Salazar, R., et al. (2018). Colon cancer molecular subtype intratumoral heterogeneity and its prognostic impact: An extensive molecular analysis of the PETACC-8. Annals of Oncology 29, viii18.

Pelka, K., Hofree, M., Chen, J.H., Sarkizova, S., Pirl, J.D., Jorgji, V., Bejnood, A., Dionne, D., Ge, W.H., Xu, K.H., et al. (2021). Spatially organized multicellular immune hubs in human colorectal cancer. Cell 184, 4734-4752.e4720.

Piskol, R., Huw, L., Sergin, I., Kljin, C., Modrusan, Z., Kim, D., Kljavin, N., Tam, R., Patel, R., Burton, J., et al. (2019). A Clinically Applicable Gene-Expression Classifier Reveals Intrinsic and Extrinsic Contributions to Consensus Molecular Subtypes in Primary and Metastatic Colon Cancer. Clinical Cancer Research 25, 4431-4442.

Slyper, M., Porter, C.B.M., Ashenberg, O., Waldman, J., Drokhlyansky, E., Wakiro, I., Smillie, C., Smith-Rosario, G., Wu, J., Dionne, D., et al. (2020). A single-cell and single-nucleus RNA-Seq toolbox for fresh and frozen human tumors. Nature Medicine 26, 792-802.

*Response: We appreciate the reviewer's suggestions and, as requested, included them in the revised version of the manuscript.*

Reviewer #2:
In this manuscript, "Refining Colorectal Cancer Classification And Clinical Stratification Through A Single- Cell Atlas" Masood and collogues present a single-cell atlas of Colorectal Cancer (CRC). They profiled ~50k cells from 16 CRC tumours and eight adjacent normal tissues. A key finding of this manuscript includes the identification of fibroblast and macrophage populations and its implications in predicating CMS subtypes. In addition, they also report the association between a subset of CD8+ T-cells and its importance in predicting immunotherapy response in CMS1 (MSI-H) CRCs. In the last few years, multiple groups have reported the single-cell atlas of CRCs. This manuscript has integrated this information/data to increase the patient number and provide more generalised observations in CRCs.

*We would like to thank reviewer for reviewing our manuscript and providing excellent feedback and suggestions*.

Overall this is an interesting manuscript and provides some interesting observations, specifically potential targeting of CAFs and C1Q TAMs to improve IO response. However, the lack of experimental validation and suboptimal analysis of bulk RNA-seq data dampens this reviewer's enthusiasm. I suggest the author employ CIBERSORTx or other deconvolution tools to analyse bulk RNA-seq and identify why higher CAF and C1Q TAMs lead to poor outcomes (cell-cell interaction analysis).

*Response: Thank you for the excellent suggestion. We used CIBERSORTx and the results are consistent with our previous deconvolution work. The findings have been described, and figures have been included (Supplementary Figure S19, Figure S24, Figure S28, Figure S30 and Figure S34). Additionally, our initial analysis was based on a previously described method [6]. A recent study has also used a similar approach, assigning a gene signature score to each sample and computing the mean of the z-scores for each individual gene in the sample for B-cell identification [7] . Another study used similar approach followed by pathway enrichment for their analysis [8].*

*We also ran a continuous score model utilizing the cell-signature data from CIBERSORTx results, and the findings corroborated our hypothesis that CRC existed in the tumor stromal immune continuum (Supplementary Figure S30).*

For cell-cell interaction analysis reply, please see below.

Moreover, it is essential to validate these findings in retrospective patient samples (FFPE blocks) to substantiate these claims.

*Response: We appreciate the reviewer's comment and apologize for not presenting the data more clearly. We validated our results using two separate bulk transcriptomics studies and, as requested by reviewer 1, included multivariate analysis to further establish the relevance of CAF and TAMs in CRC biology. Additionally, we have analyzed independent single cell data from Pelka et al., that corroborate our findings.*

# *In sum, this study investigated four different CRC single cell datasets along with a CAF single-cell cohort of breast cancer (for comparative analysis) comprising of 487,829 cells and two bulk transcriptomics data sets of > 740 samples. We hope our results answer reviewers’ comment about validation.*

# Authors should also perform additional trajectory analysis such as CellRank (PMID: 35027767), which is more specific for cancer cells to compare Lee et al., and this manuscript's cell continuum observation.

# Response: We have used another independent cohort (Belgian cohort) for trajectory analysis further confirming our results (Figure 2C-2E) [9]. We also used Slingshot method for trajectory analysis that confirmed intratumoral heterogeneity of CMS beyond CMS classification consistent with monocle results (supplementary figure S10). Monocle and Slingshot uses two independent methods to build trajectory, monocle works by building tree from individual cells whereas slingshot builds on a minimum spanning tress designed for multiple branching lineages. CellRank was published a month ago and we have not yet optimized the pipeline, but we will utilize CellRank in our future studies.

# Authors have done a good job of including bulk RNA-seq in their analysis for validation etc.; however, their analysis methods are not very optimal and they should consider employing CYBERSORTx (PMID: 31061481) or EcoTyper (PMID: 34597589) to deconvolute bulk RNA-seq and validate scRNA-seq observations in bulk data.

# *Response: Please refer to the section above on the CIBERTSORTx analysis performed at the reviewer's request.*

It is interesting to see the correlation between CAFs, TAM and poor outcomes in CRC. It's not sure if these cells are spatially enriched in TME or CRC and what kind of immunosuppressive interactions occur between them, which might play an important role in IO response.

*Response: We agree with the reviewer's view that cell-cell interactions are crucial to our work and appreciate the reviewer's recommendation.*

# *Using CellPhoneDB, we uncovered key cell-cell interactions where we found bidirectional cross talk between CD8+ T-cells and C1Q+ TAMs whereby TAM’s led to T-cell dysfunction through multiple immune check point ligand interactions (CD40LG:CD40, TIGIT: NECTIN2, CD74: COPA, CD28:CD86, SIRPA:CD47, and CD86:CTLA4) whereas CD8+ T-cells lead to M2 like polarization of macrophages (*CD74:MIF, SIRPA:CD47*s) (Figure 8A-8B). CTLA4-mediated trans-endocytosis of co-stimulatory molecules CD86 from antigen-presenting cells (APCs) diminishes APCs' ability to co-stimulate T cells. These key findings in our analysis further explain how CD8+ T-cells and TAM’s interact in TME leading to immunosuppression in CRC. Additionally, we also describe various CAFs and their interaction with both C1Q+TAM and CD8+ T-cells leading to immunosuppression. All these findings further confirm our hypothesize that CAFs and TAM’s play key role in CRC pathology (Figure 8C-8F).*

# *References:*

1. Hornburg M, Desbois M, Lu S, Guan Y, Lo AA, Kaufman S, et al. Single-cell dissection of cellular components and interactions shaping the tumor immune phenotypes in ovarian cancer. Cancer Cell. 2021;39:928-944.e6.

2. Zhang Y, Narayanan SP, Mannan R, Raskind G, Wang X, Vats P, et al. Single-cell analyses of renal cell cancers reveal insights into tumor microenvironment, cell of origin, and therapy response. Proc Natl Acad Sci USA. 2021;118:e2103240118.

3. Sadanandam A, Lyssiotis CA, Homicsko K, Collisson EA, Gibb WJ, Wullschleger S, et al. A colorectal cancer classification system that associates cellular phenotype and responses to therapy. Nature Medicine. 2013;19:619–25.

4. Eason K, Sadanandam A. Molecular or Metabolic Reprograming: What Triggers Tumor Subtypes? Cancer Res. 2016;76:5195–200.

5. Miller SA, Ghobashi AH, O’Hagan HM. Consensus molecular subtyping of colorectal cancers is influenced by goblet cell content. Cancer Genetics. 2021;254–255:34–9.

6. Bartoschek M, Oskolkov N, Bocci M, Lövrot J, Larsson C, Sommarin M, et al. Spatially and functionally distinct subclasses of breast cancer-associated fibroblasts revealed by single cell RNA sequencing. Nature Communications. 2018;9:5150.

7. Patil NS, Nabet BY, Müller S, Koeppen H, Zou W, Giltnane J, et al. Intratumoral plasma cells predict outcomes to PD-L1 blockade in non-small cell lung cancer. Cancer Cell. 2022;S1535610822000356.

8. Nicolas AM, Pesic M, Engel E, Ziegler PK, Diefenhardt M, Kennel KB, et al. Inflammatory fibroblasts mediate resistance to neoadjuvant therapy in rectal cancer. Cancer Cell. 2022;40:168-184.e13.

9. Lee H-O, Hong Y, Etlioglu HE, Cho YB, Pomella V, Van den Bosch B, et al. Lineage-dependent gene expression programs influence the immune landscape of colorectal cancer. Nature Genetics. 2020;52:594–603.

**Second round of review**

**Reviewer 1**

All of my prior concerns have been addressed. I think this is an important paper given it's findings have implications for advancing idea of precision medicine in colon cancer. The authors should be commended for making data and full analysis / methods available on github. That will increase the value of this paper as a resource

**Reviewer 2**

Authors have addressed most of my comments in the revised version. I recommend acceptance of this manuscript in Genome Biology.
